# Synthesis of benzo[*b*]furans from alkynyl sulfoxides and phenols by the interrupted Pummerer reaction†

**DOI:** 10.1039/d2ra07856b

**Published:** 2023-01-03

**Authors:** Akihiro Kobayashi, Tsubasa Matsuzawa, Takamitsu Hosoya, Suguru Yoshida

**Affiliations:** Department of Biological Science and Technology, Faculty of Advanced Engineering, Tokyo University of Science 6-3-1 Niijuku Katsushika-ku Tokyo 125-8585 Japan s-yoshida@rs.tus.ac.jp; Laboratory of Chemical Bioscience, Institute of Biomaterials and Bioengineering, Tokyo Medical and Dental University (TMDU) 2-3-10 Kanda-Surugadai Chiyoda-ku Tokyo 101-0062 Japan

## Abstract

The interrupted Pummerer reaction of alkynyl sulfoxides with phenols is disclosed. A wide range of benzo[*b*]furans were efficiently synthesized through unexplored electrophilic activation of the electron-deficient alkynyl sulfinyl group. Based on the good availability of alkynyl sulfoxides, we successfully prepared various functionalized benzo[*b*]furans from readily available alkynes, thiosulfonates, and phenols.

## Abstract 2

The interrupted Pummerer reaction of alkynyl sulfoxides with phenols is disclosed. The good availability of alkynyl sulfoxides and phenols enabled us to prepare various functionalized benzo[*b*]furans.

## Graphical abstract

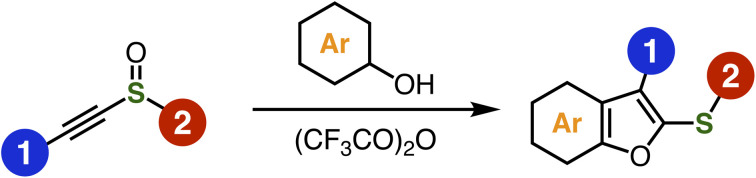

## Introduction

**Fig. 1:**
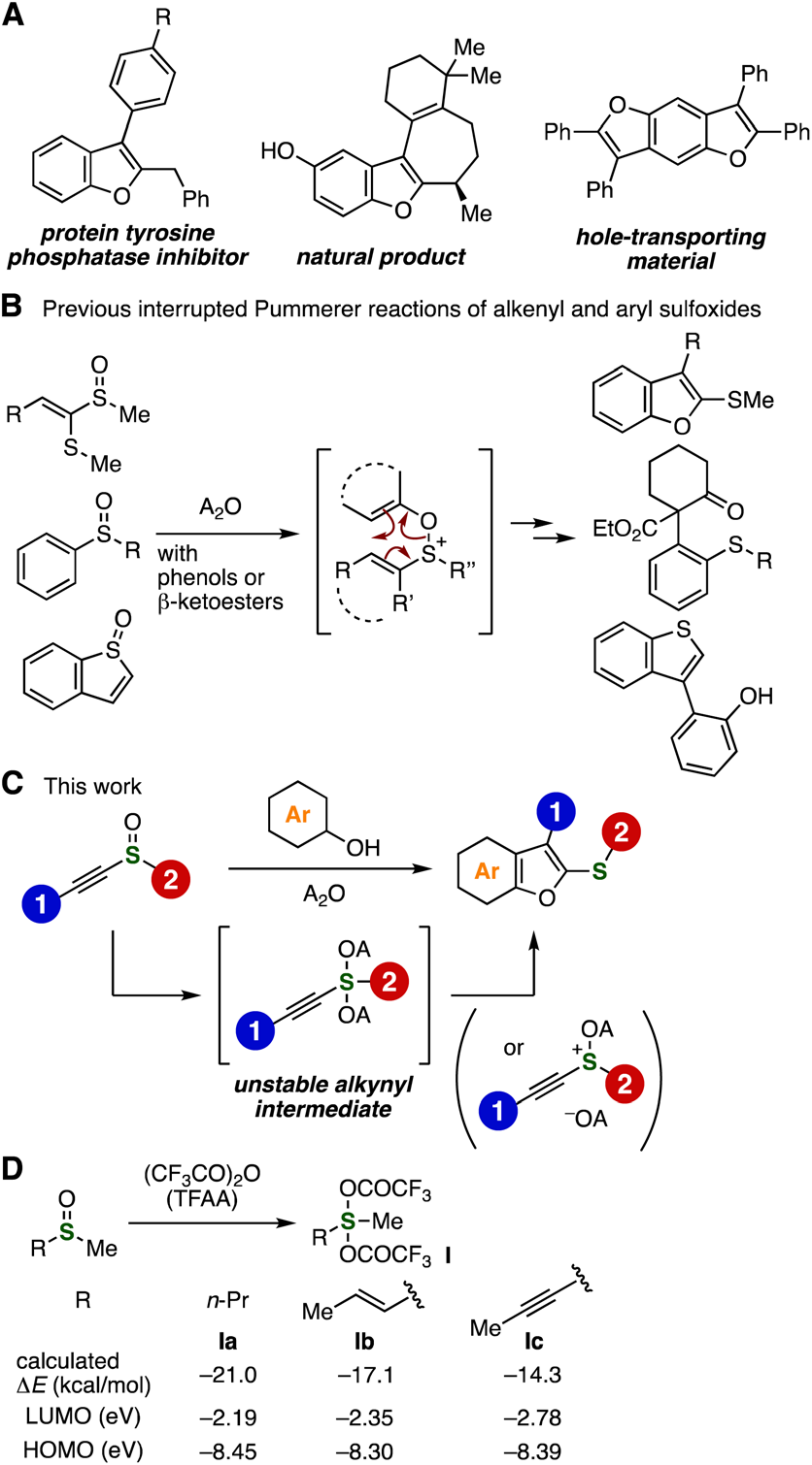
Backgrounds and an abstract of this study. (A) Significant benzofurans. (B) Interrupted Pummerer reactions. (C) This work. (D) DFT calculations of sulfuranes Ia–Ic. Δ*E* = *E*(sulfuranes) − *E*(sulfoxides) − *E*(TFAA). See ESI† for details.

Benzo[*b*]furan scaffolds are of great importance in a wide range of research fields including pharmaceutical sciences, natural product chemistry, and materials chemistry (Fig. 1A).^1^ Various methods to synthesize benzofurans have been developed so far. For example, *O*-alkylation of salicylaldehyde derivatives with chloroacetic acid and subsequent cyclization affords a range of benzofurans.^2^ Despite the significance of benzofurans, the synthesis of highly functionalized benzofurans is not easy by conventional methods due to limitations in the benzofuran skeleton construction. We herein describe a new method to prepare multisubstituted benzofurans from alkynyl sulfoxides and phenols *via* the interrupted Pummerer reaction.

The interrupted Pummerer reactions are emerging methods to synthesize highly functionalized organosulfur compounds from sulfoxides by the electrophilic activation of S=O bonds (Fig. 1B).^3^ Recently, several unique transformations of a range of alkenyl and aryl sulfoxides with various nucleophiles have been achieved through the electrophilic activation of the sulfoxide moieties followed by smooth charge-accelerated [3,3]-sigmatropic rearrangement.^4,5^ In contrast, interrupted Pummerer reactions of alkynyl sulfoxides have not been developed to the best of our knowledge, which may be due to the electron-deficient nature of the sulfoxide moiety by the electron-withdrawing sp-hybridized carbon. We conceived that the interrupted Pummerer reaction of alkynyl sulfoxides with phenols with the appropriate activators will allow us to synthesize a wide variety of functionalized benzofurans owing to the good availability of alkynyl sulfoxides and phenols (Fig. 1C).

Before examining the benzofuran synthesis, we evaluated the stability of alkynyl sulfurane intermediate Ic compared to alkenyl and alkyl sulfuranes Ia and Ib by the DFT calculation (Fig. 1D). The calculated energy differences between sulfoxides with trifluoroacetic anhydride (TFAA) and sulfuranes Ia–Ic showed that the electron-deficient alkynyl sulfurane Ic is unstable in comparison with alkyl and alkenyl sulfuranes Ia and Ib. These results clearly show that stability of sulfurane Ic was decreased by the significant electron-deficiency of alkynyl carbons. Comparing LUMO energies of Ia–Ic suggests higher electrophilicity of alkynyl sulfurane Ic than that of alkyl and alkenyl sulfuranes Ia and Ib.

## Results and discussion

After screening the reaction conditions, we established the synthetic method of benzofuran 3a from phenol (1a) and alkynyl sulfoxide 2a through electrophilic sulfoxide activation (Table 1). While benzofuran 3a was not obtained when acetic anhydride was used as an activator (entry 1), we found that treating a mixture of phenol (1a) and alkynyl sulfoxide 2a with triflic anhydride (Tf_2_O) afforded 3-butyl-2-(ethylthio)benzo[*b*]furan (3a) in moderate yield (entry 2). The yield was improved by the addition of 2,6-di(*tert*-butyl)pyridine as a base (entry 3).^6^ We accomplished the synthesis of benzofuran 3a from 1a and 2a in dichloromethane with TFAA in excellent yield (entry 4).^7^ Benzofuran 3a was also prepared in 1 mmol scale without decreasing the yield, showing the good scalability of the protocol (entry 5). Although we failed the synthesis of benzofuran 3a when using 2,6-di(*tert*-butyl)pyridine or triethylamine as an additive (entries 6 and 7), benzofuran 3a was also obtained in the presence of sodium carbonate (entry 8). Among solvents examined (entries 4 and 9–12), dichloromethane, toluene, and α,α,α-trifluorotoluene were effective for benzofuran synthesis (entries 4, 11, and 12). Trifluoroacetic acid did not activate sulfoxide 2a (entry 13).

**Table 1:** Screening of the reaction conditions

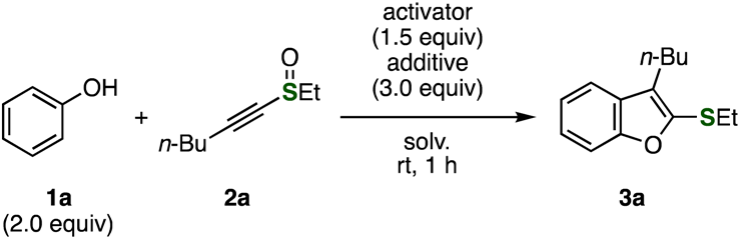				
Entry	Activator	Additive	Solv.	Yield^a^ (%)
1	Ac_2_O	None	CH_2_Cl_2_	0
2	Tf_2_O	None	CH_2_Cl_2_	45
3	Tf_2_O	2,6-(*t*-Bu)_2_pyridine	CH_2_Cl_2_	81
4	TFAA	None	CH_2_Cl_2_	97 (94)^b^
5	TFAA	None	CH_2_Cl_2_	95^c^
6	TFAA	2,6-(*t*-Bu)_2_pyridine	CH_2_Cl_2_	0
7	TFAA	NEt_3_	CH_2_Cl_2_	0
8	TFAA	Na_2_CO_3_	CH_2_Cl_2_	89
9	TFAA	None	MeCN	67
10	TFAA	None	THF	0
11	TFAA	None	Toluene	85
12	TFAA	None	PhCF_3_	91
13	CF_3_CO_2_H	None	CH_2_Cl_2_	0

a ^1^H NMR yield.

b Isolated yield (0.1 mmol scale).

c Isolated yield (1 mmol scale).

**Fig. 2:**
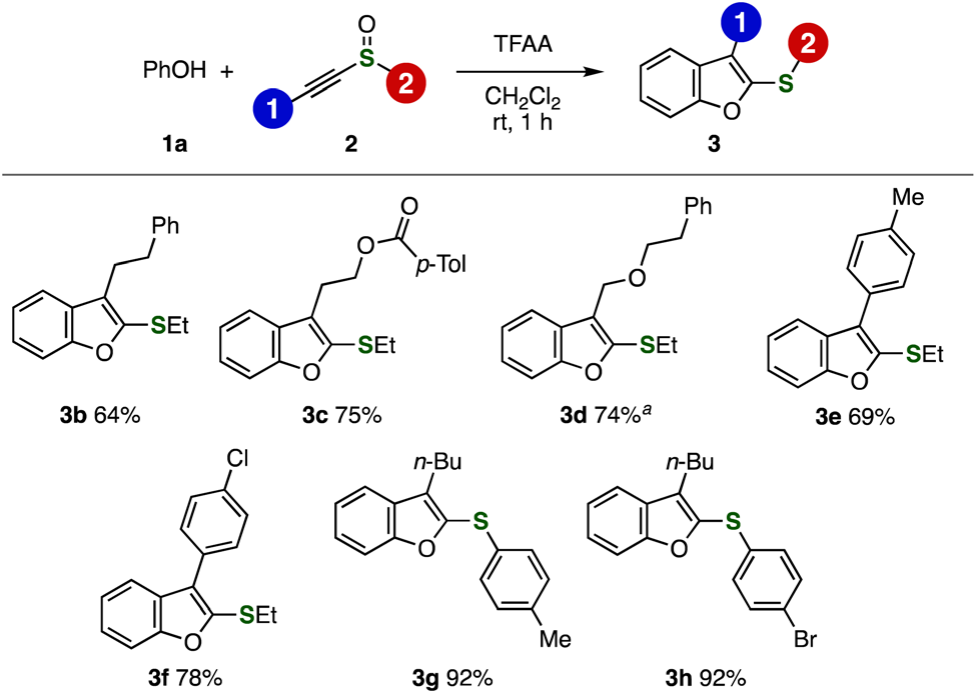
Syntheses of benzofurans 3 using various alkynyl sulfoxides 2. See the ESI† for details. ^*a*^The reaction was performed with Tf_2_O and 2,6-di(*tert*-butyl)pyridine.

With optimized conditions in hand, a wide range of 2-sulfanylbenzofurans 3 were synthesized from phenol (1a) and various alkynyl sulfoxides 2 (Fig. 2). For example, phenethyl-substituted benzofuran 3b was synthesized in good yield. It is worthy to note that an ester moiety was tolerated under the conditions, providing benzofuran 3c in high yield. We succeeded in the synthesis of ether-tethered benzofuran 3d by electrophilic activation with Tf_2_O in the presence of 2,6-di(*tert*-butyl)pyridine in good yield, where decomposition took place when the reaction was conducted with TFAA. Benzofurans 3e and 3f having aryl groups at 3-position were prepared efficiently without damaging 4-tolyl and 4-chlorophenyl groups. Also, alkynyl aryl sulfoxides participated to the benzofuran synthesis affording 3g and 3h bearing 4-tolylthio and 4-bromophenylthio groups in high yields. Since a wide variety of alkynyl sulfoxides were easily available from terminal alkynes or alkynylsilanes,^8^ the broad scope of the benzofuran synthesis is a great advantage over previous reports.^4^

**Fig. 3:**
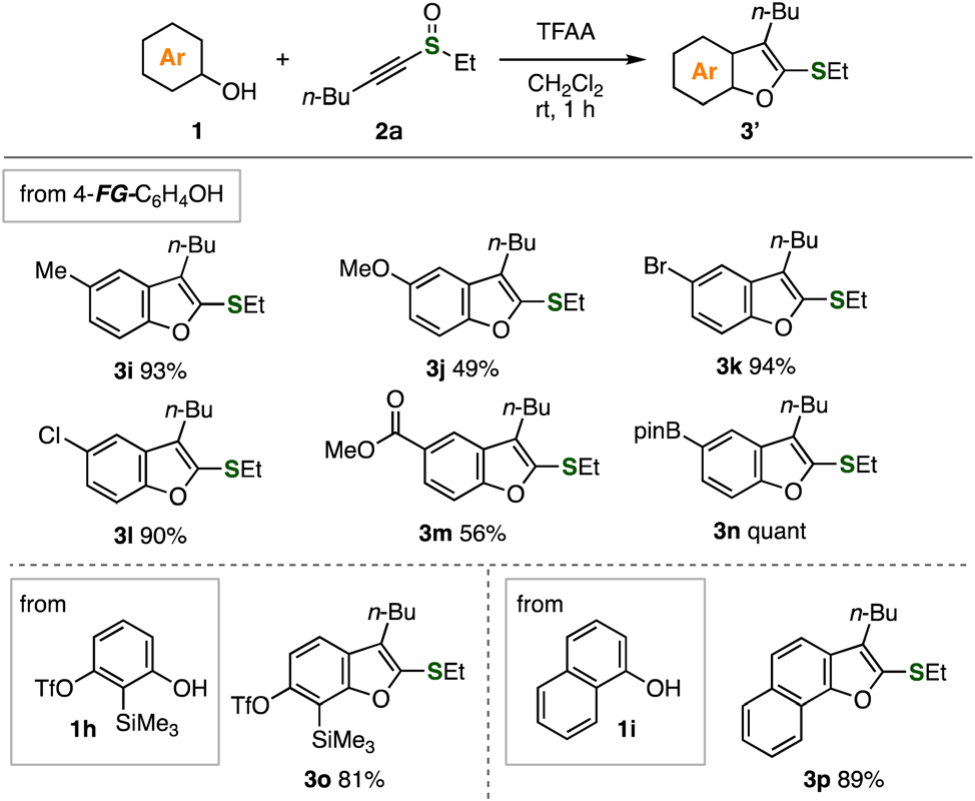
Syntheses of benzofurans 3 using various phenols. See the ESI† for details.

Diverse functionalized benzofurans were successfully synthesized from alkynyl sulfoxide 2a and a broad variety of phenols (Fig. 3). Indeed, phenols having methyl, methoxy, bromo, chloro, and methoxycarbonyl groups served in the benzofuran synthesis with alkynyl sulfoxide 2a in moderate to high yields keeping the functional groups unreacted. Furthermore, the reaction of 2-trimethylsilyl-3-triflyloxyphenol (1h) with alkynyl sulfoxide 2a efficiently proceeded to furnish benzofuran 3o leaving butyl, ethylthio, trimethylsilyl, and triflyloxy groups untouched. In addition, naphthofuran 3p was synthesis from 1-naphthol (1i) in good yield.

**Fig. 4:**
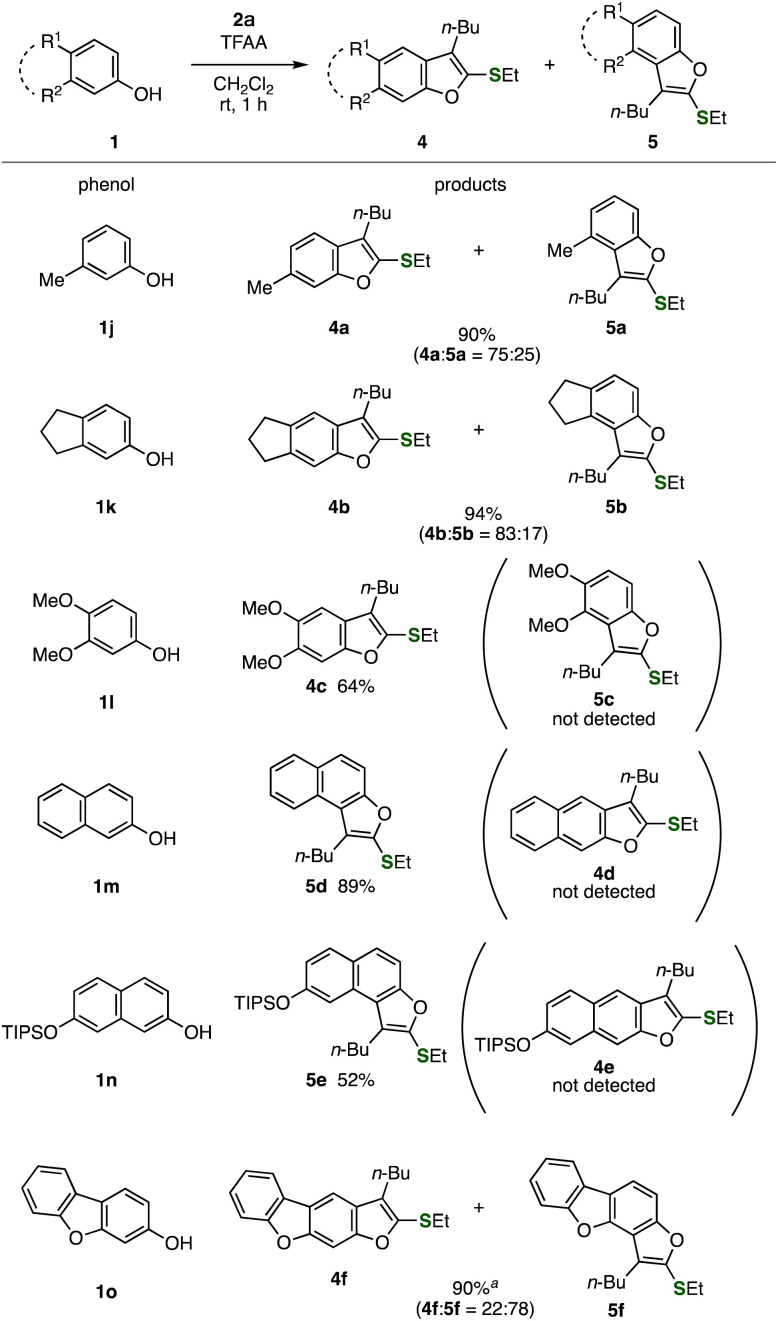
Syntheses of benzofurans 4 and 5 using various phenols. See the ESI† for details. ^*a* 1^H NMR yield. Authentic samples (4f 7%; 5f 65%) were isolated respectively.

We then examined the regioselectivity in the benzofuran synthesis (Fig. 4). When the benzofuran synthesis was performed using *m*-cresol, 6-methyl-substituted benzofuran 4a was majorly obtained along with 4-methylbenzofuran 5a with moderate regioselectivity, clearly showing that the C–C bond formation at the unhindered site was favorable. Benzofuran 4b was also synthesized as a major product with good regioselectivity when using 5-hydroxyindane (1k). Of note, we succeeded in the preparation of benzofuran 4c as a sole product, in which the C–C bond formation at the vacant position took place selectively and regioisomer 5c was not observed. In contrast, naphthols 1m and 1n reacted with alkynyl sulfoxide 2a at the hindered site affording naphthofurans 5d and 5e selectively without forming regioisomers 4d and 4e, where cyclization took place at more electron-rich carbons.^9^ Moreover, we achieved the synthesis of benzofuran-fused benzofurans 4f and 5f in good yields, in which C–C bond formation at 4-position occurred primarily in moderate selectivity.

**Fig. 5:**
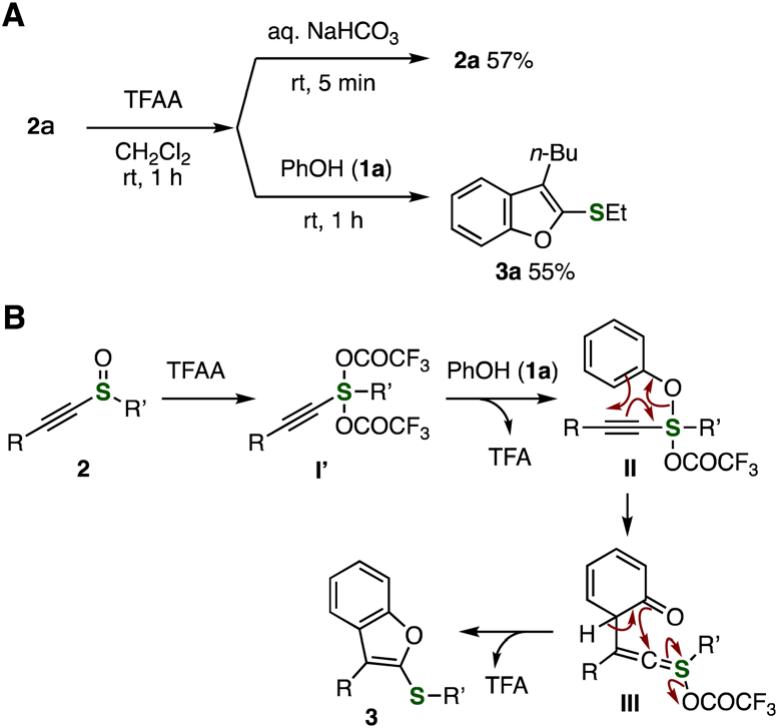
Control experiments and reaction mechanism. (A) The reaction of alkynyl sulfoxide 2a with TFAA. (B) Plausible reaction mechanism. See the ESI† for details.

To clarify the reaction mechanism of the benzofuran formation, we conducted control experiments (Fig. 5A). In order to examine the stability of intermediates generated *in situ* by the electrophilic activation of sulfoxides, we performed the addition of aqueous sodium bicarbonate or phenol (1a) after the prior activation of alkynyl sulfoxide 2a with TFAA in dichloromethane for 1 h at room temperature (Fig. 5A). As a result, sulfoxide 2a or benzofuran 3a was respectively obtained through the hydrolysis or the reaction with phenol (1a) in slightly decreased yields, suggesting that side reactions such as the Pummerer rearrangement did not take place smoothly even in the absence of phenols.^10^

A plausible reaction mechanism is shown in Fig. 5B. First, the formation of sulfurane intermediates I′ from alkynyl sulfoxides 2 by the electrophilic activation with TFAA followed by the nucleophilic substitution with 1a would afford intermediates II.^3^ Because side-products by the C–C bond formation of activated alkynyl sulfoxides I′ with 1a at *para*-position were not observed,^11^ smooth S–O bond formation providing intermediates II would take place as previously reported interrupted Pummerer reactions.^4^ Then, the sigmatropic rearrangement of alkynyl sulfuranes II and subsequent deprotonation lead to benzofurans 3.^12^

**Fig. 6:**
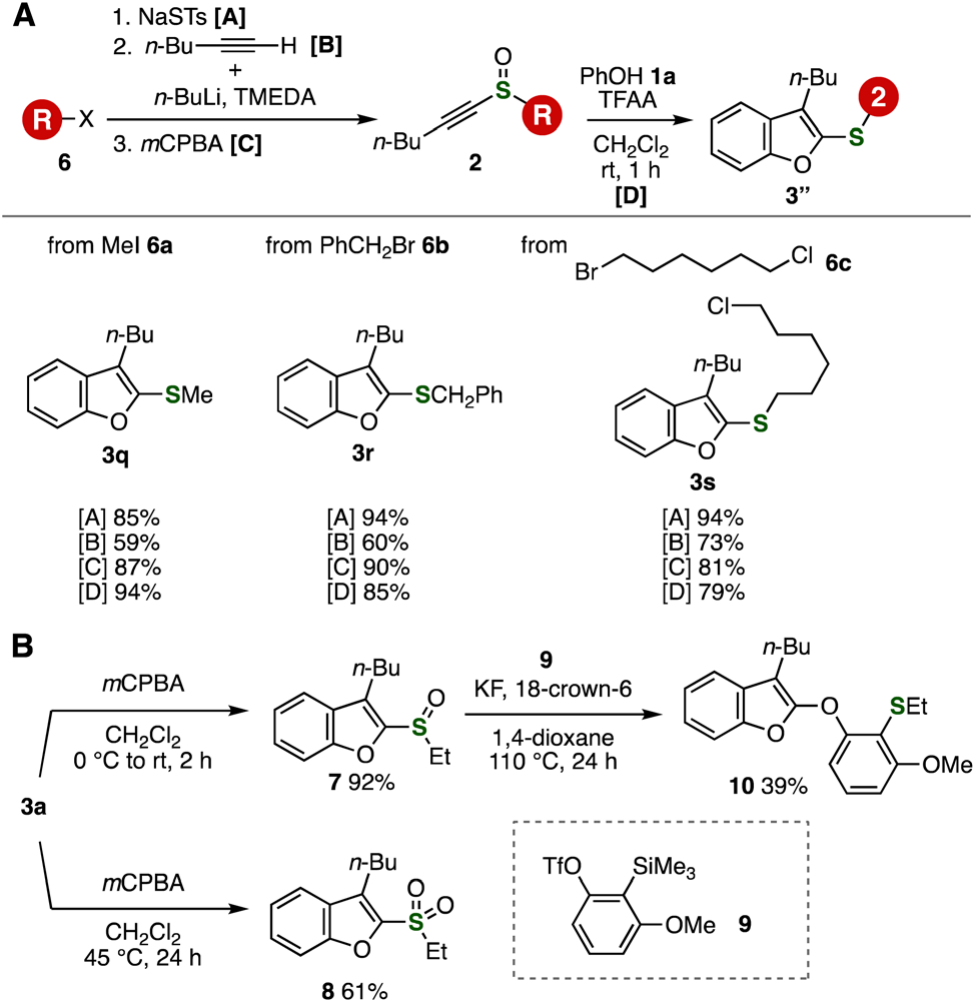
(A) Benzofuran synthesis from alkyl halides. (B) Synthesis of organosulfur compounds from 3a.

Then, we showcased the benefits of the benzofuran synthesis from alkynyl sulfoxides and phenols (Fig. 6). A variety of benzofurans 3q–3s were efficiently synthesized from alkyl halides 6a–6c, sodium thiosulfonate, 1-hexyne, and phenol since sodium thiosulfonate worked as an “^+^S^−^” equivalent (Fig. 6A). Indeed, the preparation of alkynyl sulfides by *S*-alkylation and *S*-alkynylation followed by *S*-oxidation and the benzofuran formation allowed us to access easily functionalized benzofurans by the four-step four-component coupling protocols in a modular synthetic manner.

The good transformability of the sulfanyl group served in synthesizing a wide range of benzofurans (Fig. 6B). For example, we succeeded in the preparation of sulfoxide 7 and sulfone 8 by *S*-oxidation of 3a. Since a variety of transformations of the sulfinyl groups can be accomplished, the benzofuran formation and following *S*-oxidation and subsequent transformations such as aryne reactions^13,14^ realize the synthesis of highly functionalized benzofurans. Indeed, the migratory oxythiolation of 3-methoxybenzyne from *o*-silylaryl triflate 9 and sulfoxide 7 with potassium fluoride and 18-crown-6 in 1,4-dioxane at 110 °C took place smoothly to provide highly functionalized benzofuran 10*via* the C–S and two C–O bond formations, in which the migration of the 3-butylbenzofuran-2-yl group selectively proceeded in the C–O bond formation.^14^

## Conclusions

In conclusion, we found a new method to synthesize benzo[*b*]furans from alkynyl sulfoxides and phenols by the electrophilic activation of the electron-deficient alkynyl sulfinyl moiety. Owing to the ready availability of alkynyl sulfoxides, the efficient synthetic method enabled us to prepare a wide range of functionalized benzofurans having sulfanyl groups. Since organosulfur substituents are easily transformed into various functional groups, the benzofuran synthesis will serve in developing diverse bioactive molecules. Further studies such as theoretical calculations are ongoing in our laboratory.

## Conflicts of interest

There are no conflicts to declare.

## Supplementary Material

RA-013-D2RA07856B-s001
