# Supplementary material for: Synthesis of benzo[b]furans from alkynyl sulfoxides and phenols by the interrupted Pummerer reaction
Source: RSC Adv. 2023 Jan 3;13(2):839–43. doi: 10.1039/d2ra07856b (PMC9809539; doi:10.1039/d2ra07856b)

Electronic Supplementary Material (ESI) for RSC Advances.
This journal is © The Royal Society of Chemistry 2022

Supporting Information

Synthesis of benzo[*b*]furans from alkynyl sulfoxides and phenols by the interrupted Pummerer reaction

Akihiro Kobayashi,[a,b] Tsubasa Matsuzawa,[b] Takamitsu Hosoya[b] and Suguru Yoshida[a]*

[a]*Department of Biological Science and Technology, Faculty of Advanced Engineering, Tokyo University of Science, 6-3-1 Niijuku, Katsushika-ku Tokyo 125-8585*
[b]*Laboratory of Chemical Bioscience, Institute of Biomaterials and Bioengineering, Tokyo Medical and Dental University (TMDU), 2-3-10 Kanda-Surugadai, Chiyoda-ku, Tokyo 101-0062, Japan*

Contents

General Information S1
Structures of Phenols 1 and Alkynyl Sulfoxides 2 S2
Experimental Procedures S3
Mechanistic Studies S7
Computational Methods S8
Characterization Data of New Compounds S13
References for Supporting Information S22
^{1}H and ^{13}C NMR Spectra of Compounds S23

General Information

All reactions were performed with dry glassware under atmosphere of argon, unless otherwise noted. Analytical thin-layer chromatography (TLC) was performed on precoated (0.25 mm) silica-gel plates (Merck Chemicals, Silica Gel 60 F254, Cat. No. 1.05715). Column chromatography was conducted using silica-gel (Kanto Chemical Co., Inc., Silica Gel 60N, spherical neutral, particle size 40–50 μm, Cat. No. 37562-85 or particle size 63–210 μm, Cat. No. 37565-85). Preparative TLC (PTLC) was performed on silica gel (Wako Pure Chemical Industries Ltd., Wakogel B-5F, Cat. No. 230-00043). Melting points (Mp) were measured on an OptiMelt MPA100 (Stanford Research Systems), and are uncorrected. ^{1}H NMR spectra were obtained with a Bruker AVANCE 400 spectrometer at 400 MHz. ^{13}C NMR spectra were obtained with a Bruker AVANCE 400 spectrometer at 101 MHz. ^{19}F NMR spectra were obtained with a Bruker AVANCE 400 spectrometer at 376 MHz. All NMR measurements were carried out at 25 ºC. $CDCl_3$ (Kanto Chemical Co. Inc., Cat. No. 07663-23) was used as a solvent for obtaining NMR spectra. Chemical shifts (δ) are given in parts per million (ppm) downfield from the solvent peak (δ 7.26 for ^{1}H NMR in $CDCl_3$, δ 77.0 for ^{13}C NMR in $CDCl_3$) as an internal reference with coupling constants (*J*) in hertz (Hz). The abbreviations s, d, t, q, and m signify singlet, doublet, triplet, quartet, and multiplet, respectively. High-resolution mass spectra (HRMS) were measured on a JEOL JMS-T100CS "AccuTOF CS" mass spectrometer under positive electrospray ionization (ESI^{+}) conditions or JMS-700 (JEOL, Tokyo, Japan) mass spectrometer under electron impact ionization (EI) conditions.

Unless otherwise noted, materials obtained from commercial suppliers were used without further purification. (2-(prop-2-yn-1-yloxy)ethyl)benzene,[S1] but-3-yn-1-ylbenzene,[S2] *S*-(4-tolyl) 4-methylbenzenesulfonothioate,[S3] *S*-(4-bromophenyl) 4-methylbenzenesulfonothioate,[S3] ethyl (4-tolylethynyl) sulfide,[S4] ((4-chlorophenyl)ethynyl)(ethyl)sulfane,[S4] ethyl(4-phenylbut-1-yn-1-yl)sulfane,[S4] 3-hydroxy-2-(trimethylsilyl)phenyl trifluoromethanesulfonate (**1h**),[S6] and 7-((triisopropylsilyl)oxy)naphthalen-2-ol (**1n**)[S5] were prepared according to the reported methods.

Structures of Phenols 1 and Alkynyl Sulfoxides 2

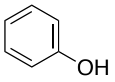

1a

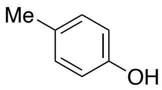

1b

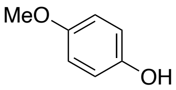

1c

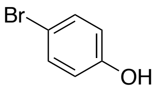

1d

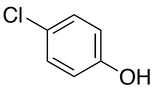

1e

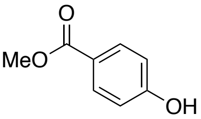

1f

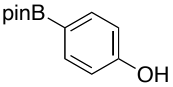

1g

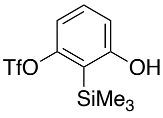

1h

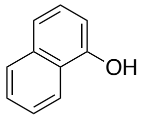

1i

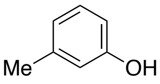

1j

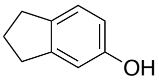

1k

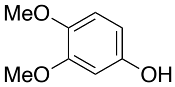

1l

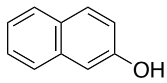

1m

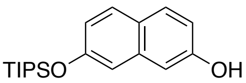

1n

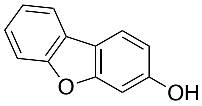

1o

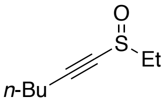

2a

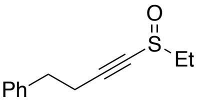

2b

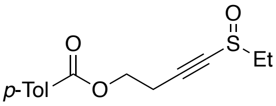

2c

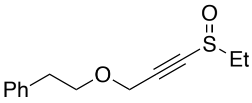

2d

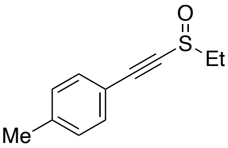

2e

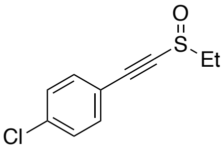

2f

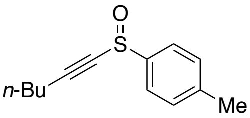

2g

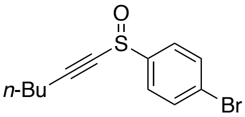

2h

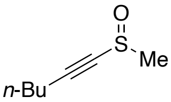

2i

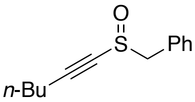

2j

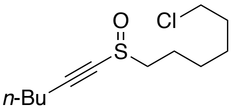

2k

Experimental Procedures

A typical procedure for the synthesis of benzofurans

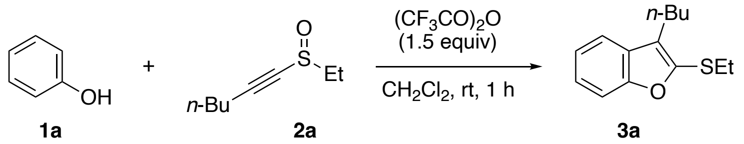

To a mixture of phenol (**1a**) (18.8 mg, 0.200 mmol, 2.0 equiv) and 1-(ethylsulfinyl)hex-1-yne (**2a**) (15.7 mg, 99.4 μmol) in dichloromethane (1.0 mL) was added trifluoroacetic anhydride (20.9 μL, 0.150 mmol, 1.5 equiv) at room temperature. After stirring for 1 h at the same temperature, to the mixture was added an aqueous saturated solution of sodium bicarbonate (5 mL) and the mixture was extracted with dichloromethane (15 mL × 3). The combined organic extract was washed with brine (20 mL) and dried (Na_2SO_4). After filtration, the filtrate was concentrated under reduced pressure. The residue was purified by preparative TLC (*n*-hexane/ CH_2Cl_2 = 3/1) to give 3-butyl-2-(ethylthio)benzo[*b*]furan (**3a**) (21.9 mg, 93 μmol, 94%) as a colorless oil.

According to the procedure for preparing 3-butyl-2-(ethylthio)benzo[*b*]furan (**3a**), 2-(ethylthio)-3-phenethylbenzo[*b*]furan (**3b**), 2-(2-(ethylthio)benzo[*b*]furan-3-yl)ethyl 4-methylbenzoate (**3c**), 2-(ethylthio)-3-(phenethoxymethyl)benzo[*b*]furan (**3d**), 2-(ethylthio)-3-(4-tolyl)benzo[*b*]furan (**3e**), 3-(4-chlorophenyl)-2-(ethylthio)benzo[*b*]furan (**3f**), 3-butyl-2-(4-tolylthio)benzo[*b*]furan (**3g**), 2-((4-bromophenyl)thio)-3-butylbenzo[*b*]furan (**3h**), 3-butyl-2-(ethylthio)-5-methylbenzo[*b*]furan (**3i**), 3-butyl-2-(ethylthio)-5-methoxybenzo[*b*]furan (**3j**), 5-bromo-3-butyl-2-(ethylthio)benzo[*b*]furan (**3k**), 3-butyl-5-chloro-2-(ethylthio)benzo[*b*]furan (**3l**), methyl 3-butyl-2-(ethylthio)benzo[*b*]furan-5-carboxylate (**3m**), 2-(3-butyl-2-(ethylthio)benzo[*b*]furan-5-yl)-4,4,5,5-tetramethyl-1,3,2-dioxaborolane (**3n**), 3-butyl-2-(ethylthio)-7-(trimethylsilyl)benzo[*b*]furan-6-yl trifluoromethanesulfonate (**3o**), 3-butyl-2-(ethylthio)naphtho[1,2-*b*]furan (**3p**), 3-butyl-2-(methylthio)benzo[*b*]furan (**3q**), 2-(benzylthio)-3-butylbenzo[*b*]furan (**3r**), 3-butyl-2-((6-chlorohexyl)thio)benzo[*b*]furan (**3s**), 3-butyl-2-(ethylthio)-6-methylbenzo[*b*]furan (**4a**), 3-butyl-2-(ethylthio)-4-methylbenzo[*b*]furan (**5a**), 3-butyl-2-(ethylthio)-6,7-dihydro-5*H*-indeno[5,6-*b*]furan (**4b**), 1-butyl-2-(ethylthio)-7,8-dihydro-6*H*-indeno[5,4-*b*]furan (**5b**), 3-butyl-2-(ethylthio)-5,6-dimethoxybenzo[*b*]furan (**4c**), 1-butyl-2-(ethylthio)naphtho[2,1-*b*]furan (**5d**), ((1-butyl-2-(ethylthio)naphtho[2,1-*b*]furan-8-yl)oxy)triisopropylsilane (**5e**), 2-ethylthio-3-butylbenzofuro[6,5-*b*]benzofuran (**4f**), and 2-ethylthio-3-butylbenzo[*b*]furo[2,3-*g*]benzofuran (**5f**) were prepared from the corresponding phenols and alkynyl sulfoxides.

*Synthesis of 2-(ethylthio)-3-(phenethoxymethyl)benzofuran (**3d**)*

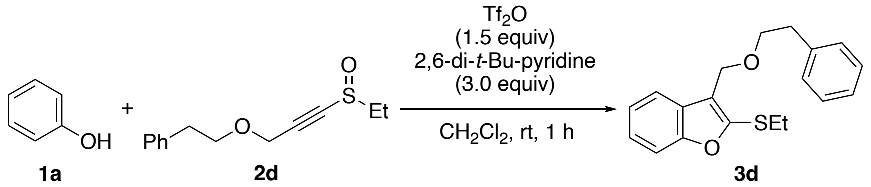

To a mixture of phenol (**1a**) (9.4 mg, 0.10 mmol, 2.0 equiv), (2-((3-(ethylsulfinyl)prop-2-yn-1-yl)oxy)ethyl)benzene (**2d**) (11.7 mg, 49.6 μmol) and 2,6-di-*tert*-butylpyridine (33.7 μL, 0.156 mmol, 3.0 equiv) in dichloromethane (0.50 mL) was added trifluoroacetic anhydride (20.9 μL, 0.150 mmol, 1.5 equiv) at room temperature. After stirring for 1 h at the same temperature, to the mixture was added an aqueous saturated solution of sodium bicarbonate (5 mL). The mixture was extracted with dichloromethane (15 mL × 3). The combined organic extract was washed with brine (20 mL) and dried (Na_2SO_4). After filtration, the filtrate was concentrated under reduced pressure. The residue was purified by preparative TLC (*n*-hexane/CH_2Cl_2 = 3/1) to give 2-(ethylthio)-3-(phenethoxymethyl)benzofuran (**3d**) (11.4 mg, 36.5 μmol, 74%) as a colorless oil.

A typical procedure for the synthesis of thiosulfonates from alkyl halides and sodium thiosulfonate

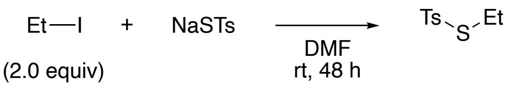

To a solution of sodium 4-methylbenzenesulfonothioate (6.30 g, 30.0 mmol) in DMF (90 mL) was added iodoethane (4.82 mL, 60.0 mmol) at room temperature. After stirring for 48 h at room temperature, to this was

added water (150 mL). The mixture was extracted with *n*-hexane/EtOAc = 4/1 (50 mL × 3), and the combined organic extract was washed with brine (50 mL) and dried (Na_2SO_4). After filtration, the filtrate was concentrated under reduced pressure. The residue was purified by column chromatography (silica-gel 90 g, *n*-hexane/EtOAc = 3/1) to give *S*-ethyl 4-methylbenzenesulfonothioate (6.42 g, 29.7 mmol, 99%) as a colorless solid.

According to the procedure for preparing *S*-ethyl 4-methylbenzenesulfonothioate, *S*-methyl 4-methylbenzenesulfonothioate, *S*-benzyl 4-methylbenzenesulfonothioate, and *S*-(6-chlorohexyl) 4-methylbenzenesulfonothioate were prepared from the corresponding alkyl halides and sodium thiosulfonate.

A typical procedure for the synthesis of alkynyl sulfides from hex-1-yne and thiosulfonates [S7]

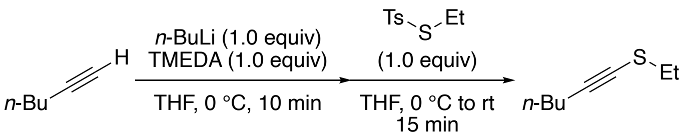

To a mixture of 1-hexyne (614 mg, 7.48 mmol) and *N,N,N',N'*-tetramethylethylenediamine (TMEDA) (867 mg, 7.46 mmol, 1.0 equiv) dissolved in THF (15 mL) was added *n*-BuLi (0.98 M in *n*-hexane, 7.6 mL, 7.5 mmol, 1.0 equiv) at 0 °C. After stirring for 10 min at the same temperature, to the mixture was added *S*-ethyl 4-methylbenzenesulfonothioate (1.61 g, 7.46 mmol, 1.0 equiv) dissolved in THF (7 mL). After stirring for 10 min at the same temperature, the mixture was allowed to warm to room temperature. After stirring for 15 min at room temperature, the mixture was concentrated under reduced pressure. The residue was dissolved in EtOAc (30 mL), washed with water (10 mL) and brine (10 mL), and dried (Na_2SO_4). After filtration, the filtrate was concentrated under reduced pressure. The residue was purified by flash column chromatography (silica-gel 35 g, *n*-hexane) to give ethyl hex-1-yn-1-yl sulfide (696 mg, 4.89 mmol, 66%) as a colorless oil.

According to the procedure for preparing ethyl hex-1-yn-1-yl sulfide, hex-1-yn-1-yl 4-tolyl sulfide, 4-bromophenyl hex-1-yn-1-yl sulfide, 4-(ethylthio)but-3-yn-1-ol (*n*-BuLi (2.0 equiv) and TMEDA (2.0 equiv) were used), hex-1-yn-1-yl methyl sulfide, benzyl hex-1-yn-1-yl sulfide, and 6-chlorohexyl hex-1-yn-1-yl sulfide were prepared from the corresponding hex-1-yne and thiosulfonates.

A typical procedure for the preparation of sulfoxides from sulfides [S8]

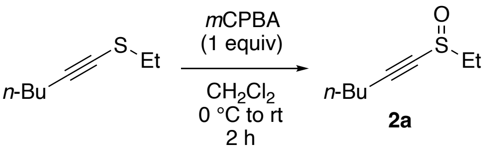

To a solution of ethyl hex-1-yn-1-yl sulfide (695 mg, 4.89 mmol) in CH_2Cl_2 (25 mL) was slowly added *m*-chloroperbenzoic acid (*m*CPBA) (ca. 77%, 1.10 g, ca. 4.9 mmol, ca. 1 equiv) at 0 °C. After stirring for 10 min at the same temperature, the mixture was allowed to warm to room temperature. After stirring for 2 h at room temperature, to the mixture was added an aqueous saturated solution of potassium carbonate (5 mL) and an aqueous saturated solution of sodium thiosulfate (5 mL). The mixture was extracted with CH_2Cl_2 (10 mL × 3). The combined organic extract was washed with brine (10 mL) and dried (Na_2SO_4). After filtration, the filtrate was concentrated under reduced pressure. The residue was purified by flash column chromatography (silica-gel 16 g, *n*-hexane/EtOAc = 2/1) to give 1-(ethylsulfinyl)hex-1-yne (**2a**) (666 mg, 4.21 mmol, 86%) as a colorless oil.

According to the procedure for preparing 1-(ethylsulfinyl)hex-1-yne (**2a**), (4-(ethylsulfinyl)but-3-yn-1-yl)benzene (**2b**), 1-((ethylsulfinyl)ethynyl)-4-methylbenzene (**2e**), 1-chloro-4-((ethylsulfinyl)ethynyl)benzene (**2f**), 1-(hex-1-yn-1-ylsulfinyl)-4-methylbenzene (**2g**), 1-bromo-4-(hex-1-yn-1-ylsulfinyl)benzene (**2h**), 1-(methylsulfinyl)hex-1-yne (**2i**), ((hex-1-yn-1-ylsulfinyl)methyl)benzene (**2j**), 1-((6-chlorohexyl)sulfinyl)hex-1-yne (**2k**), and 3-butyl-2-(ethylsulfinyl)benzofuran (**7**) were prepared from the corresponding sulfides.

*Synthesis of 4-(ethylsulfinyl)but-3-yn-1-yl 4-methylbenzoate (**2c**)*[S9]

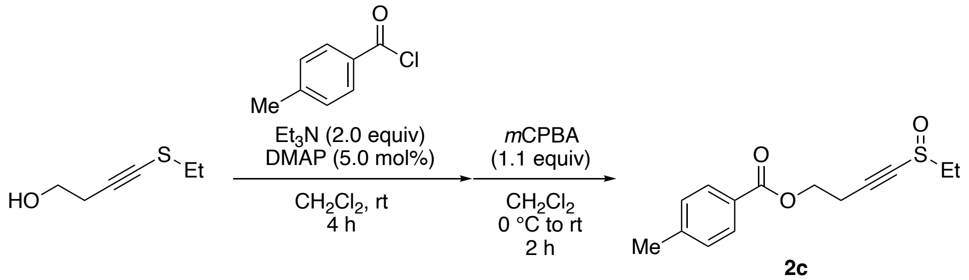

2c

To a mixture of 4-(ethylthio)but-3-yn-1-ol (49.5 mg, 0.380 mmol), Et_3N (106 µL, 0.760 mmol, 2.0 equiv), and 4-dimethylaminopyridine (DMAP) (2.3 mg, 19 µmol, 5 mol%) dissolved in CH_2Cl_2 (3 mL) was added 4-methylbenzoyl chloride (75.3 µL, 0.570 mmol, 1.5 equiv) at 0 °C. After stirring for 10 min at the same temperature, the mixture was allowed to warm to room temperature. After stirring for 4 h at room temperature, to the mixture was added water (10 mL). The mixture was extracted with CH_2Cl_2 (15 mL × 3). The combined organic extract was washed with brine (20 mL) and dried (Na_2SO_4). After filtration, the filtrate was concentrated under reduced pressure. The residue was purified by flash column chromatography (silica-gel 4 g, *n*-hexane/EtOAc = 20/1) to give 4-(ethylthio)but-3-yn-1-yl 4-methylbenzoate (78.7 mg, 0.317 mmol, 84%) as a colorless oil.

To a solution of 4-(ethylthio)but-3-yn-1-yl 4-methylbenzoate (78.7 mg, 0.317 mmol) in CH_2Cl_2 (2 mL) was slowly added *m*CPBA (ca. 77%, 76.2 mg, ca. 0.34 mmol, ca. 1.1 equiv) at 0 °C. After stirring for 10 min at the same temperature, the mixture was allowed to warm to room temperature. After stirring for 2 h at room temperature, to the mixture was added an aqueous saturated solution of potassium carbonate (5 mL) and an aqueous saturated solution of sodium thiosulfate (5 mL). The mixture was extracted with CH_2Cl_2 (10 mL × 3). The combined organic extract was washed with brine (10 mL) and dried (Na_2SO_4). After filtration, the filtrate was concentrated under reduced pressure. The residue was purified by flash column chromatography (silica-gel 2.4 g, AcOEt) to give 4-(ethylsulfinyl)but-3-yn-1-yl 4-methylbenzoate (**2c**) (73.0 mg, 0.276 mmol, 88%) as a colorless solid.

*Synthesis of (2-((3-(ethylsulfinyl)prop-2-yn-1-yl)oxy)ethyl)benzene (**2d**)*

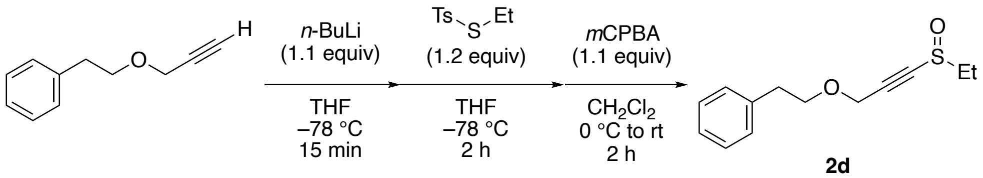

2d

To a solution of (2-(prop-2-yn-1-yloxy)ethyl)benzene (161 mg, 1.00 mmol) in THF (2.5 mL) was added *n*-BuLi (1.57 M in *n*-hexane, 0.70 mL, 1.1 mmol, 1.1 equiv) at 0 °C. After stirring for 15 min at the same temperature, to the mixture was added *S*-ethyl 4-methylbenzenesulfonothioate (260 mg, 1.20 mmol, 1.2 equiv) dissolved in THF (2.5 mL). After stirring for 2 h at the same temperature, the mixture was allowed to warm to room temperature. After stirring for 15 min at room temperature, to the mixture was added an aqueous saturated solution of ammonium chloride (5 mL). The mixture was extracted with EtOAc (10 mL × 3). The combined organic extract was washed with brine (20 mL) and dried (Na_2SO_4). After filtration, the filtrate was concentrated under reduced pressure. The residue was purified by flash column chromatography (silica-gel 8 g, *n*-hexane/EtOAc = 10/1) to give ethyl 3-phenethoxyprop-1-yn-1-yl sulfide (185 mg, 0.838 mmol, 84%) as a yellow oil.

To a solution of ethyl(3-phenethoxyprop-1-yn-1-yl)sulfane (184.7 mg, 0.838 mmol) in CH_2Cl_2 (4 mL) was slowly added *m*CPBA (ca. 77%, 187.8 mg, ca. 0.84 mmol, ca. 1.1 equiv) at 0 °C. After stirring for 10 min at the same temperature, the mixture was allowed to warm to room temperature. After stirring for 2 h at room temperature, to the mixture was added an aqueous saturated solution of potassium carbonate (5 mL) and an aqueous saturated solution of sodium thiosulfate (5 mL). The mixture was extracted with CH_2Cl_2 (10 mL × 3). The combined organic extract was washed with brine (10 mL) and dried (Na_2SO_4). After filtration, the filtrate was concentrated under reduced pressure. The residue was purified by flash column chromatography (silica-gel 2 g, CH_2Cl_2) to give (2-((3-(ethylsulfinyl)prop-2-yn-1-yl)oxy)ethyl)benzene (**2d**) (128 mg, 0.539 mmol, 64%) as a colorless oil.

*Synthesis of 3-butyl-2-(ethylsulfonyl)benzofuran (**10**)*

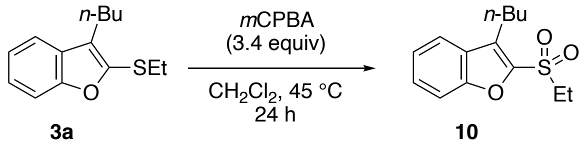

In a 5 mL screw-top V-vial® with a solid-top cap (Sigma-Aldrich, Cat. No. Z115118), to a solution of 3-butyl-2-(ethylthio)benzofuran (**3a**) (23.2 mg, 99.1 µmol) dissolved in dichloromethane (1.5 mL) was added *m*-chloroperoxybenzoic acid (ca. 77%) (67.2 mg, ca. 0.34 mmol, ca. 3.4 equiv) at room temperature. After stirring for 24 h at 45 °C, to the mixture was added saturated aqueous potassium carbonate (5 mL) and saturated aqueous sodium thiosulfate (5 mL). The mixture was extracted with CH_2Cl_2 (10 mL × 3). The combined organic extract was washed with brine (10 mL) and dried with Na_2SO_4. After filtration, the filtrate was concentrated under reduced pressure. The residue was purified preparative TLC (*n*-hexane/EtOAc = 1/1) to give 3-butyl-2-(ethylsulfonyl)benzo[*b*]furan (**10**) (16.0 mg, 60.2 µmol, 61%) as a colorless solid.

*Synthesis of 3-butyl-2-(2-(ethylthio)-3-methoxyphenoxy)benzofuran (**12**)*

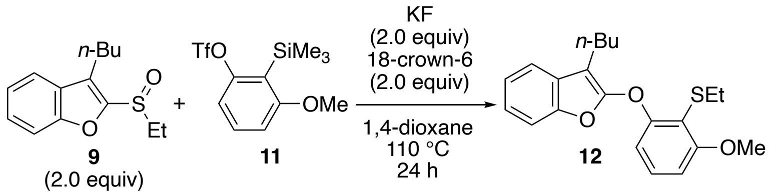

To a mixture of 3-methoxy-2-(trimethylsilyl)phenyl triflate (**11**) (16.8 mg, 51.2 µmol) and 3-butyl-2-(ethylsulfinyl)benzofuran (**9**) (25.0 mg, 0.100 mmol, 2.0 equiv) dissolved in 1,4-dioxane (1.0 mL) were added 18-crown-6-ether (26.4 mg, 0.100 mmol, 2.0 equiv) and potassium fluoride (5.8 mg, 0.10 mmol, 2.0 equiv) at room temperature. After stirring for 24 h at 110 °C, the mixture was cooled to room temperature. To this was added water (3 mL). The mixture was extracted with EtOAc (10 mL × 3). The combined organic extract was washed with brine (5 mL) and dried (Na_2SO_4). After filtration, the filtrate was concentrated under reduced pressure. The residue was purified by preparative TLC (*n*-hexane/CH_2Cl_2 = 1/1) to give 3-butyl-2-(2-(ethylthio)-3-methoxyphenoxy)benzo[*b*]furan (**12**) (7.1 mg, 20 µmol, 39%) as a colorless oil.

Mechanistic Studies

*Control experiments through the reaction of **2a** with TFAA.*

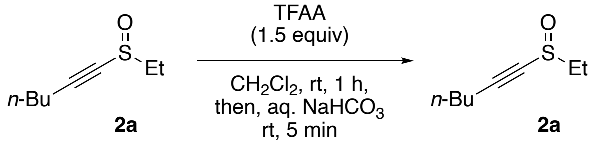

To a solution of 1-(ethylsulfinyl)hex-1-yne (**2a**) (7.7 mg, 49 μmol) in dichloromethane (0.50 mL) was added trifluoroacetic anhydride (10.5 μL, 75.5 μmol, 1.5 equiv) at room temperature. After stirring for 1 h at room temperature, to the mixture was added an aqueous saturated solution of sodium bicarbonate (5 mL). After stirring for 5 min at room temperature, the mixture was extracted with dichloromethane (15 mL × 3). The combined organic extract was washed with brine (20 mL) and dried (Na_2SO_4). After filtration, the filtrate was concentrated under reduced pressure. To the residue was added 1,1,2,2-tetrachloroethane (18.4 mg, 0.110 mmol) as an internal standard. The mixture was dissolved in $CDCl_3$ and analyzed by ^{1}H NMR (400 MHz). As a result, ^{1}H NMR yield of **2a** was determined to be 57% by comparing the relative values of integration for the peaks observed at 2.44 ppm (2H for **2a**) with that of 1,1,2,2-tetrachloroethane observed at 5.96 ppm.

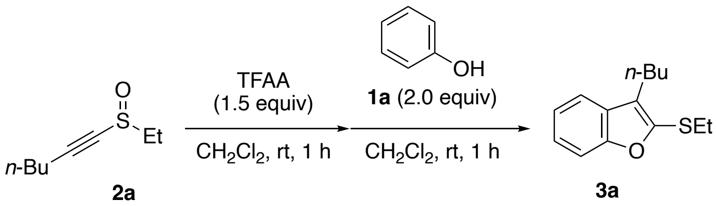

To a solution of 1-(ethylsulfinyl)hex-1-yne (**2a**) (8.1 mg, 51 μmol) in dichloromethane (0.50 mL) was added trifluoroacetic anhydride (10.5 μL, 75.5 μmol, 1.5 equiv) at room temperature. After stirring for 1 h at room temperature, to the mixture was added phenol (**1a**) (9.4 mg, 0.10 mmol, 2.0 equiv) in dichloromethane (0.50 mL). After stirring for 1 h at the same temperature, the mixture was extracted with dichloromethane (15 mL × 3). The combined organic extract was washed with brine (20 mL) and dried (Na_2SO_4). After filtration, the filtrate was concentrated under reduced pressure. To the residue was added 1,1,2,2-tetrachloroethane (18.1 mg, 0.108 mmol) as an internal standard. The mixture was dissolved in $CDCl_3$ and analyzed by ^{1}H NMR (400 MHz). As a result, ^{1}H NMR yield of **3a** was determined to be 55% by comparing the relative values of integration for the peaks observed at 7.42 ppm (1H for **3a**) with that of 1,1,2,2-tetrachloroethane observed at 5.96 ppm.

Computational Methods

The optimized structures of methyl propyl sulfoxide, methyl propenyl sulfoxide, methyl propynyl sulfoxides, TFAA, and sulfuranes **Ia**–**Ic** were computed with Spartan '18 program (Wavefunction. Inc., Irvine, CA) using density functional theory (B3LYP/6-311G(d,p)).

Methyl propyl sulfoxide

Optimized structure (C: black; H: grey; O: red; S: green)

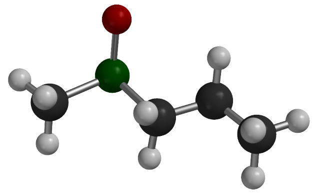

(total energy: –631.918362 hartrees)

Cartesian Coordinates (Angstroms)

H	1.011881	1.705835	-3.145414
C	0.886580	1.634150	-2.065314
H	1.860248	1.531298	-1.583848
H	0.348146	2.507910	-1.693206
S	-0.102806	0.120685	-1.753431
O	0.762521	-1.060247	-2.151177
C	-0.054835	0.213521	0.092662
H	0.999681	0.285562	0.375360
H	-0.571307	1.136250	0.376971
C	-0.712143	-1.019850	0.707724
H	-0.221301	-1.911960	0.309033
H	-1.761201	-1.068970	0.395435
C	-0.627887	-1.016596	2.237993
H	0.412463	-0.999725	2.576097
H	-1.096848	-1.911977	2.653861
H	-1.133190	-0.145886	2.667255

Trifluoroacetic anhydride

Optimized structure (C: black; H: grey; O: red; S: green; F: blue)

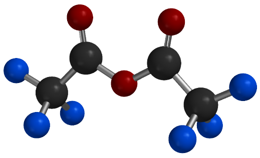

(total energy: –977.443916 hartrees)

Cartesian Coordinates (Angstroms)

C	-2.325197	0.000000	0.397337
C	-1.213869	0.000000	-0.691054
O	-1.411005	0.000000	-1.855500
O	0.000000	0.000000	-0.034694
C	1.213869	0.000000	-0.691054
O	1.411005	0.000000	-1.855500
C	2.325197	0.000000	0.397337
F	-2.216261	1.089317	1.172969
F	-2.216261	-1.089317	1.172969
F	-3.522758	0.000000	-0.179374
F	2.216261	1.089317	1.172969
F	2.216261	-1.089317	1.172969
F	3.522758	0.000000	-0.179374

Sulfurane Ia

Optimized structure (C: black; H: grey; O: red; S: green; F: blue)

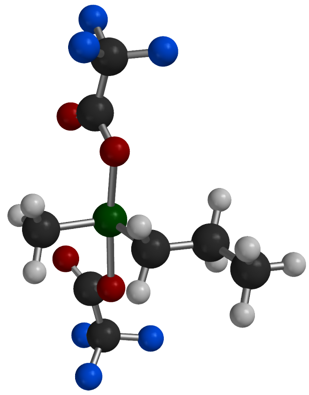

(total energy: –1609.395718 hartrees)

Cartesian Coordinates (Angstroms)

S	-0.065968	-0.080285	0.569860
C	-1.636982	-0.612696	1.336838
H	-2.398458	0.119175	1.080234
H	-1.455965	-0.628575	2.409499
H	-1.874929	-1.602325	0.957189
C	-0.481310	0.021100	-1.226619
H	-0.841983	-0.970073	-1.496824
H	-1.281749	0.755388	-1.295765
O	-0.622316	1.806931	0.664776
C	-0.459287	2.367793	1.836388
O	-0.221792	1.830999	2.888987
C	-0.621433	3.914394	1.727265
F	0.299766	4.426882	0.886424
F	-1.840860	4.235495	1.247812
F	-0.475304	4.502508	2.914421
O	0.435444	-1.944922	0.201417
C	0.983911	-2.570650	1.213152
O	1.053249	-2.207985	2.359853
C	1.558857	-3.946707	0.757597
F	0.559865	-4.759797	0.352600
F	2.206741	-4.551032	1.753589
F	2.413260	-3.799542	-0.273410
C	0.746628	0.428014	-2.037366
H	1.541163	-0.307450	-1.886232
H	1.116270	1.393153	-1.679794
C	0.402468	0.524886	-3.528341
H	1.281538	0.826935	-4.101568
H	-0.382483	1.264662	-3.708818
H	0.061661	-0.436275	-3.923163

Methyl propenyl sulfoxide

Optimized structure (C: black; H: grey; O: red; S: green)

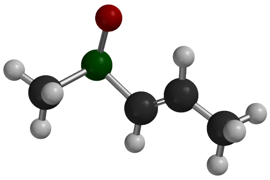

(total energy: –630.685318 hartrees)

Cartesian Coordinates (Angstroms)

H	1.196986	1.505697	-2.939815
C	0.975714	1.390063	-1.878786
H	1.827937	0.959045	-1.353280

H	0.687020	2.350747	-1.448164
S	-0.440452	0.221907	-1.756640
O	0.076259	-1.147388	-2.149109
C	-0.576166	0.252854	0.048148
H	-0.878979	1.208889	0.466328
C	-0.404191	-0.865280	0.744358
H	-0.128061	-1.757632	0.184109
C	-0.556695	-0.992664	2.229221
H	0.375645	-1.346404	2.681856
H	-1.324696	-1.734458	2.473348
H	-0.830320	-0.045376	2.698427

Sulfurane Ib

Optimized structure (C: black; H: grey; O: red; S: green; F: blue)

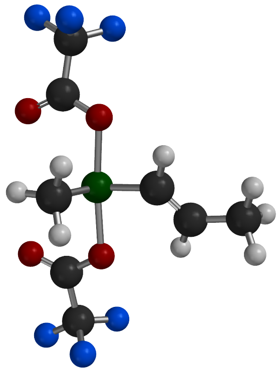

(total energy: –1608.156494 hartrees)

Cartesian Coordinates (Angstroms)

S	0.164881	-0.164519	0.398667
C	-1.459328	-0.689465	1.067452
H	-2.170991	0.103960	0.858420
H	-1.307480	-0.824493	2.136867
H	-1.732362	-1.622507	0.581316
O	-0.377296	1.727173	0.499841
C	-0.254895	2.262145	1.689053
O	-0.054744	1.707278	2.739377
C	-0.427400	3.809774	1.602152
F	0.452823	4.340496	0.728092
F	-1.669158	4.123308	1.176325
F	-0.237135	4.388258	2.787225
O	0.644626	-2.047148	0.095428
C	1.086384	-2.670201	1.159463
O	1.089631	-2.280796	2.299577
C	1.615698	-4.088203	0.783774
F	0.595130	-4.864871	0.359203
F	2.178666	-4.683166	1.836186
F	2.527603	-4.029797	-0.204120
C	-0.105355	-0.125963	-1.383854
H	-0.298680	-1.117683	-1.769372
C	0.033998	0.963734	-2.131644
H	0.250028	1.916118	-1.662135
C	-0.111944	0.953600	-3.621030
H	0.813447	1.305156	-4.088273
H	-0.902089	1.646521	-3.927368
H	-0.344058	-0.038707	-4.010624

Methyl propynyl sulfoxide

Optimized structure (C: black; H: grey; O: red; S: green)

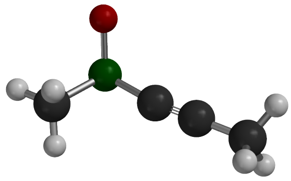
(total energy: –629.423397 hartrees)

Cartesian Coordinates (Angstroms)

H	0.960073	1.674712	-2.868487
C	0.818856	1.382491	-1.827764
H	1.755751	1.030876	-1.397738
H	0.394913	2.204271	-1.249675
S	-0.374522	-0.017638	-1.852861
O	0.327702	-1.172568	-2.518138
C	-0.409416	-0.258450	-0.122140
C	-0.546799	-0.596693	1.030031
C	-0.700807	-0.980802	2.423556
H	0.049148	-0.493979	3.052882
H	-0.582742	-2.062627	2.533181
H	-1.692158	-0.709593	2.797153

Sulfurane Ic

Optimized structure (C: black; H: grey; O: red; S: green; F: blue)

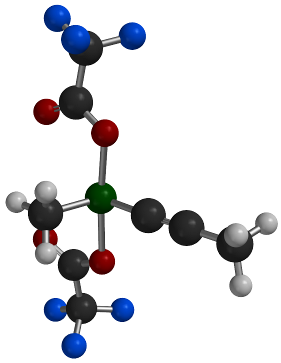
(total energy: –1606.890141 hartrees)

Cartesian Coordinates (Angstroms)

S	-0.024231	0.241804	0.263634
C	0.566594	-1.271762	1.127652
H	-0.180808	-2.043286	0.970816
H	0.646921	-0.985457	2.174165
H	1.529227	-1.530666	0.697559
O	-1.903677	-0.364992	0.452806
C	-2.396693	-0.116454	1.638064
O	-1.805730	0.263941	2.618616
C	-3.929583	-0.401553	1.668602
F	-4.576786	0.336209	0.747991
F	-4.168770	-1.702476	1.401658
F	-4.445592	-0.122156	2.866201
O	1.848694	0.753463	-0.133456
C	2.425476	1.359281	0.872050
O	2.009611	1.478725	1.998301
C	3.812235	1.934065	0.449354
F	4.636469	0.937161	0.065174
F	4.389939	2.581790	1.462119
F	3.687175	2.789024	-0.582313
C	-0.136151	-0.239174	-1.356738
C	-0.258679	-0.485305	-2.528764
C	-0.402507	-0.792924	-3.938818

H	-0.432419	0.126827	-4.529094
H	-1.328052	-1.347085	-4.116923
H	0.437334	-1.398998	-4.288656

Characterization Data of New Compounds

Ethyl hex-1-yn-1-yl sulfide,[S11] hex-1-yn-1-yl methyl sulfide,[S11] benzyl hex-1-yn-1-yl sulfide,[S11] 1-(ethylsulfinyl)hex-1-yne (**2a**),[S12] and 1-(methylsulfinyl)hex-1-yne (**2i**)[S13] were identical in spectra data with those reported in the literature.

3-Butyl-2-(ethylthio)benzo[*b*]furan (**3a**)

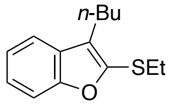

Colorless oil; TLC R_f 0.55 (*n*-hexane/CH_2Cl_2 = 10/1); ^{1}H NMR ($CDCl_3$, 400 MHz): δ 0.95 (t, 3H, *J* = 7.4 Hz), 1.29 (t, 3H, *J* = 7.4 Hz), 1.33–1.46 (m, 2H), 1.58–1.71 (m, 2H), 2.76 (t, 2H, *J* = 7.6 Hz), 2.89 (q, 2H, *J* = 7.4 Hz), 7.21 (ddd, 1H, *J* = 7.8, 7.8, 1.0 Hz), 7.28 (ddd, 1H, *J* = 7.8, 7.8 1.0 Hz), 7.42 (dd, 1H, *J* = 7.8, 1.0 Hz), 7.51 (dd, 1H, *J* = 7.8, 1.0 Hz); $^{13}C\{^1H\}$ NMR ($CDCl_3$, 101 MHz): δ 13.9, 15.3, 22.5, 24.3, 29.4, 31.8, 111.0, 119.5, 122.4, 124.5, 125.6, 129.0, 145.7, 155.6; HRMS (EI) *m/z*: $[M]^{\cdot+}$ Calcd for $C_{14}H_{18}OS^{\cdot+}$ 234.1078; Found 234.1080.

2-(Ethylthio)-3-phenethylbenzo[*b*]furan (**3b**)

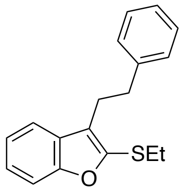

Colorless oil; TLC R_f 0.35 (*n*-hexane/CH_2Cl_2 = 10/1); ^{1}H NMR ($CDCl_3$, 400 MHz): δ 1.22 (t, 3H, *J* = 7.4 Hz), 2.75 (q, 2H, *J* = 7.4 Hz), 2.93–3.01 (m, 2H), 3.03–3.11 (m, 2H), 7.13–7.33 (m, 7H), 7.43 (d, 1H, *J* = 8.1 Hz), 7.48 (d, 1H, *J* = 7.6 Hz); $^{13}C\{^1H\}$ NMR ($CDCl_3$, 101 MHz): δ 15.3, 26.8, 29.3, 35.9, 111.1, 119.4, 122.4, 124.5, 124.6, 126.0, 128.4, 128.5, 128.7, 141.4, 146.4, 155.6; HRMS (EI) *m/z*: $[M]^{\cdot+}$ Calcd for $C_{18}H_{18}OS^{\cdot+}$ 282.1078; Found 282.1077.

2-(2-(Ethylthio)benzo[*b*]furan-3-yl)ethyl 4-methylbenzoate (**3c**)

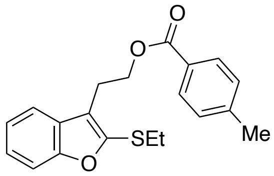

Colorless solid; TLC R_f 0.57 (*n*-hexane/CH_2Cl_2 = 1/1); ^{1}H NMR ($CDCl_3$, 400 MHz): δ 1.29 (t, 3H, *J* = 7.4 Hz), 2.40 (s, 3H), 2.92 (q, 2H, *J* = 7.4 Hz), 3.23 (t, 2H, *J* = 7.0 Hz), 4.54 (t, 2H, *J* = 7.0 Hz), 7.18–7.35 (m, 4H), 7.44 (d, 1H, *J* = 8.1 Hz), 7.56 (d, 1H, *J* = 7.7 Hz), 7.85–7.93 (AA'BB', 2H); $^{13}C\{^1H\}$ NMR ($CDCl_3$, 101 MHz): δ 15.3, 21.6, 24.4, 29.3, 63.8, 111.0, 119.4, 121.1, 122.6, 124.8, 127.3, 128.6, 129.0, 129.7, 143.6, 147.3, 155.6, 166.6; HRMS (ESI) *m/z*: $[M + Na]^+$ Calcd for $C_{20}H_{20}NaO_3S^+$ 363.1031; Found 363.1032.

2-(Ethylthio)-3-(phenethoxymethyl)benzo[*b*]furan (**3d**)

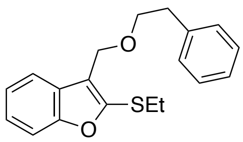

Colorless oil; TLC R_f 0.28 (*n*-hexane/CH_2Cl_2 = 3/1); ^{1}H NMR ($CDCl_3$, 400 MHz): δ 1.28 (t, 3H, *J* = 7.4 Hz), 2.87–2.98 (m, 4H), 3.71 (t, 2H, *J* = 7.1 Hz), 4.72 (s, 2H), 7.16–7.24 (m, 4H), 7.24–7.33 (m, 3H), 7.42 (d, 1H, *J* = 8.2 Hz), 7.53 (d, 1H, *J* = 7.7 Hz); $^{13}C\{^1H\}$ NMR ($CDCl_3$, 101 MHz): δ 15.3, 29.3, 36.2, 63.8, 70.8, 110.8, 120.3, 121.6, 122.8, 124.8, 126.1, 128.2, 128.3, 128.9, 138.9, 148.2, 155.7; HRMS (ESI) *m/z*: $[M + Na]^+$ Calcd for $C_{19}H_{20}NaO_2S^+$ 335.1082; Found 335.1078.

2-(Ethylthio)-3-(4-tolyl)benzo[*b*]furan (**3e**)

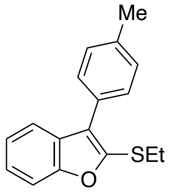

Colorless oil; TLC R_f 0.43 (*n*-hexane/CH_2Cl_2 = 10/1); ^{1}H NMR ($CDCl_3$, 400 MHz): δ 1.29 (t, 3H, *J* = 7.4 Hz), 2.44 (s, 3H), 2.98 (q, 2H, *J* = 7.4 Hz), 7.26 (dd, 1H, *J* = 7.8, 7.8 Hz), 7.29–7.37 (m, 3H), 7.46–7.56 (m, 3H), 7.63 (d, 1H, *J* = 7.8 Hz); ^{13}C{^{1}H} NMR ($CDCl_3$, 101 MHz): δ 15.2, 21.3, 28.8, 110.9, 119.9, 122.9, 124.4, 124.6, 128.4, 128.8, 129.1, 129.2, 137.3, 146.3, 155.5; HRMS (ESI) *m/z*: [M + K]$^+$ Calcd for $C_{17}H_{16}KOS^+$ 307.0559; Found 307.0559.

3-(4-Chlorophenyl)-2-(ethylthio)benzo[*b*]furan (**3f**)

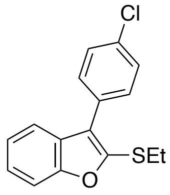

Pale yellow oil; TLC R_f 0.51 (*n*-hexane/CH_2Cl_2 = 10/1); ^{1}H NMR ($CDCl_3$, 400 MHz): δ 1.28 (t, 3H, *J* = 7.4 Hz), 2.99 (q, 2H, *J* = 7.4 Hz), 7.27 (ddd, 1H, *J* = 7.7, 7.7, 1.0 Hz), 7.34 (ddd, 1H, *J* = 7.7, 7.7, 1.3 Hz), 7.44–7.53 (m, 3H), 7.53–7.61 (m, 3H); ^{13}C{^{1}H} NMR ($CDCl_3$, 101 MHz): δ 15.3, 28.8, 111.1, 119.6, 123.1, 123.2, 124.8, 128.0, 128.8, 130.4, 130.5, 133.4, 146.9, 155.6; HRMS (ESI) *m/z*: [M + K]$^+$ Calcd for $C_{16}H_{13}{}^{35}ClKOS^+$ 327.0013; Found 327.0012.

3-Butyl-2-(4-tolylthio)benzo[*b*]furan (**3g**)

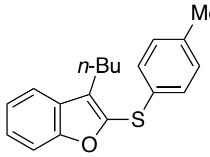

Colorless oil; TLC R_f 0.71 (*n*-hexane/CH_2Cl_2 = 3/1); ^{1}H NMR ($CDCl_3$, 400 MHz): δ 0.94 (t, 3H, *J* = 7.4 Hz), 1.33–1.48 (m, 2H), 1.62–1.74 (m, 2H), 2.31 (s, 3H), 2.86 (t, 2H, *J* = 7.9 Hz), 7.04–7.12 (AA'BB', 2H), 7.14–7.22 (AA'BB', 2H), 7.26 (dd, 1H, *J* = 7.7, 7.7 Hz), 7.33 (dd, 1H, *J* = 7.7, 7.7 Hz), 7.46 (d, 1H, *J* = 7.7 Hz), 7.59 (d, 1H, *J* = 7.7 Hz); ^{13}C{^{1}H} NMR ($CDCl_3$, 101 MHz): δ 13.9, 21.0, 22.5, 24.3, 31.8, 111.4, 119.9, 122.4, 125.2, 127.8, 128.4, 128.7, 129.8, 131.5, 136.5, 143.7, 155.9; HRMS (ESI) *m/z*: [M + Na]$^+$ Calcd for $C_{19}H_{20}NaOS^+$ 319.1133; Found 319.1133.

2-((4-Bromophenyl)thio)-3-butylbenzo[*b*]furan (**3h**)

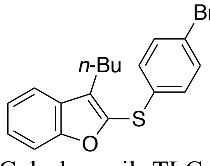

Colorless oil; TLC R_f 0.74 (*n*-hexane/CH_2Cl_2 = 3/1); ^{1}H NMR ($CDCl_3$, 400 MHz): δ 0.93 (t, 3H, *J* = 7.4 Hz), 1.30–1.45 (m, 2H), 1.60–1.72 (m, 2H), 2.83 (t, 2H, *J* = 7.6 Hz), 7.03–7.12 (AA'BB', 2H), 7.27 (dd, 1H, *J* = 7.8, 7.8 Hz), 7.32–7.41 (m, 3H), 7.46 (d, 1H, *J* = 7.8 Hz), 7.60 (d, 1H, *J* = 7.8 Hz); ^{13}C{^{1}H} NMR ($CDCl_3$, 101 MHz): δ 13.8, 22.5, 24.3, 31.7, 111.5, 120.1, 120.3, 122.6, 125.6, 128.5, 128.9, 129.2, 132.1, 134.7, 142.1, 156.0; HRMS (EI) *m/z*: [M]$^{\cdot+}$ Calcd for $C_{18}H_{17}{}^{79}BrOS^{\cdot+}$ 360.0183; Found 360.0182.

3-Butyl-2-(ethylthio)-5-methylbenzo[*b*]furan (**3i**)

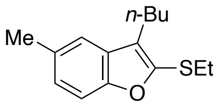

Colorless oil; TLC R_f 0.50 (*n*-hexane/CH_2Cl_2 = 10/1); ^1H NMR ($CDCl_3$, 400 MHz): δ 0.95 (t, 3H, *J* = 7.4 Hz), 1.28 (t, 3H, *J* = 7.4 Hz), 1.33–1.45 (m, 2H), 1.58–1.70 (m, 2H), 2.45 (s, 3H), 2.75 (t, 2H, *J* = 7.7 Hz), 2.88 (q, 2H, *J* = 7.4 Hz), 7.09 (d, 1H, *J* = 8.4 Hz), 7.26–7.33 (m, 2H); $^{13}C\{^1H\}$ NMR ($CDCl_3$, 101 MHz): δ 13.9, 15.3, 21.4, 22.5, 24.3, 29.4, 31.8, 110.5, 119.3, 125.4, 125.7, 129.0, 131.7, 145.7, 154.0; HRMS (EI) *m/z*: $[M]^{\cdot+}$ Calcd for $C_{15}H_{20}OS^{\cdot+}$ 248.1235; Found 248.1234.

3-Butyl-2-(ethylthio)-5-methoxybenzo[*b*]furan (**3j**)

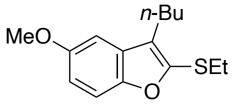

Pale yellow oil; TLC R_f 0.36 (*n*-hexane/CH_2Cl_2 = 10/1); ^1H NMR ($CDCl_3$, 400 MHz): δ 0.95 (t, 3H, *J* = 7.4 Hz), 1.28 (t, 3H, *J* = 7.4 Hz), 1.32–1.45 (m, 2H), 1.57–1.69 (m, 2H), 2.72 (t, 2H, *J* = 7.7 Hz), 2.88 (q, 2H, *J* = 7.4 Hz), 3.86 (s, 3H), 6.88 (dd, 1H, *J* = 8.9, 2.6 Hz), 6.93 (d, 1H, *J* = 2.6 Hz), 7.30 (d, 1H, *J* = 8.9 Hz); $^{13}C\{^1H\}$ NMR ($CDCl_3$, 101 MHz): δ 13.9, 15.3, 22.5, 24.3, 29.4, 31.7, 56.0, 102.2, 111.4, 112.9, 125.5, 129.4, 146.5, 150.6, 155.6; HRMS (EI) *m/z*: $[M]^{\cdot+}$ Calcd for $C_{15}H_{20}O_2S^{\cdot+}$ 264.1184; Found 264.1185.

5-Bromo-3-butyl-2-(ethylthio)benzo[*b*]furan (**3k**)

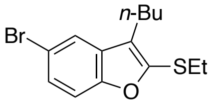

Colorless oil; TLC R_f 0.58 (*n*-hexane/CH_2Cl_2 = 10/1); ^1H NMR ($CDCl_3$, 400 MHz): δ 0.95 (t, 3H, *J* = 7.4 Hz), 1.28 (t, 3H, *J* = 7.4 Hz), 1.32–1.43 (m, 2H), 1.55–1.67 (m, 2H), 2.70 (t, 2H, *J* = 7.7 Hz), 2.90 (q, 2H, *J* = 7.4 Hz), 7.28 (d, 1H, *J* = 8.6 Hz), 7.35 (dd, 1H, *J* = 8.6, 2.0 Hz), 7.61 (d, 1H, *J* = 2.0 Hz); $^{13}C\{^1H\}$ NMR ($CDCl_3$, 101 MHz): δ 13.9, 15.3, 22.5, 24.1, 29.2, 31.7, 112.4, 115.4, 122.1, 124.7, 127.2, 131.0, 147.5, 154.3; HRMS (EI) *m/z*: $[M]^{\cdot+}$ Calcd for $C_{14}H_{17}{}^{79}BrOS^{\cdot+}$ 312.0183; Found 312.0186.

3-Butyl-5-chloro-2-(ethylthio)benzo[*b*]furan (**3l**)

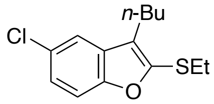

Colorless oil; TLC R_f 0.56 (*n*-hexane/CH_2Cl_2 = 10/1); ^1H NMR ($CDCl_3$, 400 MHz): δ 0.95 (t, 3H, *J* = 7.4 Hz), 1.28 (t, 3H, *J* = 7.4 Hz), 1.32–1.44 (m, 2H), 1.57–1.68 (m, 2H), 2.70 (t, 2H, *J* = 7.7 Hz), 2.91 (q, 2H, *J* = 7.4 Hz), 7.22 (dd, 1H, *J* = 8.7, 2.1 Hz), 7.32 (d, 1H, *J* = 8.7 Hz), 7.45 (d, 1H, *J* = 2.1 Hz); $^{13}C\{^1H\}$ NMR ($CDCl_3$, 101 MHz): δ 13.9, 15.3, 22.5, 24.2, 29.2, 31.7, 111.9, 119.1, 124.5, 124.9, 127.9, 130.3, 147.6, 153.9; HRMS (ESI) *m/z*: $[M + K]^+$ Calcd for $C_{14}H_{17}{}^{35}ClKOS^+$ 307.0326; Found 307.0325.

Methyl 3-butyl-2-(ethylthio)benzo[*b*]furan-5-carboxylate (**3m**)

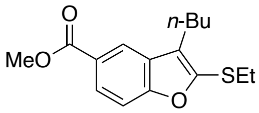

Colorless oil; TLC R_f 0.46 (*n*-hexane/EtOAc = 10/1); ^1H NMR ($CDCl_3$, 400 MHz): δ 0.95 (t, 3H, *J* = 7.4 Hz), 1.29 (t, 3H, *J* = 7.4 Hz), 1.33–1.45 (m, 2H), 1.56–1.71 (m, 2H), 2.77 (t, 2H, *J* = 7.7 Hz), 2.92 (q, 2H, *J* = 7.4 Hz), 3.95 (s, 3H), 7.43 (d, 1H, *J* = 8.6 Hz), 8.00 (dd, 1H, *J* = 8.6, 1.7 Hz), 8.22 (d, 1H, *J* = 1.7 Hz); $^{13}C\{^1H\}$ NMR ($CDCl_3$, 101 MHz): δ 13.9, 15.3, 22.5, 24.2, 29.2, 31.8, 52.1, 110.8, 121.9, 124.6, 125.8, 126.2, 129.0, 147.6, 158.2, 167.3; HRMS (ESI) *m/z*: $[M + Na]^+$ Calcd for $C_{16}H_{20}NaO_3S^+$ 315.1031; Found 315.1036.

2-(3-Butyl-2-(ethylthio)benzo[*b*]furan-5-yl)-4,4,5,5-tetramethyl-1,3,2-dioxaborolane (**3n**)

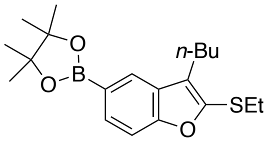

Colorless oil; TLC R_f 0.42 (*n*-hexane/CH_2Cl_2 = 1/1); ^{1}H NMR ($CDCl_3$, 400 MHz): δ 0.95 (t, 3H, *J* = 7.4 Hz), 1.27 (t, 3H, *J* = 7.4 Hz), 1.32–1.45 (m, 14H), 1.60–1.72 (m, 2H), 2.76 (t, 2H, *J* = 7.7 Hz), 2.89 (q, 2H, *J* = 7.4 Hz), 7.40 (d, 1H, *J* = 8.2 Hz), 7.74 (d, 1H, *J* = 8.2 Hz), 7.97 (s, 1H); ^{13}C{^{1}H} NMR ($CDCl_3$, 101 MHz): δ 13.9, 15.3, 22.6, 24.3, 24.9, 29.3, 32.0, 83.8, 110.4, 125.8, 126.7, 128.7, 131.2, 145.8, 157.8 (the signal for the carbon which is attached to the boron atom was not observed); HRMS (ESI) *m/z*: [M + Na]$^+$ Calcd for $C_{20}H_{29}BNaO_3S^+$ 383.1828; Found 383.1832.

3-Butyl-2-(ethylthio)-7-(trimethylsilyl)benzo[*b*]furan-6-yl trifluoromethanesulfonate (**3o**)

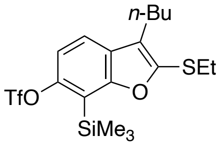

Colorless oil; TLC R_f 0.37 (*n*-hexane/CH_2Cl_2 = 10/1); ^{1}H NMR ($CDCl_3$, 400 MHz): δ 0.51 (s, 9H), 0.94 (t, 3H, *J* = 7.3 Hz), 1.30 (t, 3H, *J* = 7.3 Hz), 1.32–1.43 (m, 2H), 1.55–1.66 (m, 2H), 2.71 (t, 2H, *J* = 7.6 Hz), 2.92 (q, 2H, *J* = 7.3 Hz), 7.19 (d, 1H, *J* = 8.6 Hz), 7.49 (d, 1H, *J* = 8.6 Hz); ^{13}C{^{1}H} NMR ($CDCl_3$, 101 MHz): δ 0.3, 13.9, 15.4, 22.5, 24.0, 29.0, 31.8, 115.2, 115.9, 118.6 (q, $J_{C–F}$ = 322 Hz) 120.9, 123.9, 127.6, 147.9, 151.2, 159.6; ^{19}F NMR ($CDCl_3$, 376 MHz): δ –73.1 (s); HRMS (ESI) *m/z*: [M + K]$^+$ Calcd for $C_{18}H_{25}F_3KO_4S_2Si^+$ 493.0553; Found 493.0553.

3-Butyl-2-(ethylthio)naphtho[1,2-*b*]furan (**3p**)

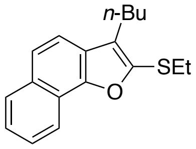

Colorless oil; TLC R_f 0.39 (*n*-hexane/CH_2Cl_2 = 10/1); ^{1}H NMR ($CDCl_3$, 400 MHz): δ 0.97 (t, 3H, *J* = 7.4 Hz), 1.33 (t, 3H, *J* = 7.4 Hz), 1.37–1.49 (m, 2H), 1.65–1.77 (m, 2H), 2.85 (t, 2H, *J* = 7.4 Hz), 2.93 (q, 2H, *J* = 7.4 Hz), 7.49 (dd, 1H, *J* = 8.1, 8.1 Hz), 7.54–7.62 (m, 2H), 7.65 (d, 1H, *J* = 8.6 Hz), 7.92 (d, 1H, *J* = 8.1 Hz), 8.33 (d, 1H, *J* = 8.1 Hz); ^{13}C{^{1}H} NMR ($CDCl_3$, 101 MHz): δ 13.9, 15.3, 22.5, 24.2, 30.1, 32.2, 118.2, 120.3, 121.0, 122.9, 124.3, 125.3, 126.3, 127.4, 128.3, 131.5, 144.7, 151.5; HRMS (EI) *m/z*: $[M]^{\cdot+}$ Calcd for $C_{18}H_{20}OS^{\cdot+}$ 284.1235; Found 284.1236.

3-Butyl-2-(ethylthio)-6-methylbenzo[*b*]furan (**4a**)
3-Butyl-2-(ethylthio)-4-methylbenzo[*b*]furan (**5a**)

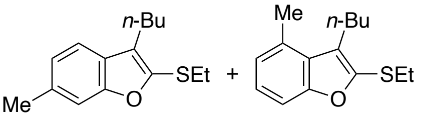

An inseparable mixture of **4a** and **5a** (75:25) was obtained. Pale yellow oil; TLC R_f 0.52 (*n*-hexane/CH_2Cl_2 = 10/1); ^{1}H NMR ($CDCl_3$, 400 MHz) for **4a**: δ 0.95 (t, 3H, *J* = 7.4 Hz), 1.27 (t, 3H, *J* = 7.4 Hz), 1.33–1.44 (m, 2H), 1.54–1.70 (m, 2H), 2.47 (s, 3H), 2.74 (t, 2H, *J* = 7.6 Hz), 2.87 (q, 2H, *J* = 7.4 Hz), 7.05 (d, 1H, *J* = 7.9 Hz), 7.23 (s, 1H), 7.39 (d, 1H, *J* = 7.9 Hz); for **5a**: 0.97 (t, 3H, *J* = 7.4 Hz), 1.29 (t, 3H, *J* = 7.4 Hz), 1.39–1.50 (m, 2H), 1.54–1.70 (m, 2H), 2.63 (s, 3H), 2.85 (t, 2H, *J* = 7.8 Hz), 2.89 (q, 2H, *J* = 7.4 Hz), 6.96 (d, 1H, *J* = 7.7 Hz), 7.15 (dd, 1H, *J* = 7.7, 7.7 Hz) 7.27 (d, 1H, *J* = 7.7 Hz); ^{13}C{^{1}H} NMR ($CDCl_3$, 101 MHz) *for **4a**:* δ 13.9, 15.3, 21.7, 22.5, 24.3, 29.5, 31.8, 111.2, 119.1, 123.6, 125.8, 126.5, 134.9, 144.8, 156.0; for **5a**: 14.0, 15.3, 19.1, 22.6, 25.4, 29.3, 34.0, 108.8, 124.0, 124.3, 126.2, 127.1, 131.1, 145.7, 155.9; HRMS (EI) *m/z*: $[M]^{\cdot+}$ Calcd for $C_{15}H_{20}OS^{\cdot+}$ 248.1235; Found 248.1236.

3-Butyl-2-(ethylthio)-6,7-dihydro-5*H*-indeno[5,6-*b*]furan (**4b**)
1-Butyl-2-(ethylthio)-7,8-dihydro-6*H*-indeno[5,4-*b*]furan (**5b**)

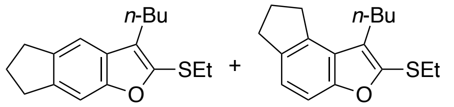

An inseparable mixture of **4b** and **5b** (83:17) was obtained. Colorless oil; TLC R_f 0.48 (*n*-hexane/CH_2Cl_2 = 10/1); ^{1}H NMR ($CDCl_3$, 400 MHz) for **4b**: δ 0.95 (t, 3H, *J* = 7.4 Hz), 1.27 (t, 3H, *J* = 7.4 Hz), 1.32–1.50 (m, 2H), 1.53–1.72 (m, 2H), 2.07–2.19 (m, 2H), 2.73 (t, 2H, *J* = 7.6 Hz), 2.85 (q, 2H, *J* = 7.4 Hz), 2.92–3.03 (m, 4H), 7.26 (s, 1H), 7.32 (s, 1H); for **5b**: 0.95–1.00 (m 3H), 1.28 (t, 3H, *J* = 7.4 Hz), 1.32–1.50 (m, 2H), 1.53–1.72 (m, 2H), 2.14–2.26 (m, 2H), 2.77 (t, 2H, *J* = 7.8 Hz), 2.88 (q, 2H, *J* = 7.4 Hz), 2.92–3.03 (m, 2H), 3.18 (t, 2H, *J* = 7.4 Hz), 7.14 (d, 1H, *J* = 8.2 Hz), 7.22 (d, 1H, *J* = 8.2 Hz); ^{13}C{^{1}H} NMR ($CDCl_3$, 101 MHz) for **4b**: δ 13.9, 15.2, 22.6, 24.4, 26.4, 29.6, 31.9, 32.4, 33.0, 106.8, 114.4, 125.9, 127.5, 138.5, 141.8, 144.7, 155.2; for **5b**: 14.0, 15.3, 22.5, 25.0, 25.8, 29.4, 31.5, 32.2, 33.6, 108.6, 120.5, 125.5, 125.8, 135.6, 138.2, 144.6, 155.1; HRMS (EI) *m/z*: $[M]^{\cdot+}$ Calcd for $C_{17}H_{22}OS^{\cdot+}$ 274.1391; Found 274.1390.

3-Butyl-2-(ethylthio)-5,6-dimethoxybenzo[*b*]furan (**4c**)

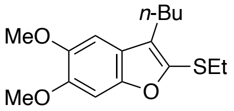

Colorless oil; TLC R_f 0.38 (*n*-hexane/CH_2Cl_2 = 1/1); ^{1}H NMR ($CDCl_3$, 400 MHz): δ 0.95 (t, 3H, *J* = 7.3 Hz), 1.26 (t, 3H, *J* = 7.4 Hz), 1.32–1.45 (m, 2H), 1.57–1.68 (m, 2H), 2.72 (t, 2H, *J* = 7.6 Hz), 2.88 (q, 2H, *J* = 7.4 Hz), 3.91 (s, 3H), 3.93 (s, 3H), 6.90 (s, 1H), 6.99 (s, 1H); ^{13}C{^{1}H} NMR ($CDCl_3$, 101 MHz): δ 13.9, 15.3, 22.5, 24.4, 29.9, 31.9, 56.2, 56.5, 95.1, 101.0, 120.7, 126.4, 144.0, 146.2, 148.4, 150.5; HRMS (ESI) *m/z*: $[M + Na]^+$ Calcd for $C_{16}H_{22}NaO_3S^+$ 317.1187; Found 317.1187.

1-Butyl-2-(ethylthio)naphtho[2,1-*b*]furan (**5d**)

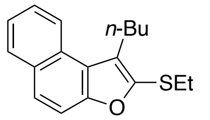

Pale yellow oil; TLC R_f 0.42 (*n*-hexane/CH_2Cl_2 = 10/1); ^{1}H NMR ($CDCl_3$, 400 MHz): δ 1.00 (t, 3H, *J* = 7.4 Hz), 1.31 (t, 3H, *J* = 7.4 Hz), 1.45–1.60 (m, 2H), 1.69–1.81 (m, 2H), 2.91 (t, 2H, *J* = 7.4 Hz), 3.12 (q, 2H, *J* = 7.4 Hz), 7.45–7.52 (m, 1H), 7.56–7.64 (m, 2H), 7.72 (d, 1H, *J* = 8.9 Hz), 7.95 (d, 1H, *J* = 8. 0 Hz), 8.27 (d, 1H, *J* = 8.2 Hz); ^{13}C{^{1}H} NMR ($CDCl_3$, 101 MHz): δ 14.0, 15.3, 22.6, 26.0, 29.9, 32.0, 112.3, 121.9, 123.1, 124.1, 126.0, 126.5, 127.8, 128.1, 129.1, 130.6, 145.2, 153.8; HRMS (EI) *m/z*: $[M]^{\cdot+}$ Calcd for $C_{18}H_{20}OS^{\cdot+}$ 284.1235; Found 284.1224.

((1-Butyl-2-(ethylthio)naphtho[2,1-*b*]furan-8-yl)oxy)triisopropylsilane (**5e**)

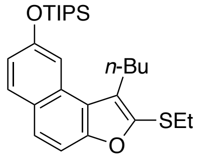

Pale yellow oil; TLC R_f 0.27 (*n*-hexane/CH_2Cl_2 = 10/1); ^{1}H NMR ($CDCl_3$, 400 MHz): δ 0.98 (t, 3H, *J* = 7.4 Hz), 1.16 (d, 18H, *J* = 7.3 Hz), 1.27–1.42 (m, 6H), 1.44–1.58 (m, 2H), 1.67–1.78 (m, 2H), 2.90 (q, 2H, *J* = 7.4 Hz), 3.07 (t, 2H, *J* = 7.8 Hz), 7.11 (dd, 1H, *J* = 8.8, 2.3 Hz), 7.45 (d, 1H, *J* = 8.8 Hz), 7.63 (d, 1H, *J* = 8.8 Hz), 7.68 (d, 1H, *J* = 2.3 Hz), 7.80 (d, 1H, *J* = 8.8 Hz); ^{13}C{^{1}H} NMR ($CDCl_3$, 101 MHz): δ 12.8, 14.1, 15.3, 18.0, 22.7, 26.2, 30.0, 32.5, 110.0, 111.1, 119.6, 121.0, 125.8 (two signals overlapped), 127.7, 129.3, 130.4, 144.6, 154.3, 154.8; HRMS (ESI) *m/z*: $[M + Na]^+$ Calcd for $C_{27}H_{40}NaO_2SSi^+$ 479.2416; Found 479.2408.

2-Ethylthio-3-butylbenzofuro[6,5-*b*]benzofuran (**4f**)

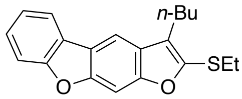

Yellow oil; TLC R_f 0.54 (*n*-hexane/CH_2Cl_2 = 5/1); ^{1}H NMR ($CDCl_3$, 400 MHz): δ 0.98 (t, 3H, *J* = 7.3 Hz), 1.31 (t, 3H, *J* = 7.4 Hz), 1.38–1.50 (m, 2H), 1.67–1.79 (m, 2H), 2.85 (t, 2H, *J* = 7.6 Hz), 2.92 (q, 2H, *J* = 7.4 Hz), 7.35 (dd, 1H, *J* = 7.9, 7.9 Hz), 7.43 (dd, 1H, *J* = 7.9, 7.9 Hz), 7.55 (d, 1H, *J* = 7.9 Hz), 7.59 (d, 1H, *J* = 0.6 Hz), 7.96 (d, 1H, *J* = 0.6 Hz), 7.99 (d, 1H, *J* = 7.9 Hz); $^{13}C\{^1H\}$ NMR ($CDCl_3$, 101 MHz): δ 14.0, 15.3, 22.6, 24.5, 29.5, 31.9, 94.6, 109.9, 111.5, 120.3, 120.5, 122.6, 124.3, 125.5, 125.8, 126.5, 146.0, 154.9, 155.3, 156.8; HRMS (EI) *m/z*: $[M]^{\cdot+}$ Calcd for $C_{20}H_{20}O_2S^{\cdot+}$ 324.1184; Found 324.1183.

2-Ethylthio-3-butylbenzo[*b*]furo[2,3-*g*]benzofuran (**5f**)

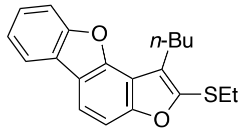

Colorless solid; TLC R_f 0.34 (*n*-hexane/CH_2Cl_2 = 10/1); ^{1}H NMR ($CDCl_3$, 400 MHz): δ 1.01 (t, 3H, *J* = 7.4 Hz), 1.33 (t, 3H, *J* = 7.4 Hz), 1.41–1.54 (m, 2H), 1.79–1.91 (m, 2H), 2.94 (q, 2H, *J* = 7.4 Hz), 3.03 (t, 2H, *J* = 7.5 Hz), 7.37 (dd, 1H, *J* = 7.5, 7.5 Hz), 7.40–7.48 (m, 2H), 7.65 (d, 1H, *J* = 8.0 Hz), 7.82 (d, 1H, *J* = 8.5 Hz), 7.96 (d, 1H, *J* = 7.5 Hz); $^{13}C\{^1H\}$ NMR ($CDCl_3$, 101 MHz): δ 14.0, 15.3, 22.3, 25.2, 29.6, 32.3, 106.7, 111.7, 115.2, 116.3, 118.3, 119.9, 122.9, 124.6, 124.7, 125.7, 145.9, 148.8, 156.2 (two signals overlapped); HRMS (ESI) *m/z*: $[M + K]^+$ Calcd for $C_{20}H_{20}KO_2S^+$ 363.0821; Found 363.0832.

3-Butyl-2-(methylthio)benzo[*b*]furan (**3q**)

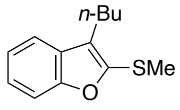

Colorless oil; TLC R_f 0.50 (*n*-hexane/CH_2Cl_2 = 10/1); ^{1}H NMR ($CDCl_3$, 400 MHz): δ 0.95 (t, 3H, *J* = 7.4 Hz), 1.32–1.45 (m, 2H), 1.59–1.71 (m, 2H), 2.47 (s, 3H), 2.75 (t, 2H, *J* = 7.5 Hz), 7.21 (dd, 1H, *J* = 8.2, 8.2 Hz), 7.27 (dd, 1H, *J* = 8.2, 8.2 Hz), 7.42 (d, 1H, *J* = 8.2 Hz), 7.49 (d, 1H, *J* = 8.2 Hz); $^{13}C\{^1H\}$ NMR ($CDCl_3$, 101 MHz): δ 13.9, 18.0, 22.5, 24.1, 31.8, 110.9, 119.5, 122.3, 123.9, 124.4, 129.0, 146.9, 155.5; HRMS (EI) *m/z*: $[M]^{\cdot+}$ Calcd for $C_{13}H_{16}OS^{\cdot+}$ 220.0922; Found 220.0923.

2-(Benzylthio)-3-butylbenzo[*b*]furan (**3r**)

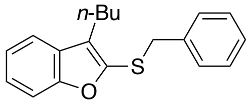

Colorless oil; TLC R_f 0.60 (*n*-hexane/CH_2Cl_2 = 3/1); ^{1}H NMR ($CDCl_3$, 400 MHz): δ 0.85 (t, 3H, *J* = 7.3 Hz), 1.15–1.29 (m, 2H), 1.31–1.43 (m, 2H), 2.47 (t, 2H, *J* = 7.6 Hz), 4.06 (s, 2H), 7.10–7.17 (m, 2H), 7.17–7.25 (m, 4H), 7.26–7.31 (AA'BB'C, 1H), 7.40–7.50 (AA'BB'C, 2H); $^{13}C\{^1H\}$ NMR ($CDCl_3$, 101 MHz): δ 13.8, 22.5, 24.0, 31.5, 39.6, 111.0, 119.7, 122.2, 124.7, 126.7, 127.2, 128.4, 128.8 (two signals overlapped), 137.6, 144.8, 155.7; HRMS (EI) *m/z*: $[M]^{\cdot+}$ Calcd for $C_{19}H_{20}OS^{\cdot+}$ 296.1235; Found 296.1235.

3-Butyl-2-((6-chlorohexyl)thio)benzo[*b*]furan (**3s**)

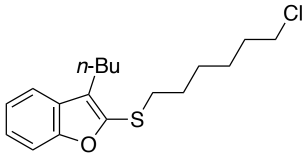

Colorless oil; TLC R_f 0.66 (*n*-hexane/CH_2Cl_2 = 3/1); ^{1}H NMR ($CDCl_3$, 400 MHz): δ 0.94 (t, 3H, *J* = 7.4 Hz), 1.32–1.50 (m, 6H), 1.57–1.70 (m, 4H), 1.70–1.85 (m, 2H), 2.75 (t, 2H, *J* = 7.6 Hz), 2.87 (t, 2H, *J* = 7.3 Hz), 3.51 (t, 2H, *J* = 6.7 Hz), 7.21 (ddd, 1H, *J* = 7.6, 7.6, 1.0 Hz), 7.25–7.30 (m, 1H), 7.40–7.44 (m, 1H), 7.48–7.52 (m, 1H); $^{13}C\{^1H\}$ NMR ($CDCl_3$, 101 MHz): δ 13.9, 22.5, 24.3, 26.4, 27.7, 29.8, 31.8, 32.4, 35.0, 44.9, 111.0, 119.5, 122.3, 124.5, 125.3, 129.0, 145.9, 155.6; HRMS (ESI) *m/z*: $[M + K]^+$ Calcd for $C_{18}H_{25}{}^{35}ClKOS^+$ 363.0952; Found 363.0952.

3-Butyl-2-(ethylsulfinyl)benzo[*b*]furan (**7**)

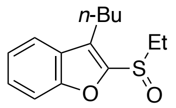

Colorless oil; TLC R_f 0.22 (*n*-hexane/EtOAc= 3/1); ^{1}H NMR ($CDCl_3$, 400 MHz): δ 0.95 (t, 3H, *J* = 7.4 Hz), 1.25 (t, 3H, *J* = 7.5 Hz), 1.34–1.48 (m, 2H), 1.61–1.80 (m, 2H), 2.90 (t, 2H, *J* = 7.6 Hz), 3.26–3.46 (m, 2H), 7.31 (dd, 1H, *J* = 8.3, 8.3 Hz), 7.43 (dd, 1H, *J* = 8.3, 8.3 Hz), 7.54 (d, 1H, *J* = 8.3 Hz), 7.63 (d, 1H, *J* = 8.3 Hz); ^{13}C{^{1}H} NMR ($CDCl_3$, 101 MHz): δ 7.5, 13.8, 22.5, 23.3, 32.2, 45.7, 112.3, 121.1, 123.3, 127.3, 127.5, 128.8, 147.9, 155.7; HRMS (ESI) *m/z*: $[M + Na]^+$ Calcd for $C_{14}H_{18}NaO_2S^+$ 273.0925; Found 273.0926.

3-Butyl-2-(ethylsulfonyl)benzo[*b*]furan (**8**)

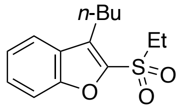

Colorless oil; TLC R_f 0.63 (*n*-hexane/EtOAc = 3/1); ^{1}H NMR ($CDCl_3$, 400 MHz): δ 0.95 (t, 3H, *J* = 7.4 Hz), 1.35 (t, 3H, *J* = 7.5 Hz), 1.39–1.50 (m, 2H), 1.65–1.78 (m, 2H), 3.06 (t, 2H, *J* = 7.8 Hz), 3.20 (q, 2H, *J* = 7.5 Hz), 7.35 (dd, 1H, *J* = 8.0, 8.0 Hz), 7.49 (dd, 1H, *J* = 8.0, 8.0 Hz), 7.54 (d, 1H, *J* = 8.0 Hz), 7.69 (d, 1H, *J* = 8.0 Hz); ^{13}C{^{1}H} NMR ($CDCl_3$, 101 MHz): δ 7.1, 13.9, 22.7, 23.1, 32.5, 49.9, 112.4, 121.7, 123.7, 127.5, 128.2, 130.1, 142.9, 154.7; HRMS (ESI) *m/z*: $[M + Na]^+$ Calcd for $C_{14}H_{18}NaO_3S^+$ 289.0874; Found 289.0874.

3-Butyl-2-(2-(ethylthio)-3-methoxyphenoxy)benzo[*b*]furan (**12**)

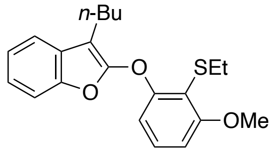

Colorless oil; TLC R_f 0.65 (*n*-hexane/CH_2Cl_2 = 1/1); ^{1}H NMR ($CDCl_3$, 400 MHz): δ 0.88 (t, 3H, *J* = 7.4 Hz), 1.25 (t, 3H, *J* = 7.4 Hz), 1.30–1.42 (m, 2H), 1.58–1.69 (m, 2H), 2.59 (t, 2H, *J* = 7.6 Hz), 2.96 (q, 2H, *J* = 7.4 Hz), 3.93 (s, 3H), 6.48 (dd, 1H, *J* = 8.3, 1.0 Hz), 6.69 (dd, 1H, *J* = 8.3, 1.0 Hz), 7.17 (dd, 1H, *J* = 8.3, 8.3 Hz), 7.20–7.26 (m, 2H), 7.30–7.37 (m, 1H), 7.45–7.53 (m, 1H); ^{13}C{^{1}H} NMR ($CDCl_3$, 101 MHz): δ 13.8, 14.9, 21.9, 22.5, 28.4, 31.0, 56.3, 100.8, 106.3, 108.4, 110.8, 111.9, 119.2, 122.6, 123.1, 129.3, 129.6, 149.3, 153.5, 158.6, 161.3; HRMS (EI) *m/z*: $[M]^{\cdot+}$ Calcd for $C_{21}H_{24}O_3S^{\cdot+}$ 356.1446; Found 356.1448.

The regiochemistry of **12** was determined by the HMBC and the NOESY experiments.

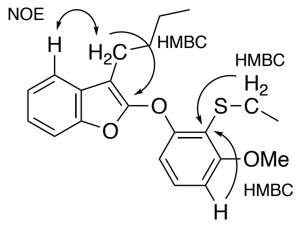

Hex-1-yn-1-yl 4-tolyl sulfide

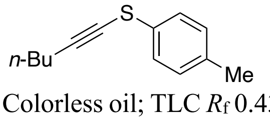

Colorless oil; TLC R_f 0.43 (*n*-hexane); ^{1}H NMR ($CDCl_3$, 400 MHz): δ 0.94 (t, 3H, *J* = 7.2 Hz), 1.40–1.52 (m, 2H), 1.53–1.63 (m, 2H), 2.32 (s, 3H), 2.44 (q, 2H, *J* = 7.0 Hz), 7.10–7.17 (AA'BB', 2H), 7.27–7.34 (AA'BB', 2H); ^{13}C{^{1}H} NMR ($CDCl_3$, 101 MHz): δ 13.6, 20.0, 20.9, 22.0, 30.7, 65.0, 99.3, 126.0, 129.8, 130.0, 136.0; HRMS (EI) *m/z*: $[M]^{\cdot+}$ Calcd for $C_{13}H_{16}S^{\cdot+}$ 204.0973; Found 204.0973.

4-Bromophenyl hex-1-yn-1-yl sulfide

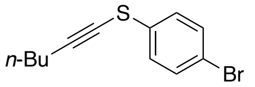

Colorless oil; TLC R_f 0.51 (*n*-hexane); ^{1}H NMR ($CDCl_3$, 400 MHz): δ 0.94 (t, 3H, *J* = 7.2 Hz), 1.39–1.52 (m, 2H), 1.53–1.64 (m, 2H), 2.45 (q, 2H, *J* = 7.1 Hz), 7.24–7.30 (AA'BB', 2H), 7.40–7.46 (AA'BB', 2H); ^{13}C{^{1}H} NMR ($CDCl_3$, 101 MHz): δ 13.6, 20.0, 22.0, 30.6, 63.8, 100.8, 119.7, 127.2, 132.0, 133.1; HRMS (EI) *m/z*: $[M]^{\cdot+}$ Calcd for $C_{12}H_{13}{}^{79}BrS^{\cdot+}$ 267.9921; Found 267.9921.

6-Chlorohexyl hex-1-yn-1-yl sulfide

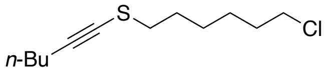

Colorless oil; TLC R_f 0.23 (*n*-hexane); ^{1}H NMR ($CDCl_3$, 400 MHz): δ 0.91 (t, 3H, *J* = 7.2 Hz), 1.34–1.54 (m, 8H), 1.67–1.87 (m, 4H), 2.30 (t, 2H, *J* = 7.9 Hz), 2.66 (t, 2H, *J* = 7.2 Hz), 3.54 (t, 2H, *J* = 6.7 Hz); ^{13}C{^{1}H} NMR ($CDCl_3$, 101 MHz): δ 13.6, 19.8, 21.9, 26.4, 27.4, 28.9, 30.8, 32.4, 35.2, 45.0, 68.0, 94.4; HRMS (EI) *m/z*: $[M]^{\cdot+}$ Calcd for $C_{12}H_{21}{}^{35}ClS^{\cdot+}$ 232.1053; Found 232.1050.

(4-(Ethylsulfinyl)but-3-yn-1-yl)benzene (**2b**)

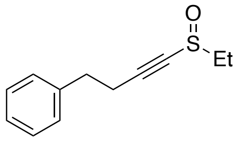

Colorless oil; TLC R_f 0.19 (CH_2Cl_2); ^{1}H NMR ($CDCl_3$, 400 MHz): δ 1.35 (t, 3H, *J* = 7.4 Hz), 2.73 (t, 2H, *J* = 7.4 Hz), 2.83–3.10 (m, 4H), 7.15–7.27 (m, 3H), 7.27–7.35 (AA'BB'C, 2H); ^{13}C{^{1}H} NMR ($CDCl_3$, 101 MHz): δ 6.3, 21.7, 33.8, 49.9, 77.4, 104.1, 126.6, 128.3, 128.5, 139.3; HRMS (ESI) *m/z*: $[M + Na]^+$ Calcd for $C_{12}H_{14}NaOS^+$ 229.0663; Found 229.0664.

4-(Ethylsulfinyl)but-3-yn-1-yl 4-methylbenzoate (**2c**)

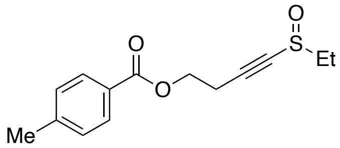

Colorless oil; TLC R_f 0.14 (CH_2Cl_2); ^{1}H NMR ($CDCl_3$, 400 MHz): δ 1.39 (t, 3H, *J* = 7.4 Hz), 2.40 (s, 3H), 2.86–3.14 (m, 4H), 4.44 (t, 2H, *J* = 6.6 Hz), 7.18–7.28 (AA'BB', 2H), 7.86–7.96 (AA'BB', 2H); ^{13}C{^{1}H} NMR ($CDCl_3$, 101 MHz): δ 6.3, 20.3, 21.6, 49.9, 61.2, 78.4, 100.4, 126.7, 129.1, 129.6, 144.0, 166.1; HRMS (ESI) *m/z*: $[M + Na]^+$ Calcd for $C_{14}H_{16}NaO_3S^+$ 287.0718; Found 287.0718.

(2-((3-(Ethylsulfinyl)prop-2-yn-1-yl)oxy)ethyl)benzene (**2d**)

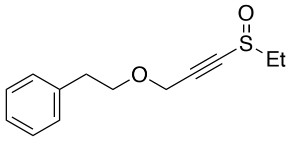

Colorless oil; TLC R_f 0.22 (CH_2Cl_2); ^{1}H NMR ($CDCl_3$, 400 MHz): δ 1.44 (t, 3H, *J* = 7.4 Hz), 2.91 (t, 2H, *J* = 6.9 Hz), 2.95–3.18 (m, 2H), 3.77 (t, 2H, *J* = 6.9 Hz), 4.35 (s, 2H), 7.18–7.25 (m, 3H), 7.27–7.35 (AA'BB'C, 2H); ^{13}C{^{1}H} NMR ($CDCl_3$, 101 MHz): δ 6.5, 36.0, 49.8, 58.3, 71.4, 82.6, 99.3, 126.4, 128.4, 128.8, 138.3; HRMS (ESI) *m/z*: $[M + Na]^+$ Calcd for $C_{13}H_{16}NaO_2S^+$ 259.0769; Found 259.0769.

1-((Ethylsulfinyl)ethynyl)-4-methylbenzene (**2e**)

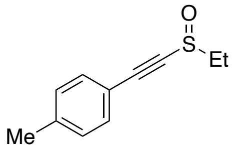

Yellow oil; TLC R_f 0.38 (*n*-hexane/EtOAc = 3/1); ^{1}H NMR ($CDCl_3$, 400 MHz): δ 1.50 (t, 3H, *J* = 7.4 Hz), 2.38 (s, 3H), 3.04–3.26 (m, 2H), 7.15–7.22 (AA'BB', 2H), 7.39–7.46 (AA'BB', 2H); ^{13}C{^{1}H} NMR ($CDCl_3$, 101 MHz): δ 6.6, 21.7, 50.0, 84.1, 102.6, 116.7, 129.4, 132.2, 141.2; HRMS (ESI) *m/z*: $[M + Na]^+$ Calcd for $C_{11}H_{12}NaOS^+$ 215.0507; Found 215.0506.

1-Chloro-4-((ethylsulfinyl)ethynyl)benzene (**2f**)

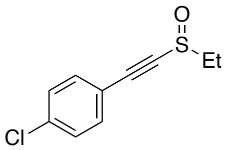

Yellow oil; TLC R_f 0.33 (CH_2Cl_2); ^{1}H NMR ($CDCl_3$, 400 MHz): δ 1.50 (t, 3H, J = 7.4 Hz), 3.05–3.27 (m, 2H), 7.31–7.40 (AA'BB', 2H), 7.43–7.49 (AA'BB', 2H); ^{13}C{^{1}H} NMR ($CDCl_3$, 101 MHz): δ 6.6, 50.0, 85.8, 100.8, 118.2, 129.1, 133.4, 136.9; HRMS (ESI) *m/z*: $[M + Na]^+$ Calcd for $C_{10}H_9{}^{35}ClNaOS^+$ 234.9960; Found 234.9962.

1-(Hex-1-yn-1-ylsulfinyl)-4-methylbenzene (**2g**)

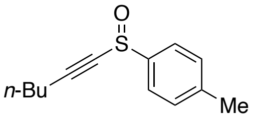

Colorless oil; TLC R_f 0.28 (*n*-hexane/EtOAc = 5/1); ^{1}H NMR ($CDCl_3$, 400 MHz): δ 0.89 (t, 3H, J = 7.3 Hz), 1.32–1.45 (m, 2H), 1.48–1.60 (m, 2H), 2.37–2.47 (m, 5H), 7.28–7.37 (AA'BB', 2H), 7.62–7.72 (AA'BB', 2H); ^{13}C{^{1}H} NMR ($CDCl_3$, 101 MHz): δ 13.4, 19.4, 21.4, 21.8, 29.5, 78.1, 105.9, 125.1, 130.1, 141.2, 142.2; HRMS (ESI) *m/z*: $[M + Na]^+$ Calcd for $C_{13}H_{16}NaOS^+$ 243.0820; Found 243.0821.

1-Bromo-4-(hex-1-yn-1-ylsulfinyl)benzene (**2h**)

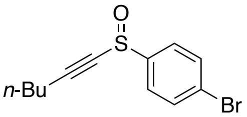

Colorless oil; TLC R_f 0.20 (*n*-hexane/EtOAc = 5/1); ^{1}H NMR ($CDCl_3$, 400 MHz): δ 0.89 (t, 3H, J = 7.3 Hz), 1.31–1.44 (m, 2H), 1.48–1.60 (m, 2H), 2.41 (t, 2H, J = 7.1 Hz), 7.60–7.70 (m, 4H); ^{13}C{^{1}H} NMR ($CDCl_3$, 101 MHz): δ 13.4, 19.4, 21.9, 29.4, 77.7, 106.7, 126.0, 126.4, 132.6, 143.4; HRMS (ESI) *m/z*: $[M + Na]^+$ Calcd for $C_{12}H_{13}{}^{79}BrNaOS^+$ 306.9768; Found 306.9769.

((Hex-1-yn-1-ylsulfinyl)methyl)benzene (**2j**)

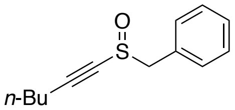

Colorless oil; TLC R_f 0.31 (*n*-hexane/EtOAc = 2/1); ^{1}H NMR ($CDCl_3$, 400 MHz): δ 0.90 (t, 3H, J = 7.3 Hz), 1.30–1.42 (m, 2H), 1.45–1.56 (m, 2H), 2.38 (t, 2H, J = 7.0 Hz), 4.26 (s, 2H), 7.28–7.43 (m, 5H); ^{13}C{^{1}H} NMR ($CDCl_3$, 101 MHz): δ 13.4, 19.3, 21.8, 29.5, 62.8, 76.7, 106.9, 128.6 (two signals overlapped), 129.2, 130.4; HRMS (ESI) *m/z*: $[M + Na]^+$ Calcd for $C_{13}H_{16}NaOS^+$ 243.0820; Found 243.0820.

1-((6-Chlorohexyl)sulfinyl)hex-1-yne (**2k**)

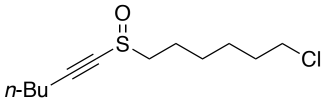

Colorless oil; TLC R_f 0.32 (*n*-hexane/EtOAc = 2/1); ^{1}H NMR ($CDCl_3$, 400 MHz): δ 0.91 (t, 3H, J = 7.4 Hz), 1.35–1.62 (m, 8H), 1.70–1.98 (m, 4H), 2.43 (t, 2H, J = 7.1 Hz), 3.01 (t, 2H, J = 7.6 Hz), 3.53 (t, 2H, J = 6.6 Hz); ^{13}C{^{1}H} NMR ($CDCl_3$, 101 MHz): δ 13.4, 19.3, 21.9, 22.1, 26.4, 27.8, 29.6, 32.1, 44.8, 56.1, 76.8, 105.6; HRMS (ESI) *m/z*: $[M + Na]^+$ Calcd for $C_{12}H_{21}{}^{35}ClNaOS^+$ 271.0899; Found 271.0899.

References for Supporting Information

S1 Z. Xu, H. Chen, Z. Wang, A. Ying, L. Zhang, *J. Am. Chem. Soc.* **2016**, *138*, 5515.

S2 D.-Q. Chen, C.-H. Guo, H.-R. Zhang, D.-P. Jin, X.-S. Li, P. Gao, X.-X. Wu, X.-Y. Liu Y.-M. Lianga, *Green Chem.* **2016**, *18*, 4176.

S3 K. Kanemoto, S.Yoshida, T. Hosoya, *Org. Lett.* **2019**, *21*, 3172.

S4 T. Matsuzawa, T. Hosoya, S. Yoshida, *Chem. Sci.* **2020**, *11*, 9691.

S5 C. Medena, C. Aubert, E. Derat, L. Fensterbank, G. Gontard, O.Khaled, C. Ollivier, N. Vanthuyne, M. Petit, M. Barbazanges, *ChemCatChem* **2021**, *13*, 4543.

S6 S. Yoshida, K. Shimomori, T. Nonaka, T. Hosoya, *Chem. Lett.* **2015**, *44*, 1324.

S7 J. Doroszuk, M. Musiejuk, Ł. Ponikiewski, D. Witt, *Eur. J. Org. Chem.* **2018**, 6333.

S8 M. J. Barrett, G. F. Khan, P. W. Davies, R. S. Grainger, *Chem. Commun.* **2017**, *53*, 5733.

S9 J. Kikuchi, K. Takano, Y. Ota, S. Umemiya, M. Terada, *Chem. Eur J.* **2020**, 26, 11124.

S10 W. Zheng, F. Zheng, Y. Hong, L. Hu, *Heteroat. Chem.* **2012**, *23*, 105.

S11 W. E. Truce, M. J. Lusch, *J. Org. Chem.* **1978**, *43*, 2252.

S12 S. Meng, Y. Wang, J. Liu, J. Zheng, X. Qian, Q. Wang, *Org. Lett.* **2022**, *24*, 757.

^{1}H and ^{13}C NMR Spectra of Compounds

^{1}H NMR (400 MHz) and ^{13}C NMR (101 MHz) spectra of 3-butyl-2-(ethylthio)benzo[*b*]furan (**3a**) ($CDCl_3$)

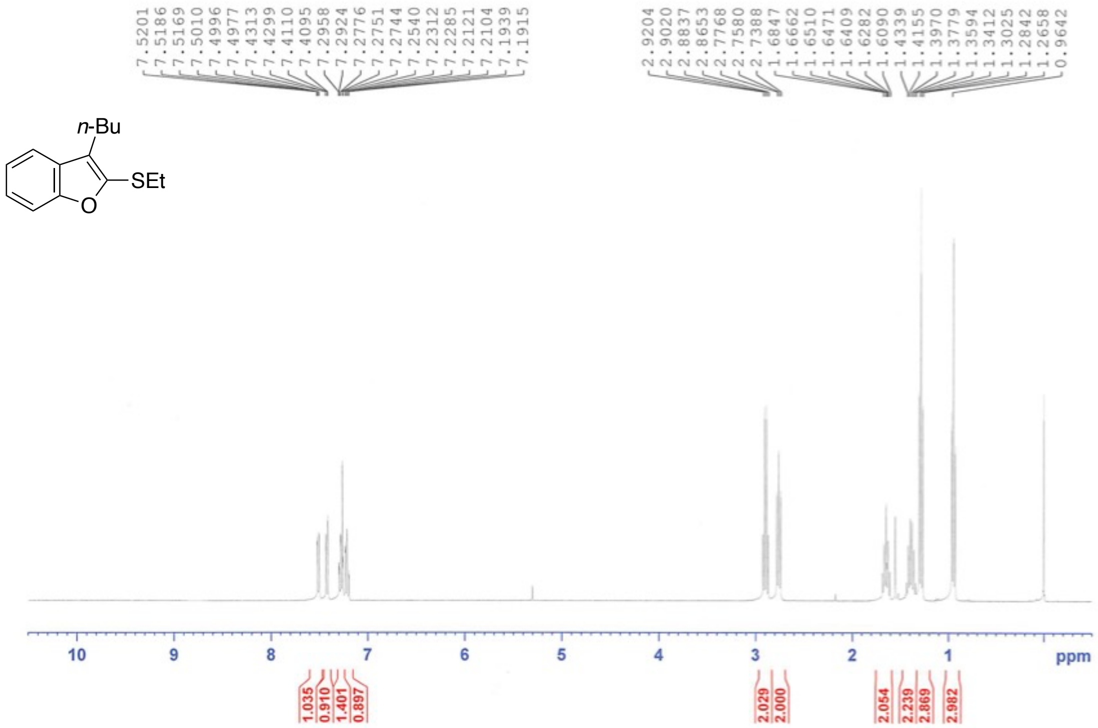

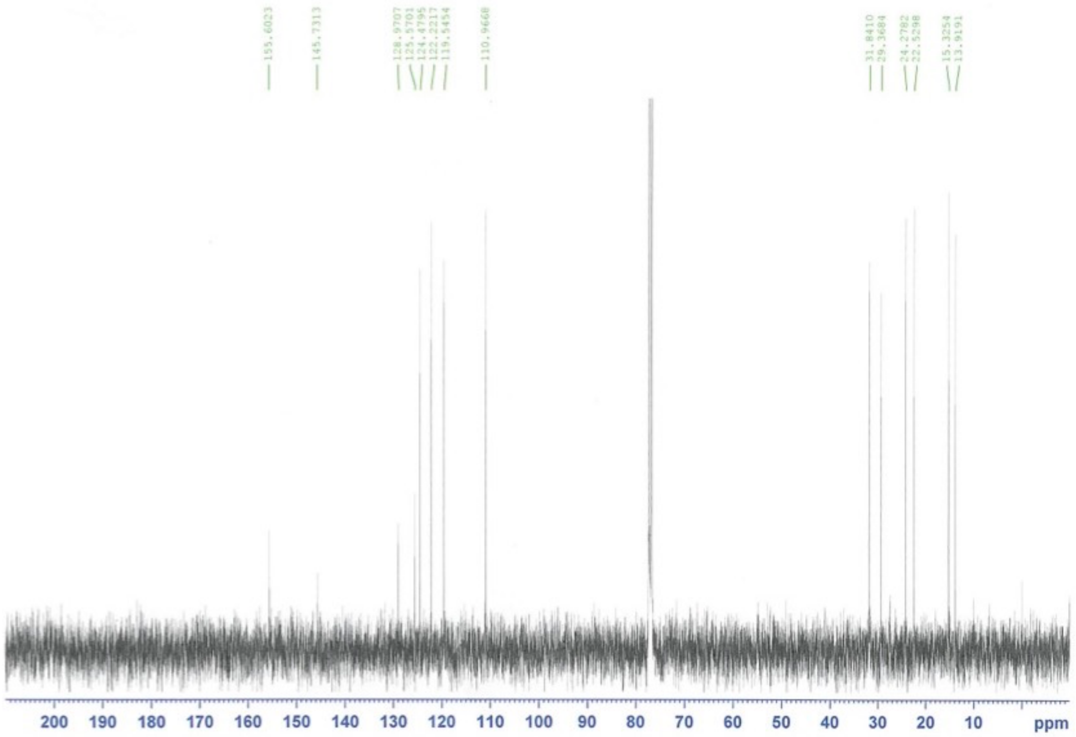

^{1}H NMR (400 MHz) and ^{13}C NMR (101 MHz) spectra of 2-(ethylthio)-3-phenethylbenzo[*b*]furan (**3b**) ($CDCl_3$)

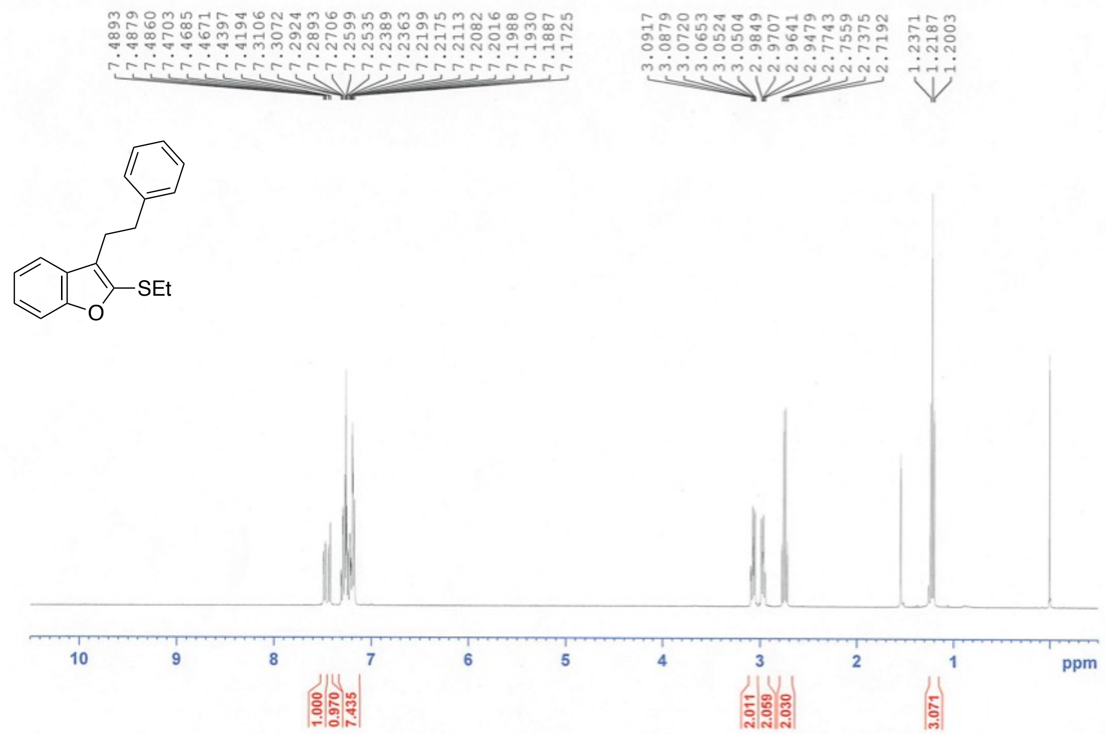

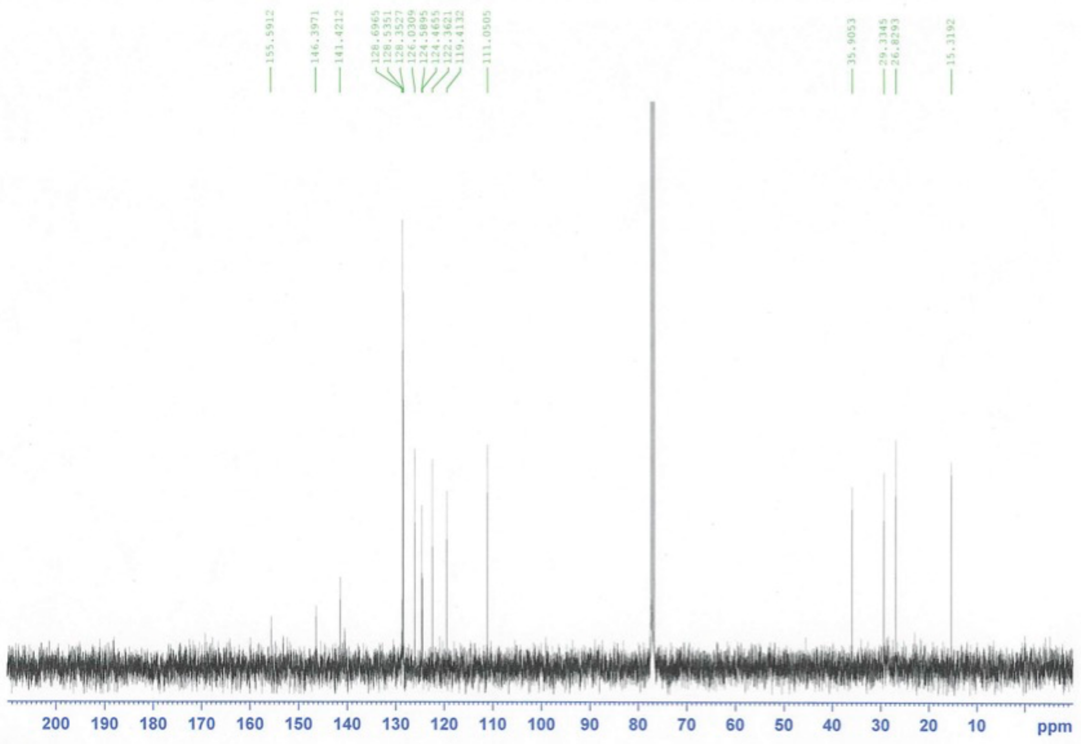

^{1}H NMR (400 MHz) and ^{13}C NMR (101 MHz) spectra of 2-(2-(ethylthio)benzo[*b*]furan-3-yl)ethyl 4-methylbenzoate (**3c**) ($CDCl_3$)

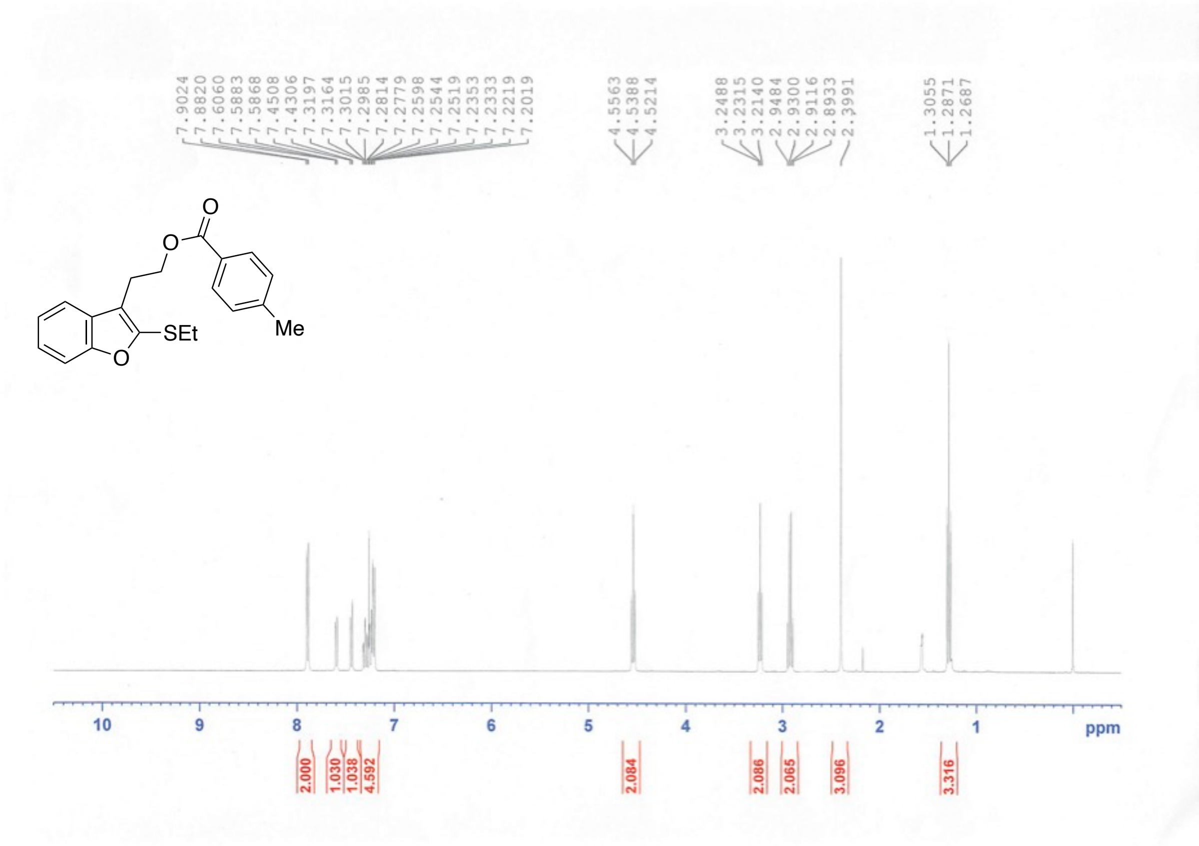

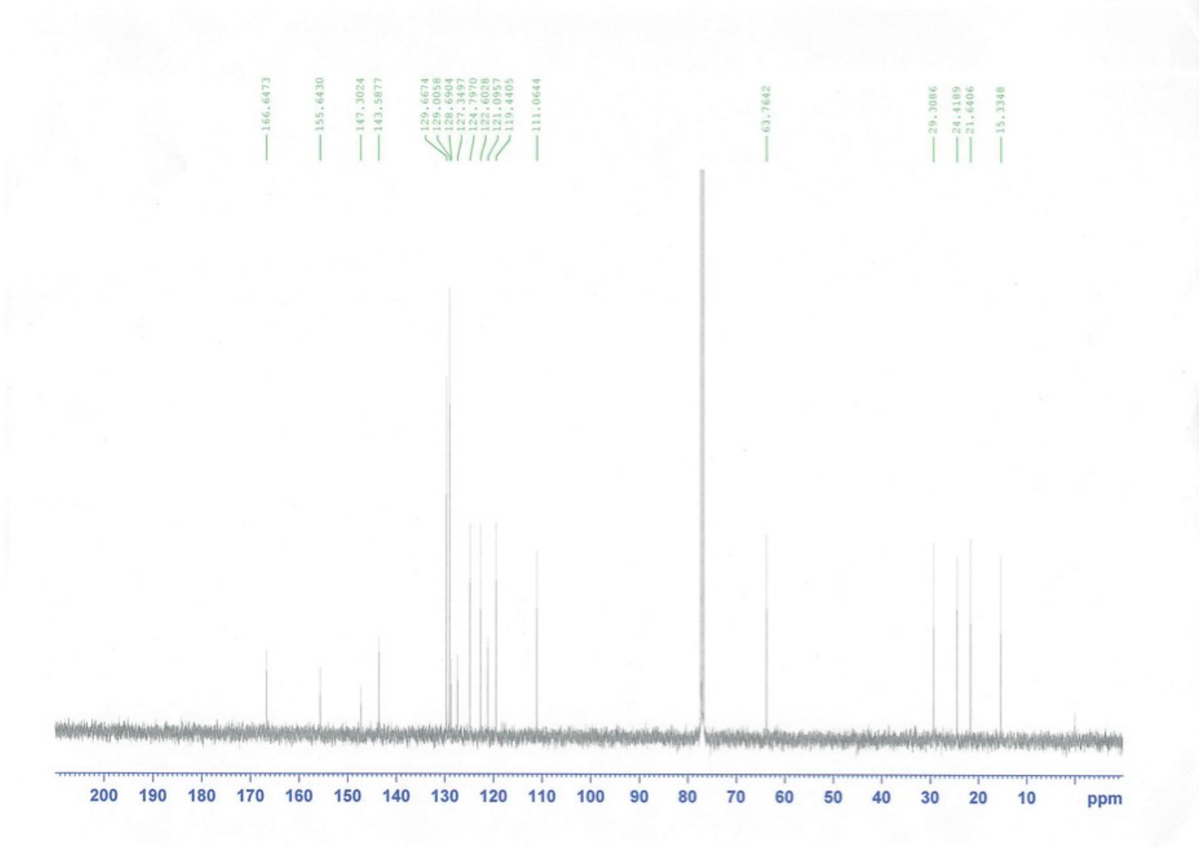

^{1}H NMR (400 MHz) and ^{13}C NMR (101 MHz) spectra of 2-(ethylthio)-3-(phenethoxymethyl)benzo[*b*]furan (**3d**) ($CDCl_3$)

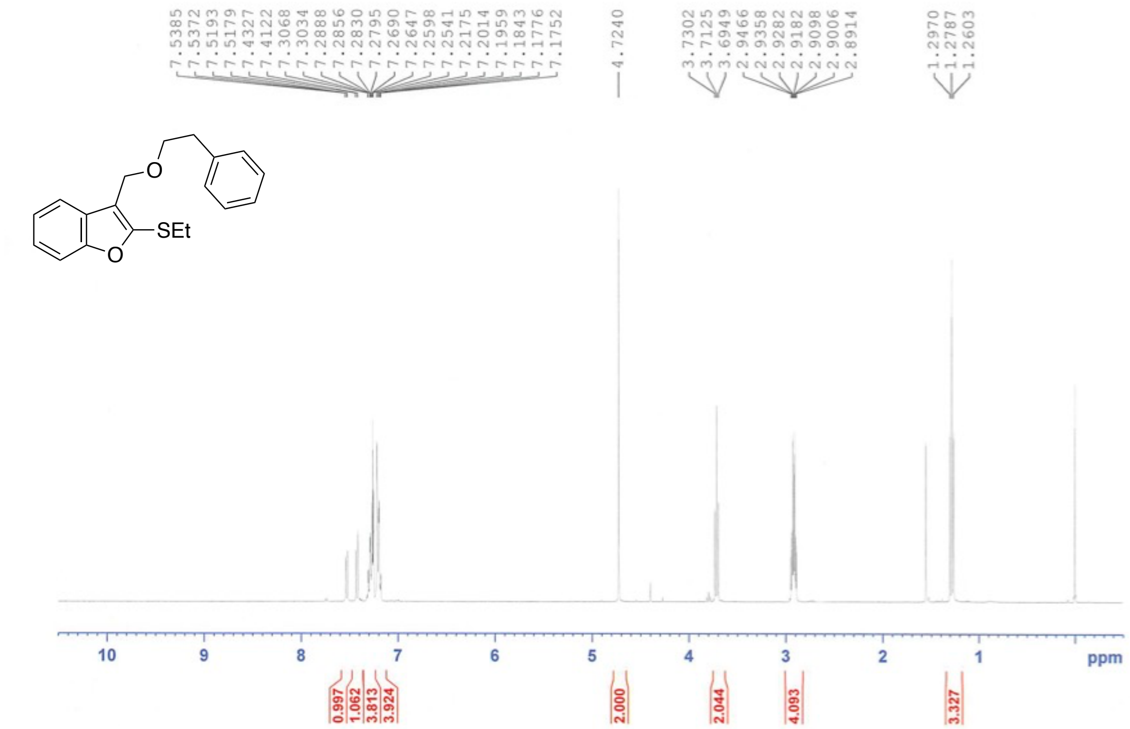

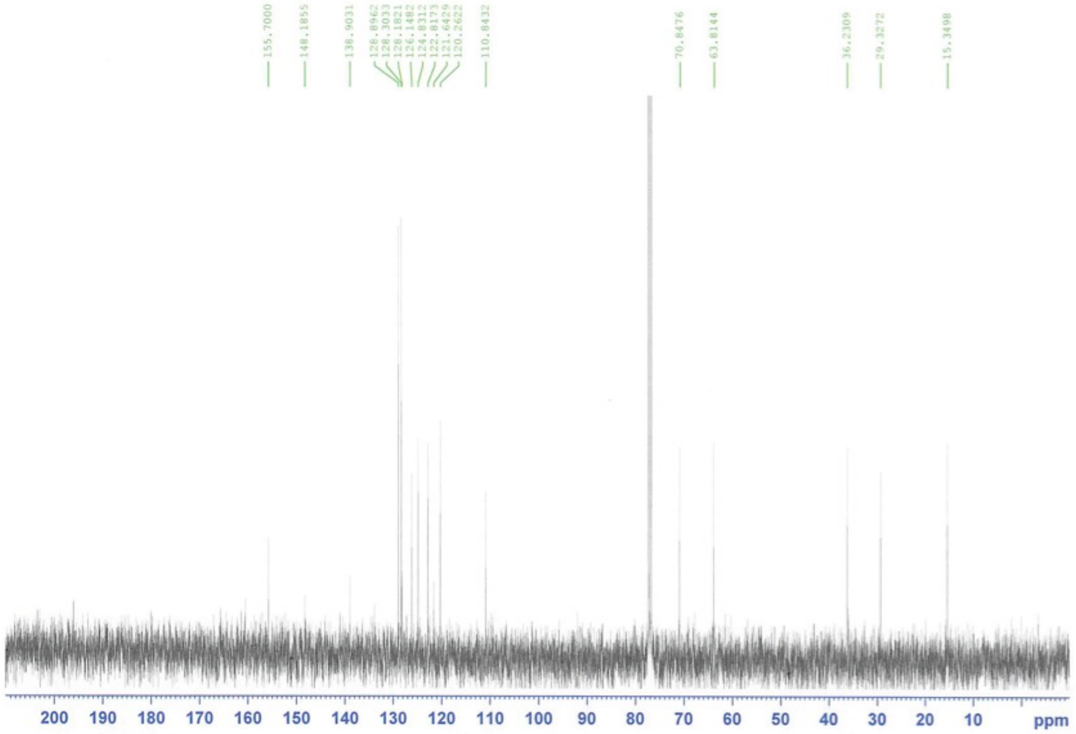

^{1}H NMR (400 MHz) and ^{13}C NMR (101 MHz) spectra of 2-(ethylthio)-3-(4-tolyl)benzo[*b*]furan (**3e**) ($CDCl_3$)

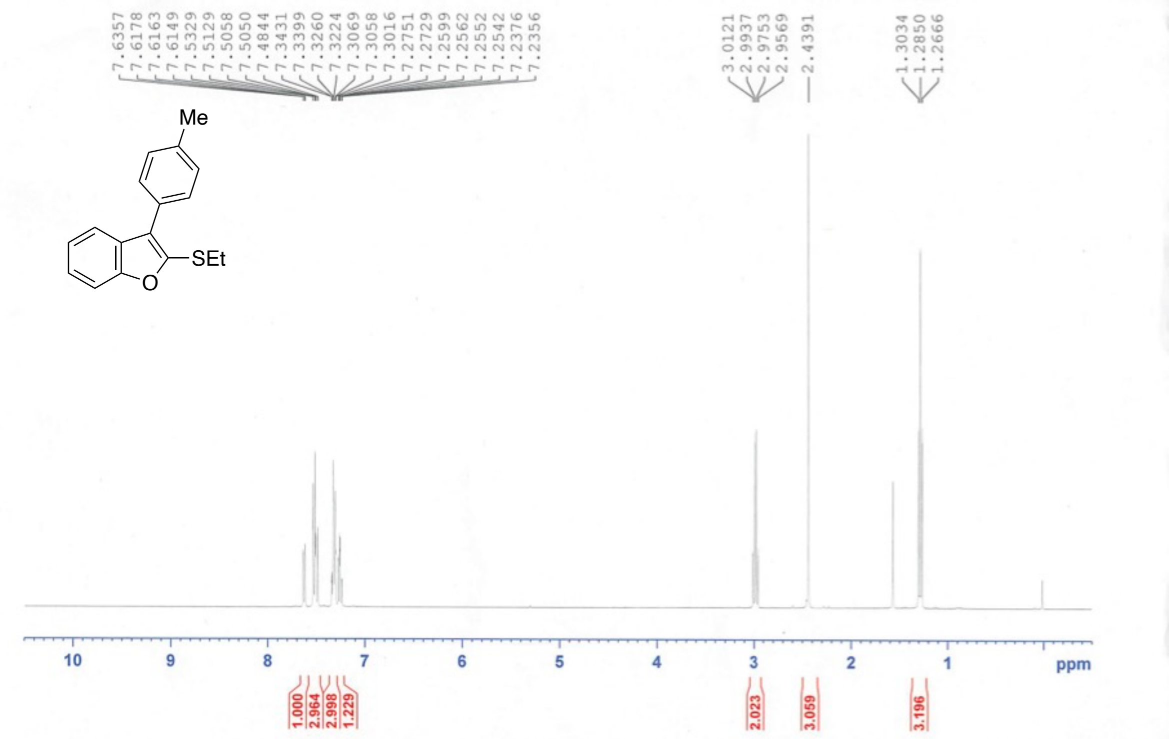

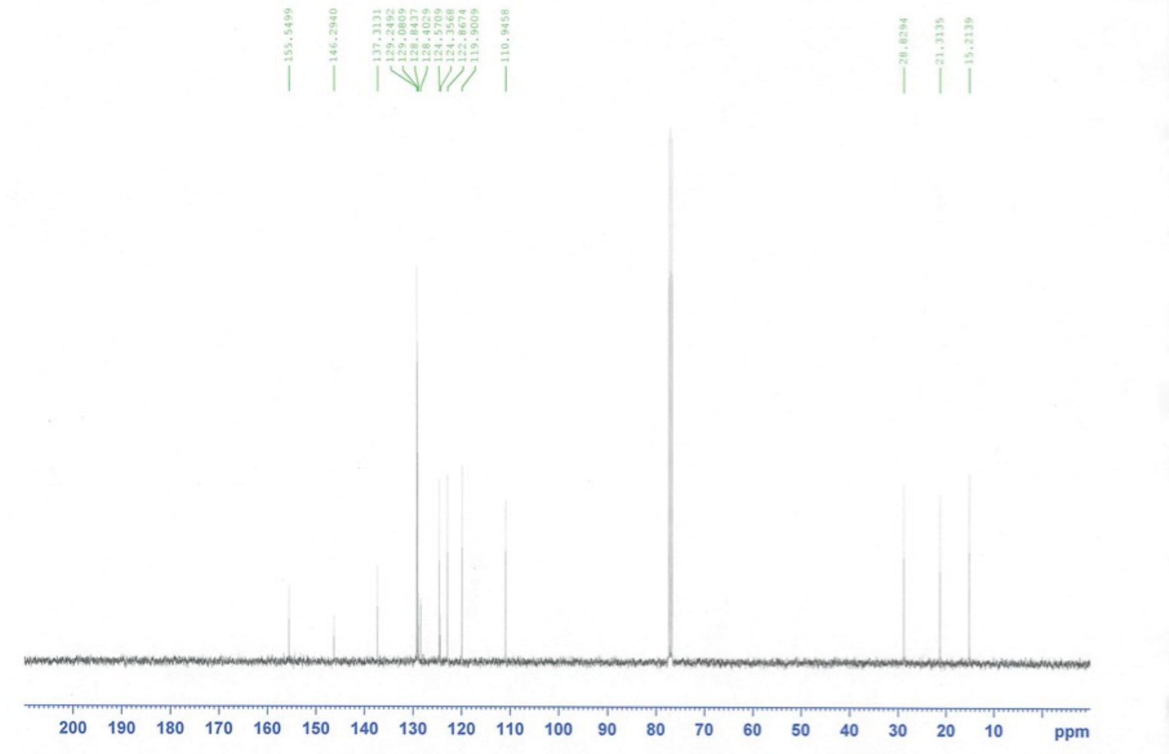

^{1}H NMR (400 MHz) and ^{13}C NMR (101 MHz) spectra of 3-(4-chlorophenyl)-2-(ethylthio)benzo[*b*]furan (**3f**) ($CDCl_3$)

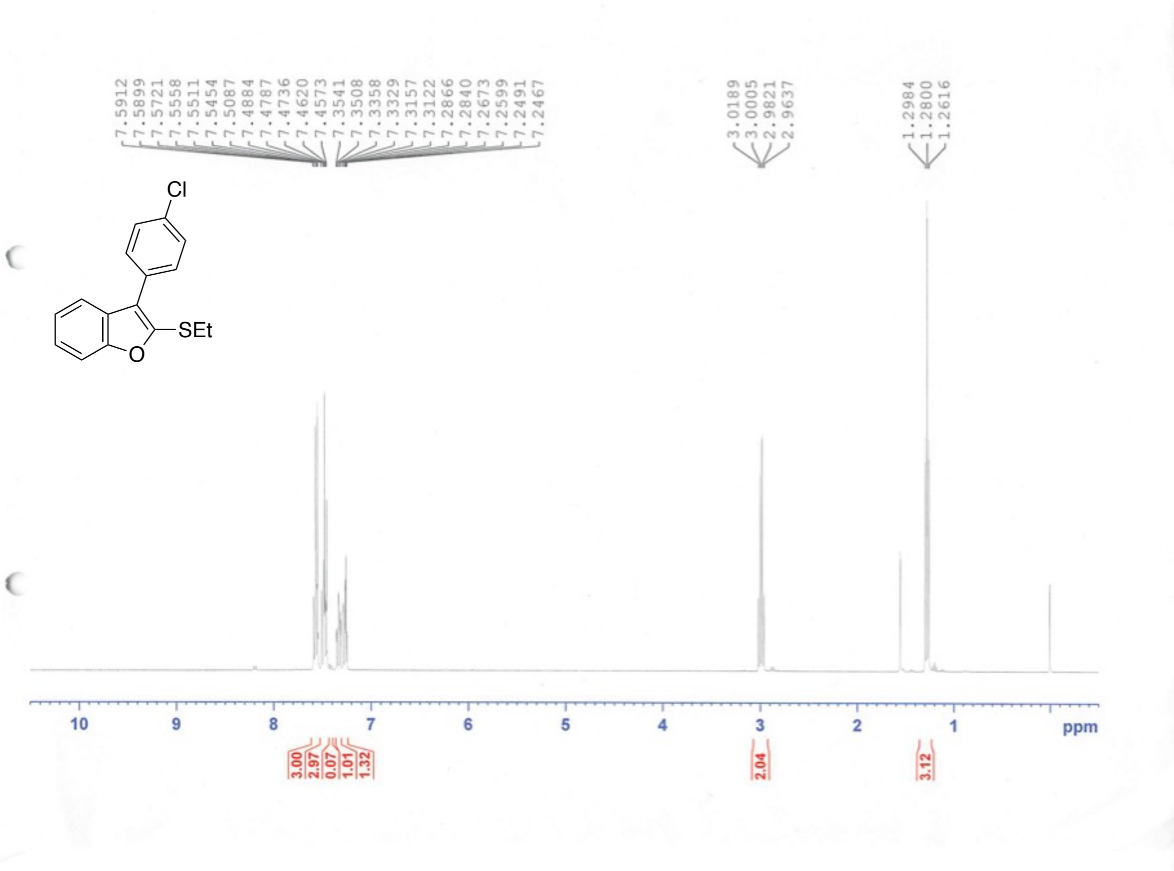

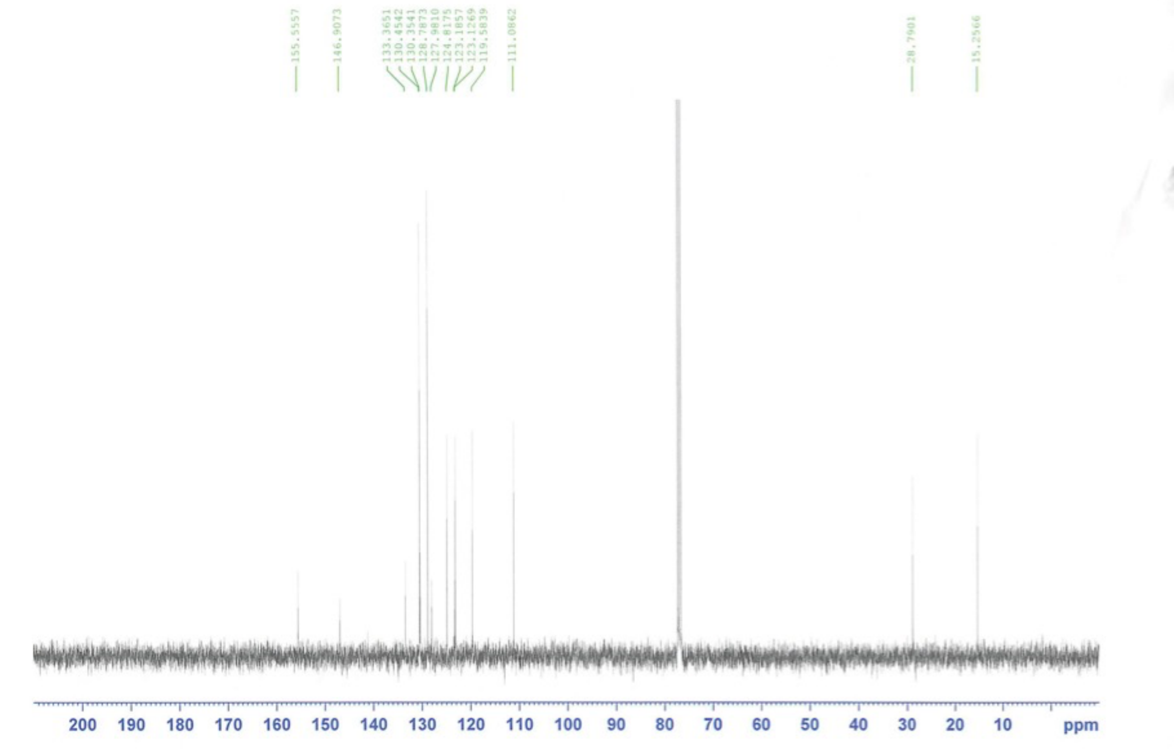

^{1}H NMR (400 MHz) and ^{13}C NMR (101 MHz) spectra of 3-butyl-2-(4-tolylthio)benzo[*b*]furan (**3g**) ($CDCl_3$)

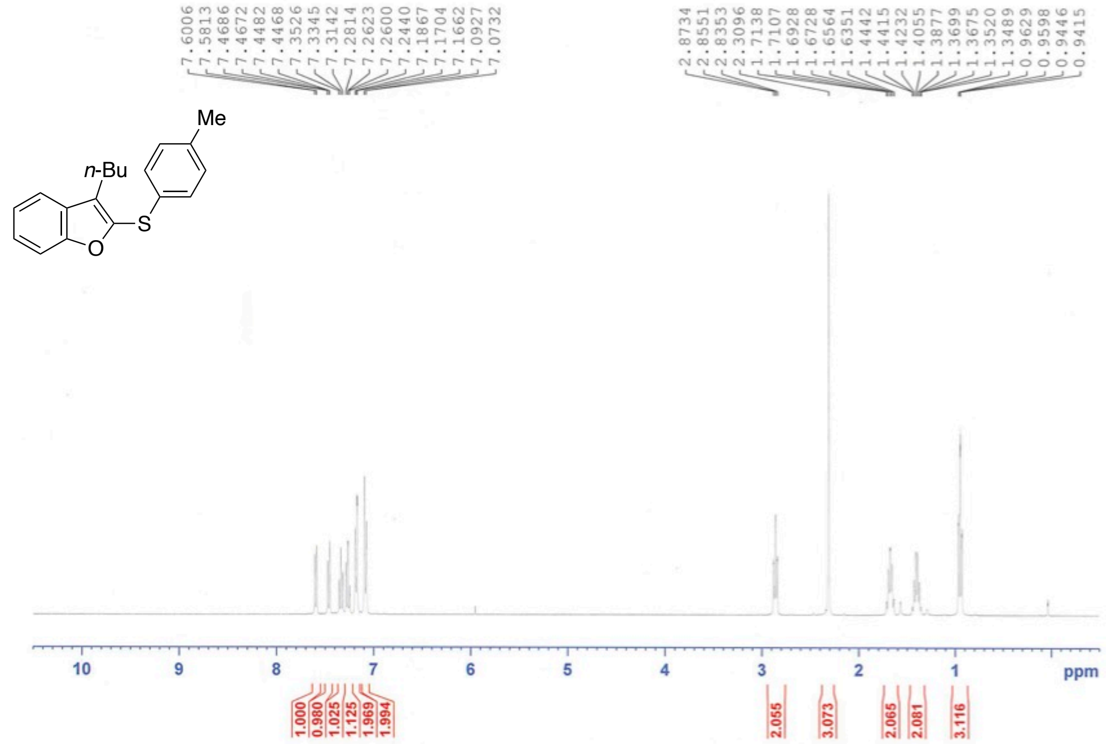

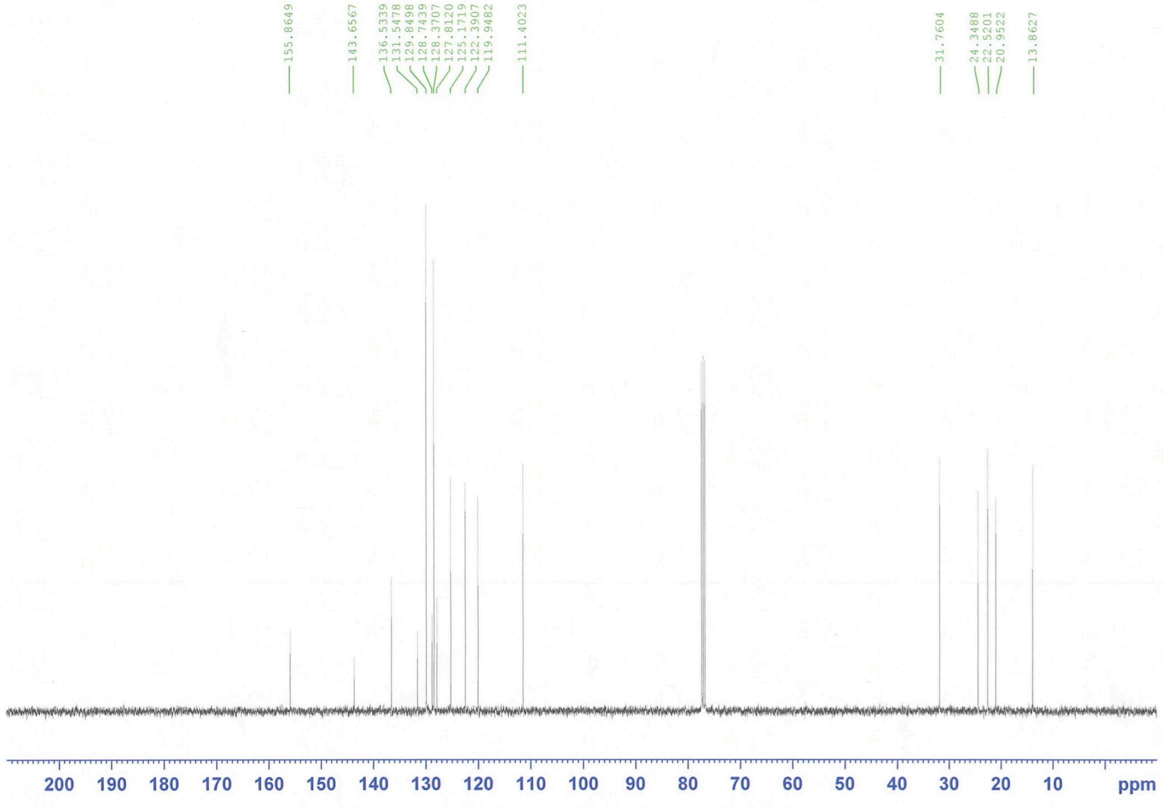

^{1}H NMR (400 MHz) and ^{13}C NMR (101 MHz) spectra of 2-((4-bromophenyl)thio)-3-butylbenzo[*b*]furan (**3h**) ($CDCl_3$)

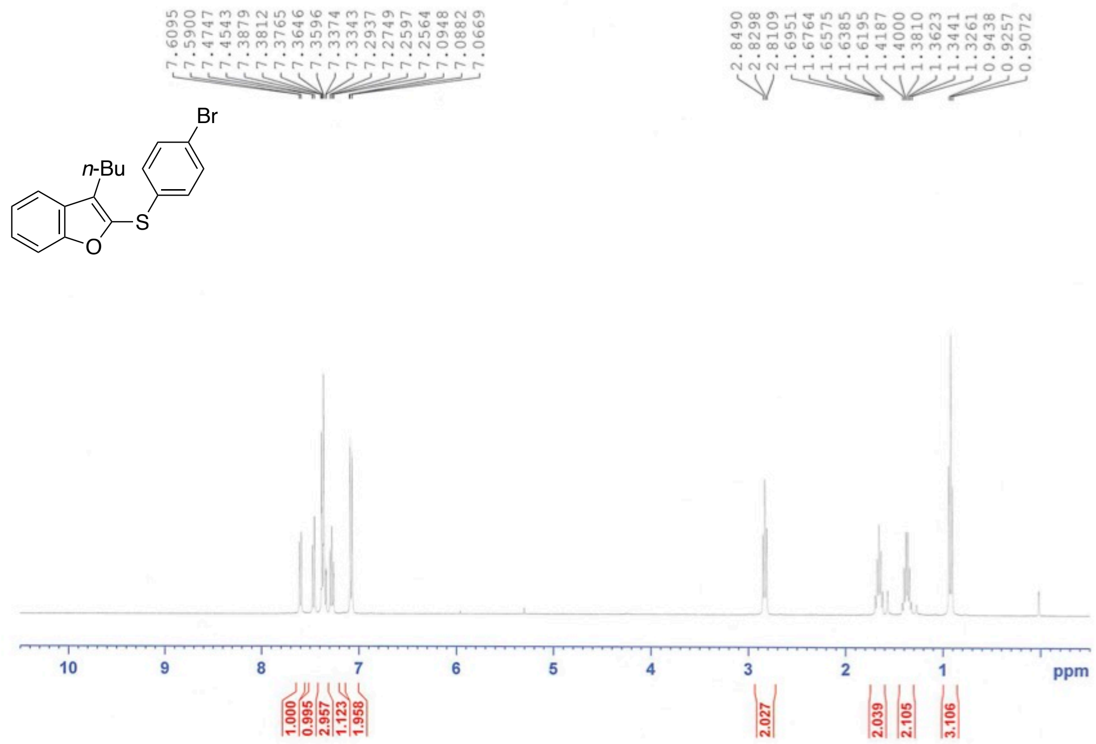

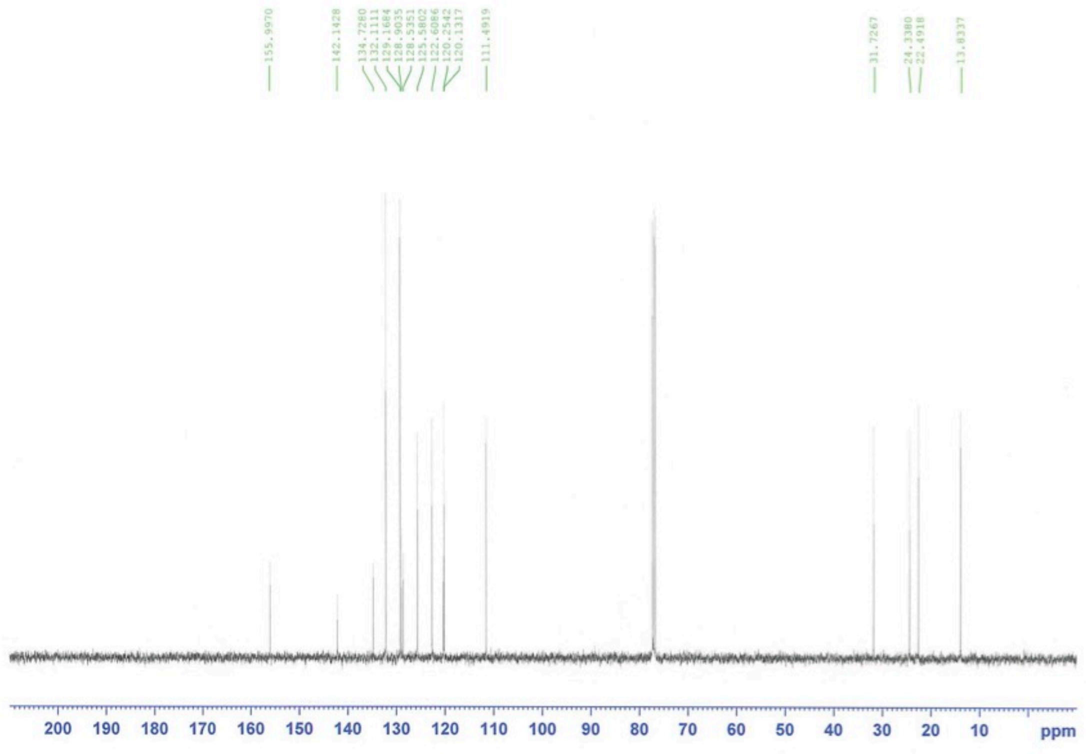

^{1}H NMR (400 MHz) and ^{13}C NMR (101 MHz) spectra of 3-butyl-2-(ethylthio)-5-methylbenzo[*b*]furan (**3i**) ($CDCl_3$)

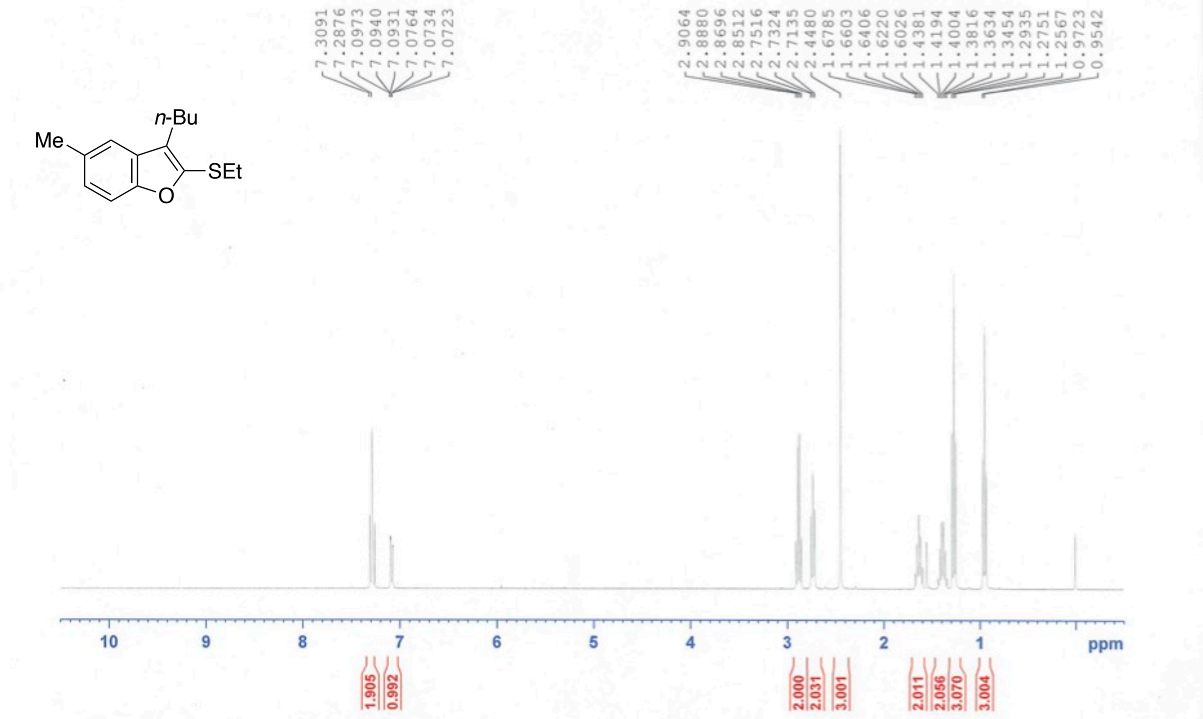

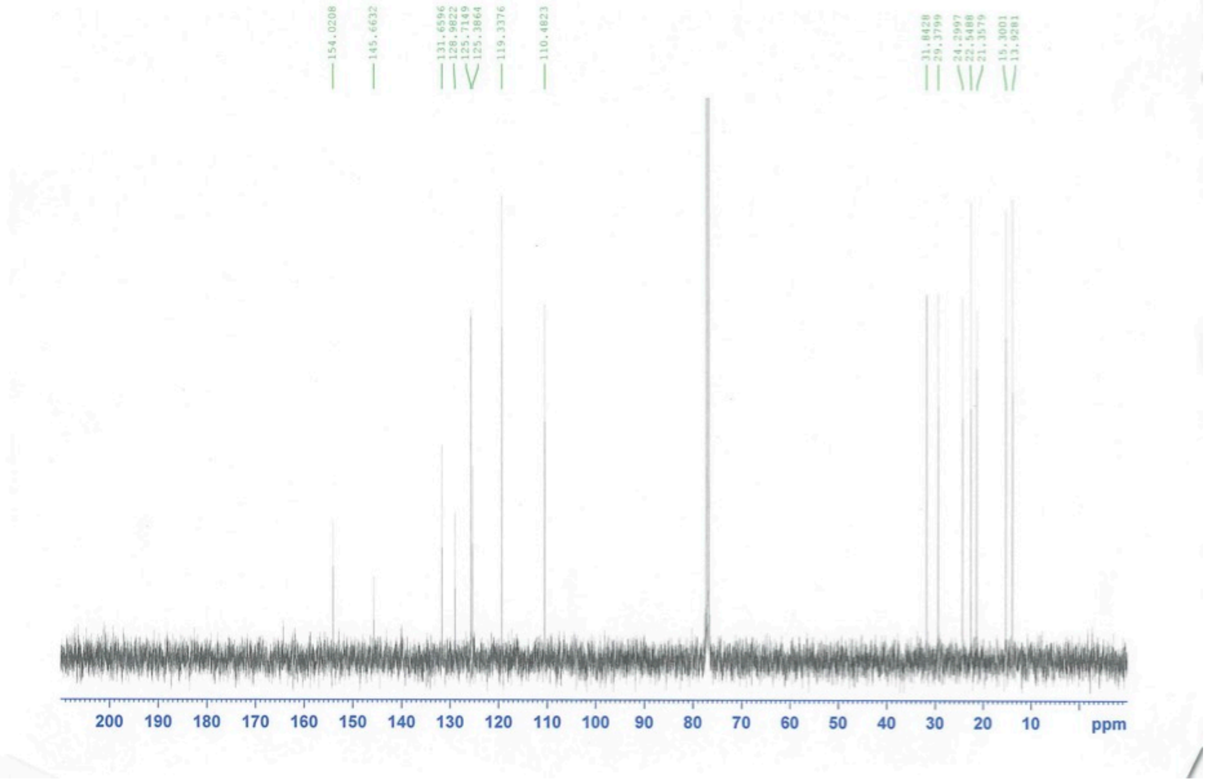

^{1}H NMR (400 MHz) and ^{13}C NMR (101 MHz) spectra of 3-butyl-2-(ethylthio)-5-methoxybenzo[*b*]furan (**3j**) ($CDCl_3$)

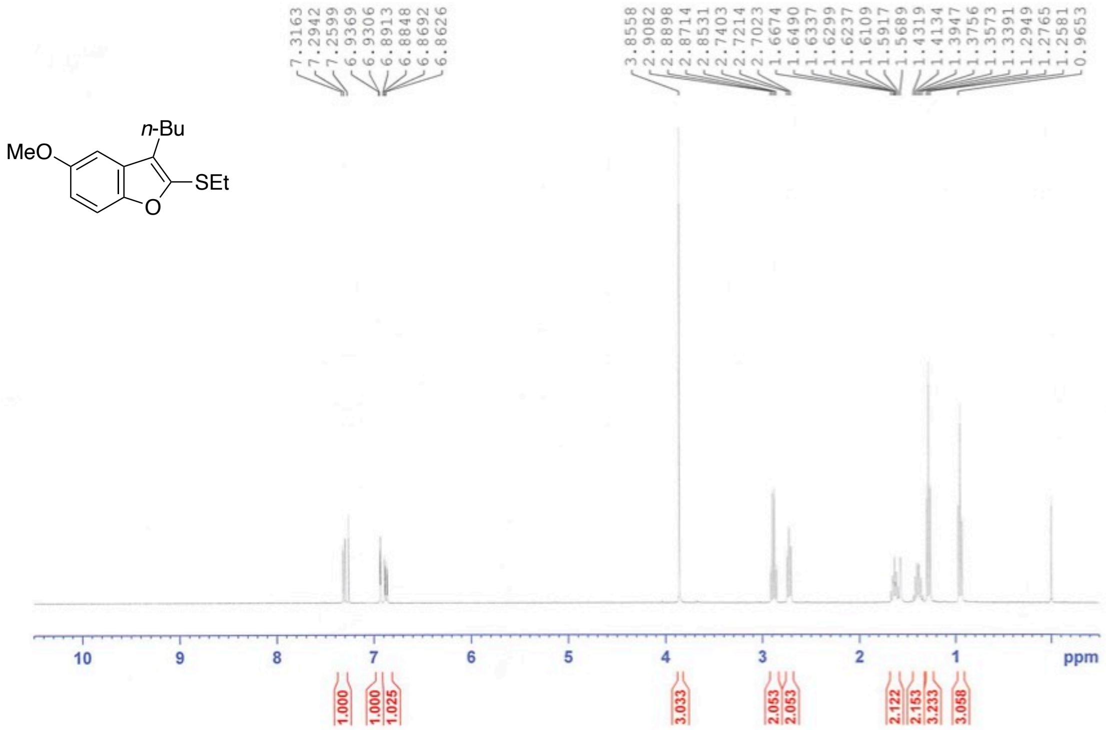

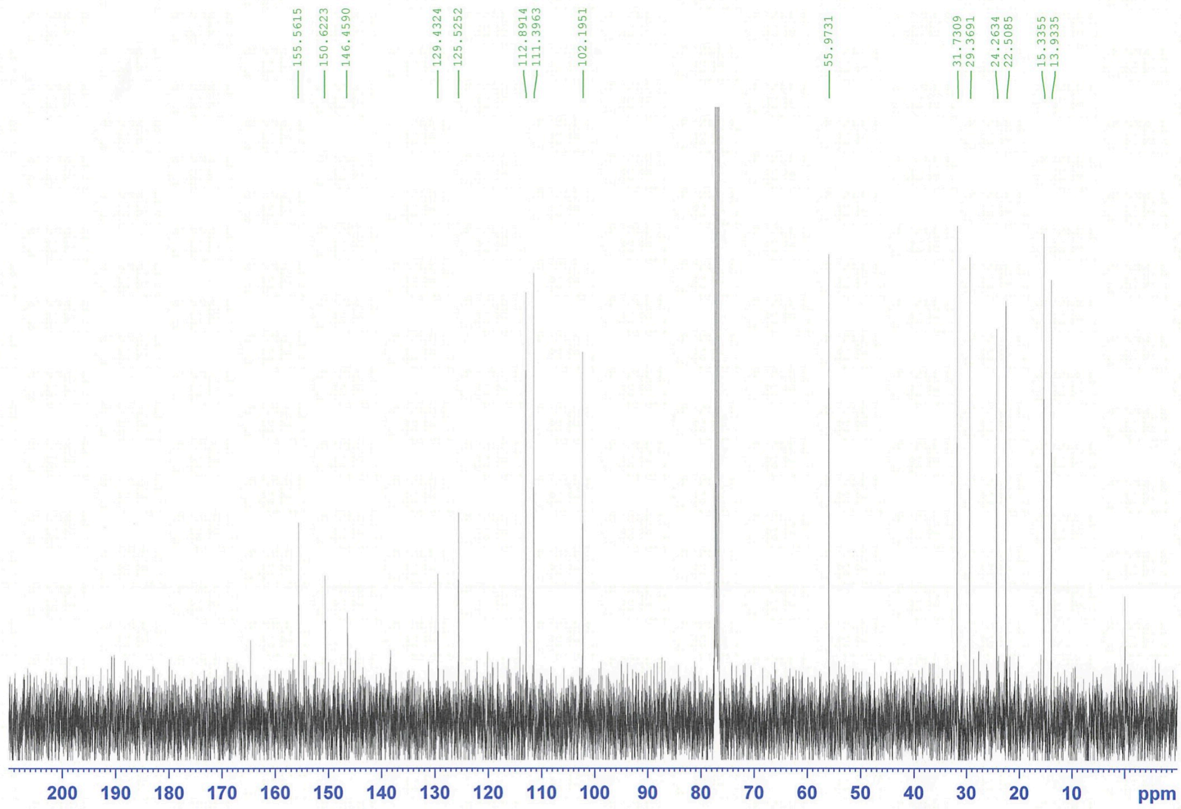

^{1}H NMR (400 MHz) and ^{13}C NMR (101 MHz) spectra of 5-bromo-3-butyl-2-(ethylthio)benzo[*b*]furan (**3k**) ($CDCl_3$)

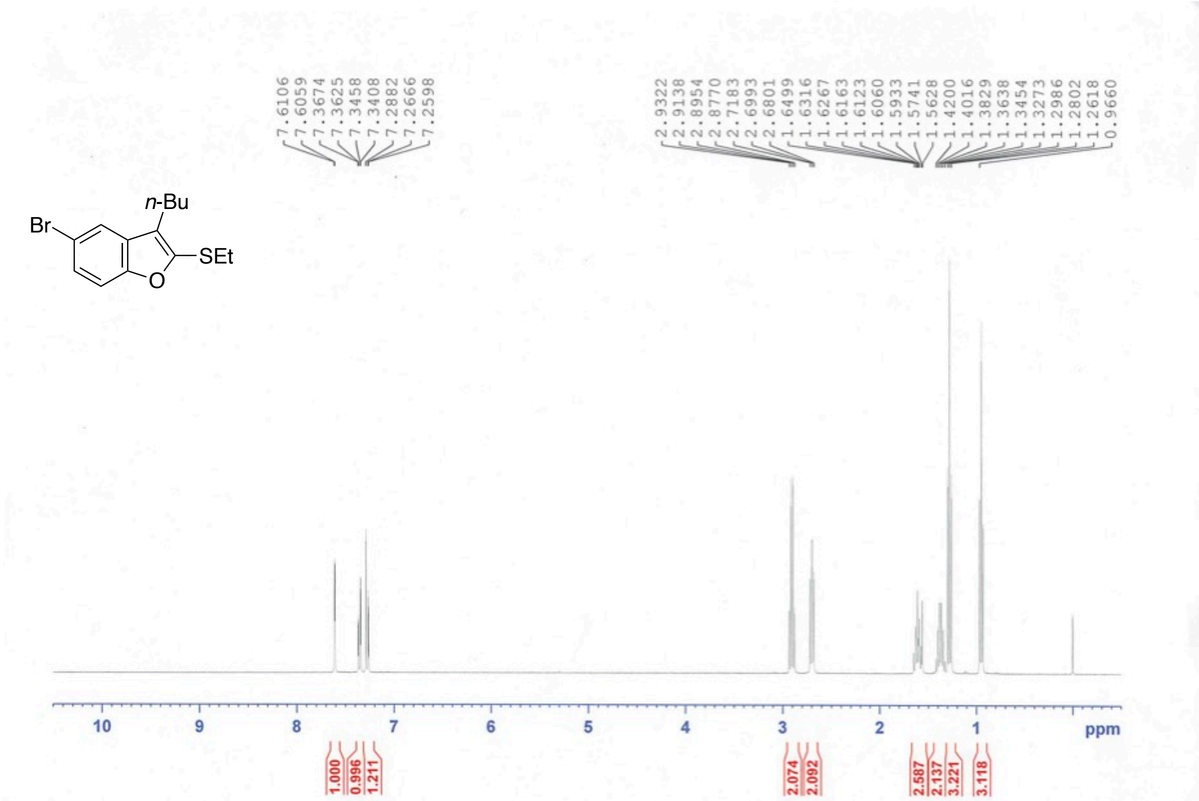

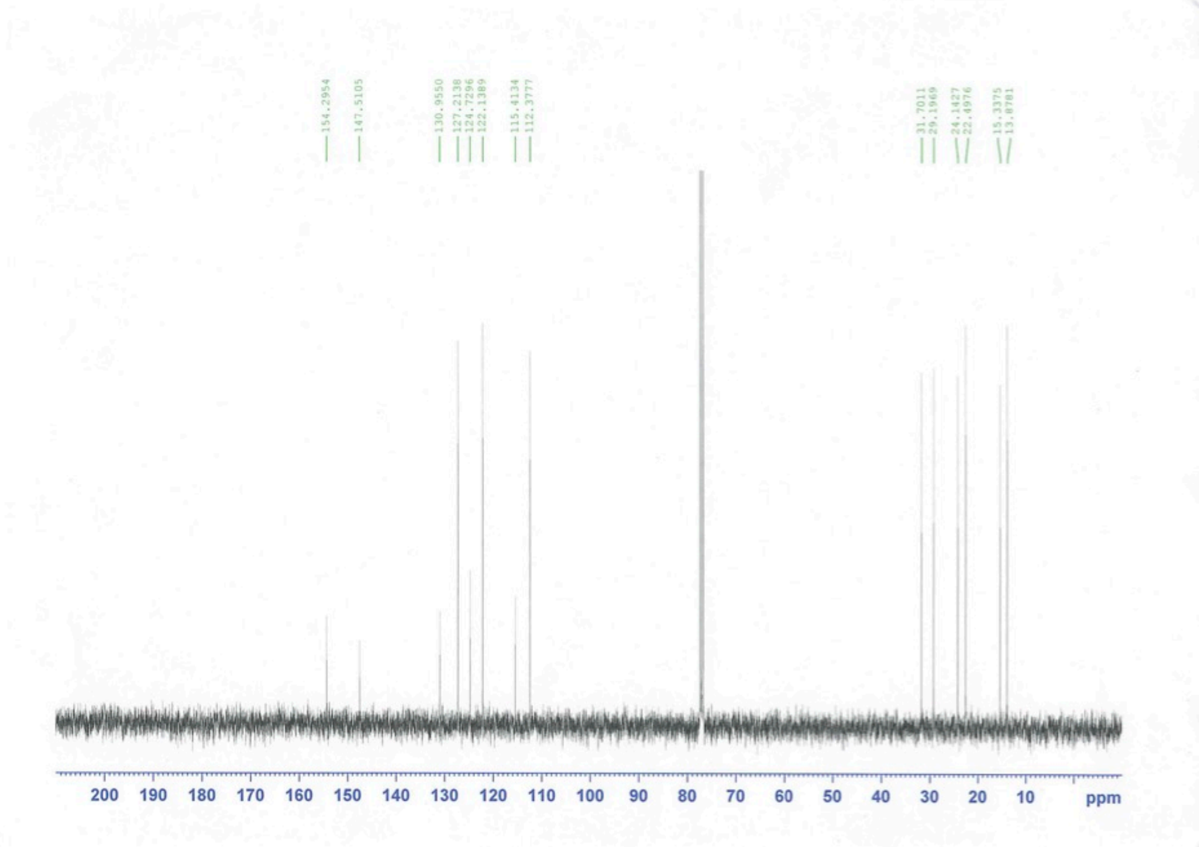

^{1}H NMR (400 MHz) and ^{13}C NMR (101 MHz) spectra of 3-butyl-5-chloro-2-(ethylthio)benzo[*b*]furan (**3l**) ($CDCl_3$)

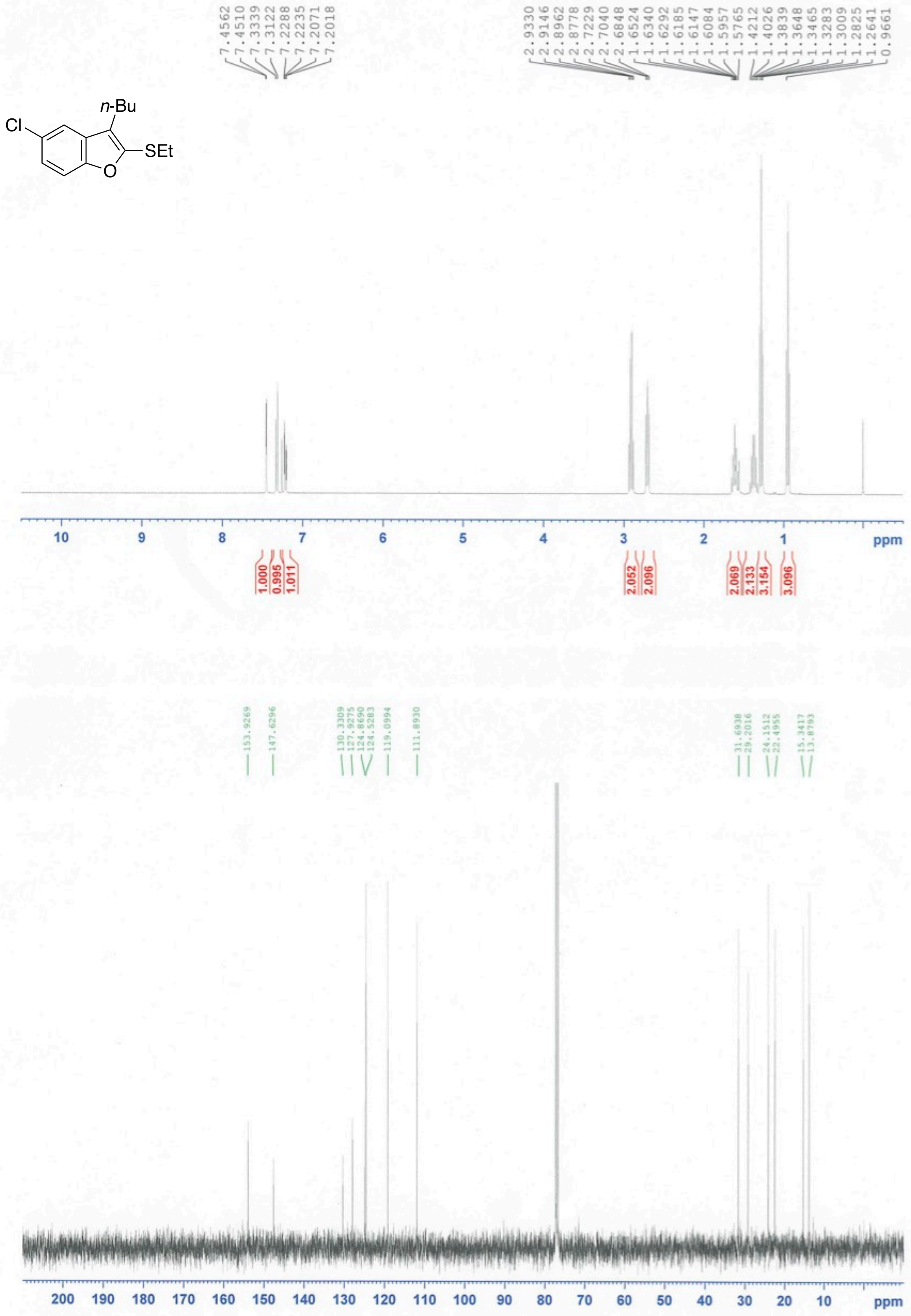

^{1}H NMR (400 MHz) and ^{13}C NMR (101 MHz) spectra of methyl 3-butyl-2-(ethylthio)benzo[*b*]furan-5-carboxylate (**3m**) ($CDCl_3$)

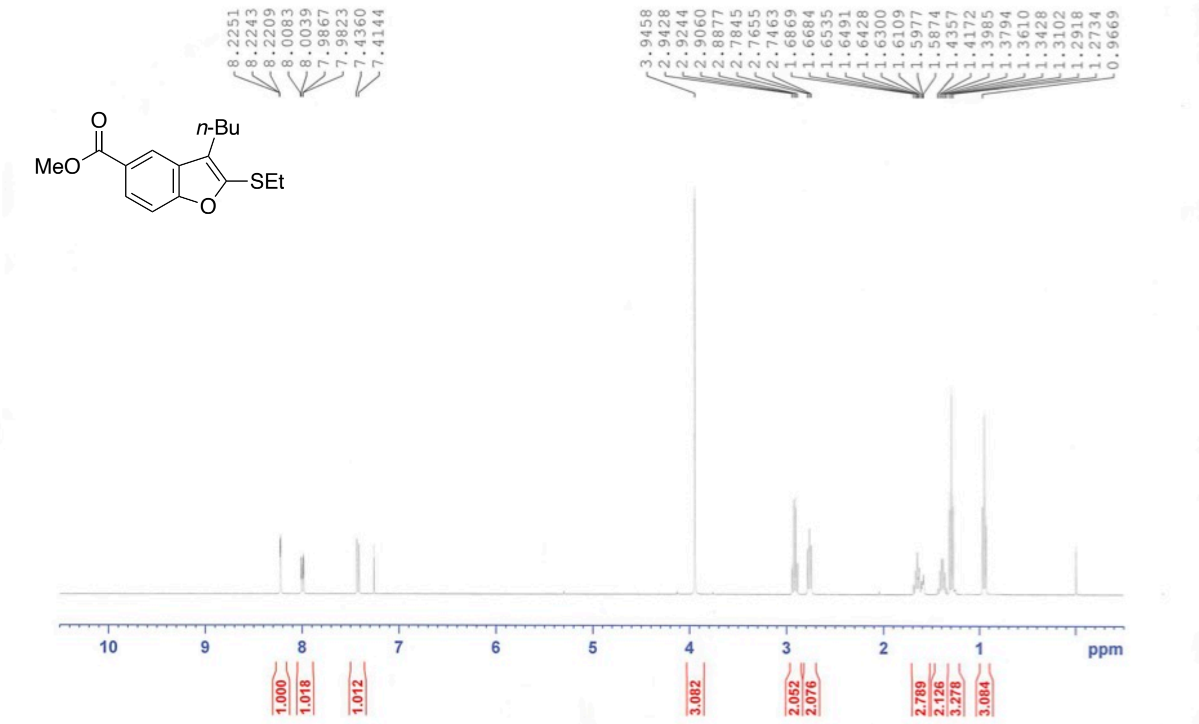

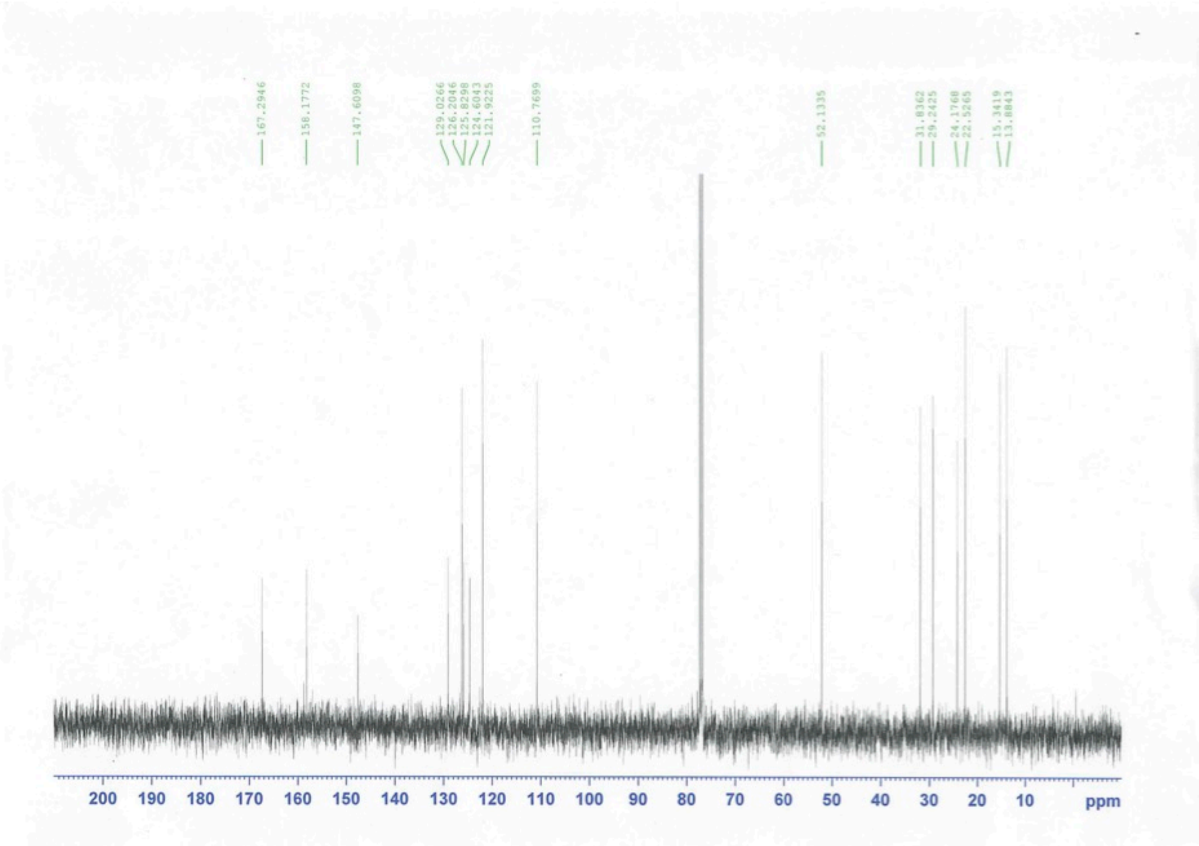

^1H NMR (400 MHz) and ^{13}C NMR (101 MHz) spectra of 2-(3-butyl-2-(ethylthio)benzo[*b*]furan-5-yl)-4,4,5,5-tetramethyl-1,3,2-dioxaborolane (**3n**) ($CDCl_3$)

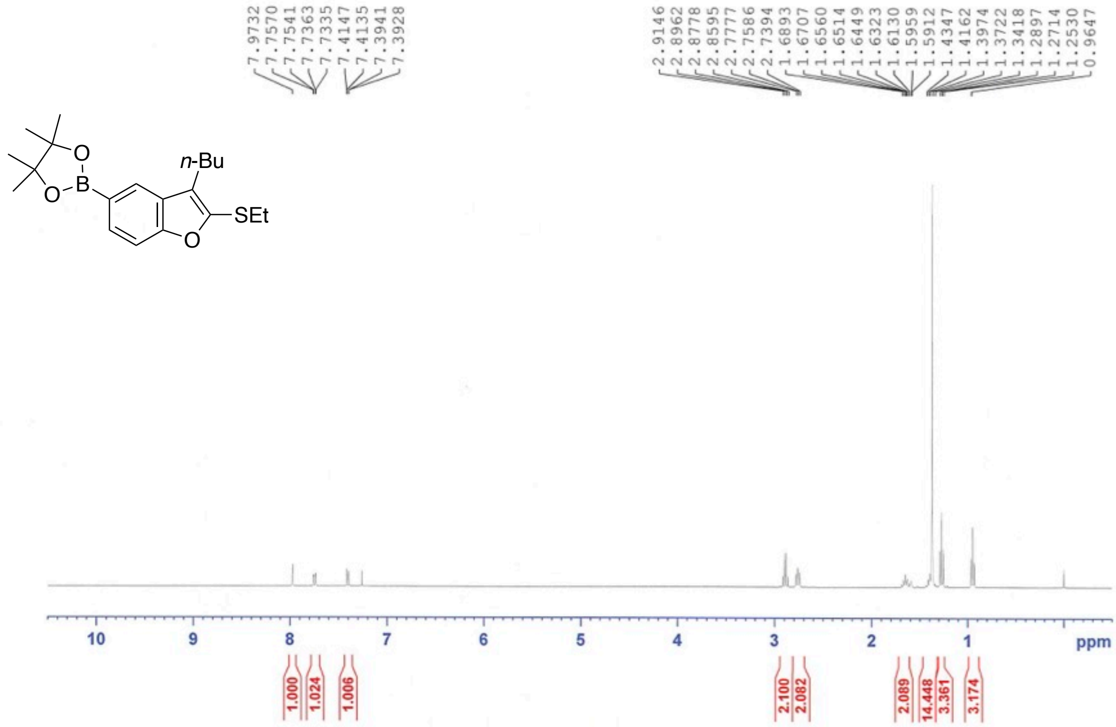

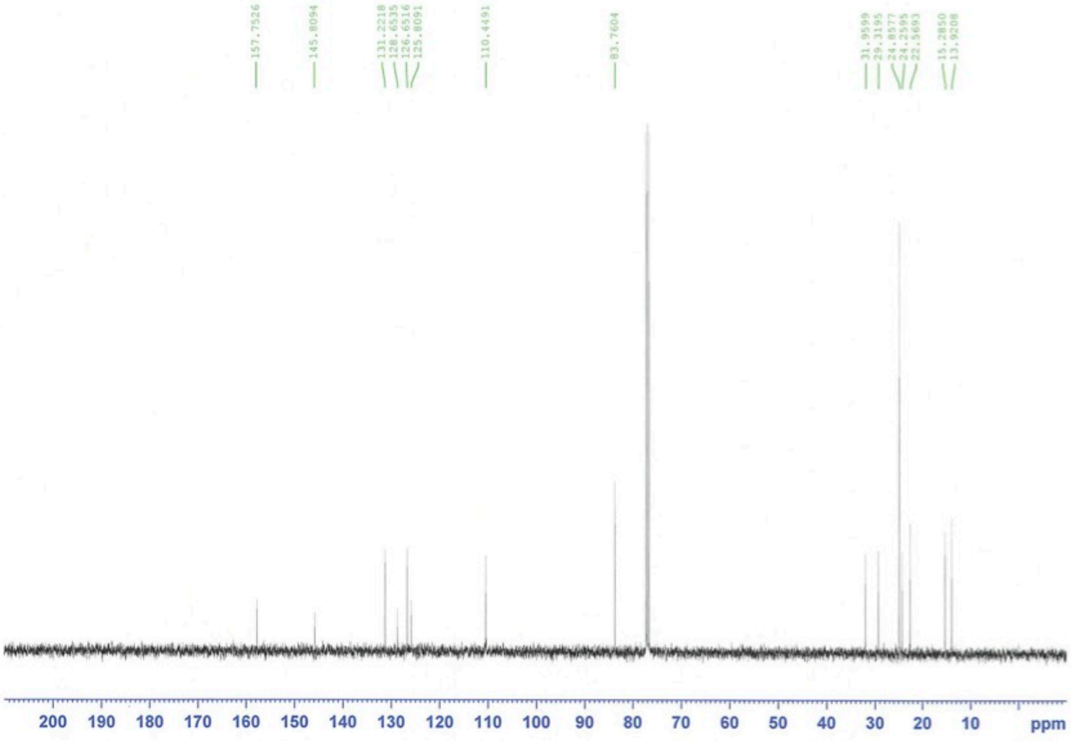

^{1}H NMR (400 MHz) and ^{13}C NMR (101 MHz) spectra of 3-butyl-2-(ethylthio)-7-(trimethylsilyl)benzo[*b*]furan-6-yl trifluoromethanesulfonate (**3o**) ($CDCl_3$)

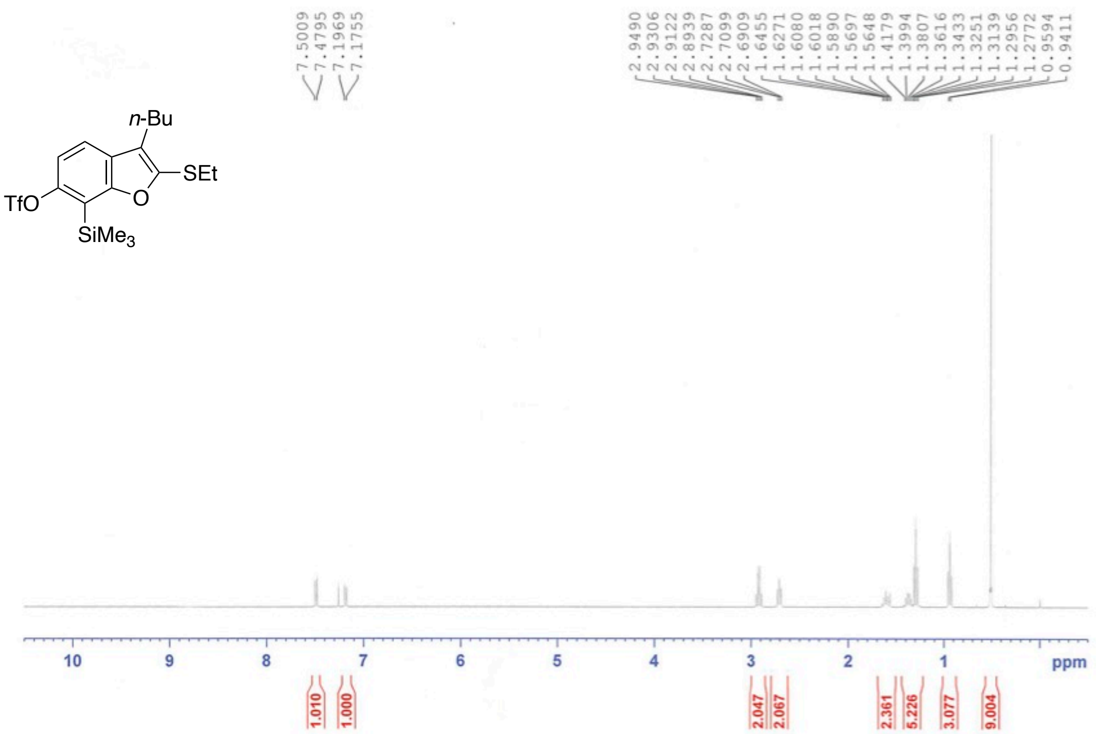

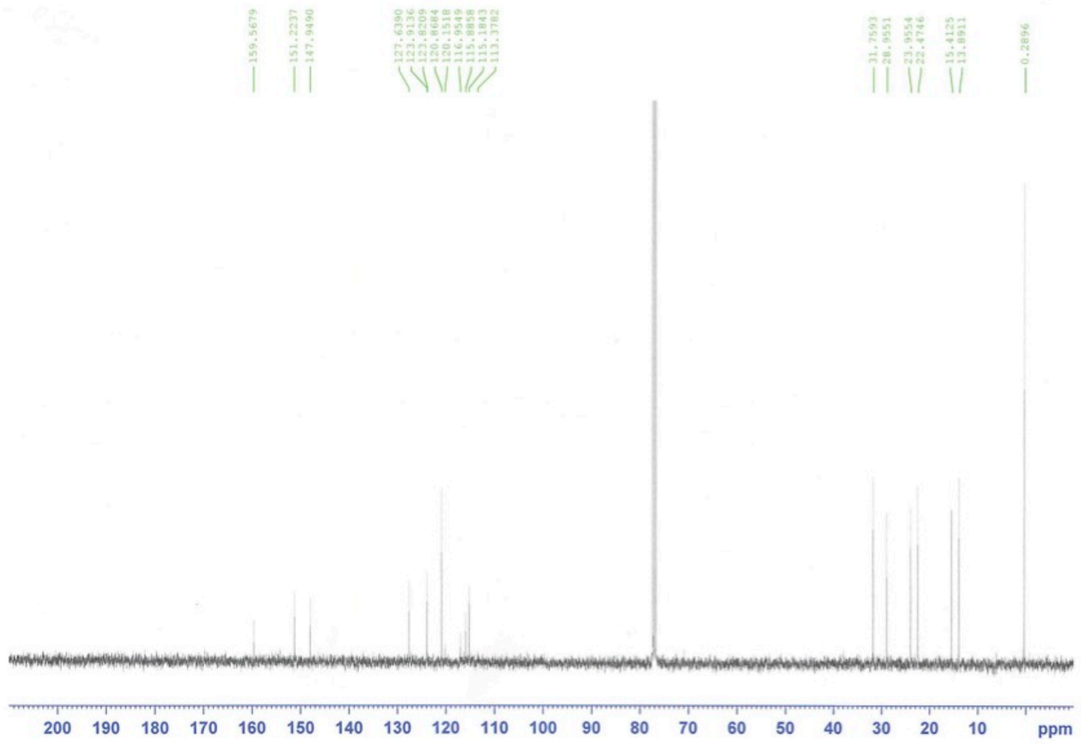

^{1}H NMR (400 MHz) and ^{13}C NMR (101 MHz) spectra of 3-butyl-2-(ethylthio)naphtho[1,2-*b*]furan (**3p**) ($CDCl_3$)

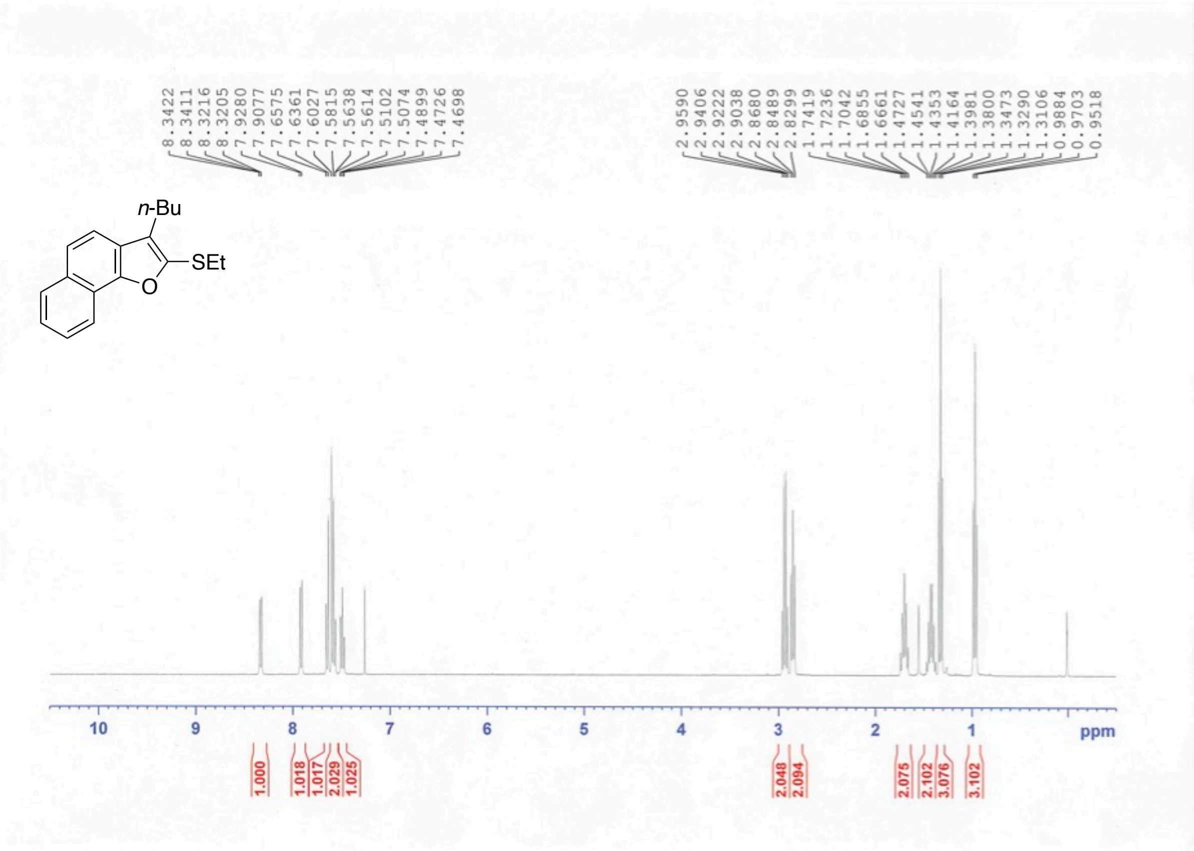

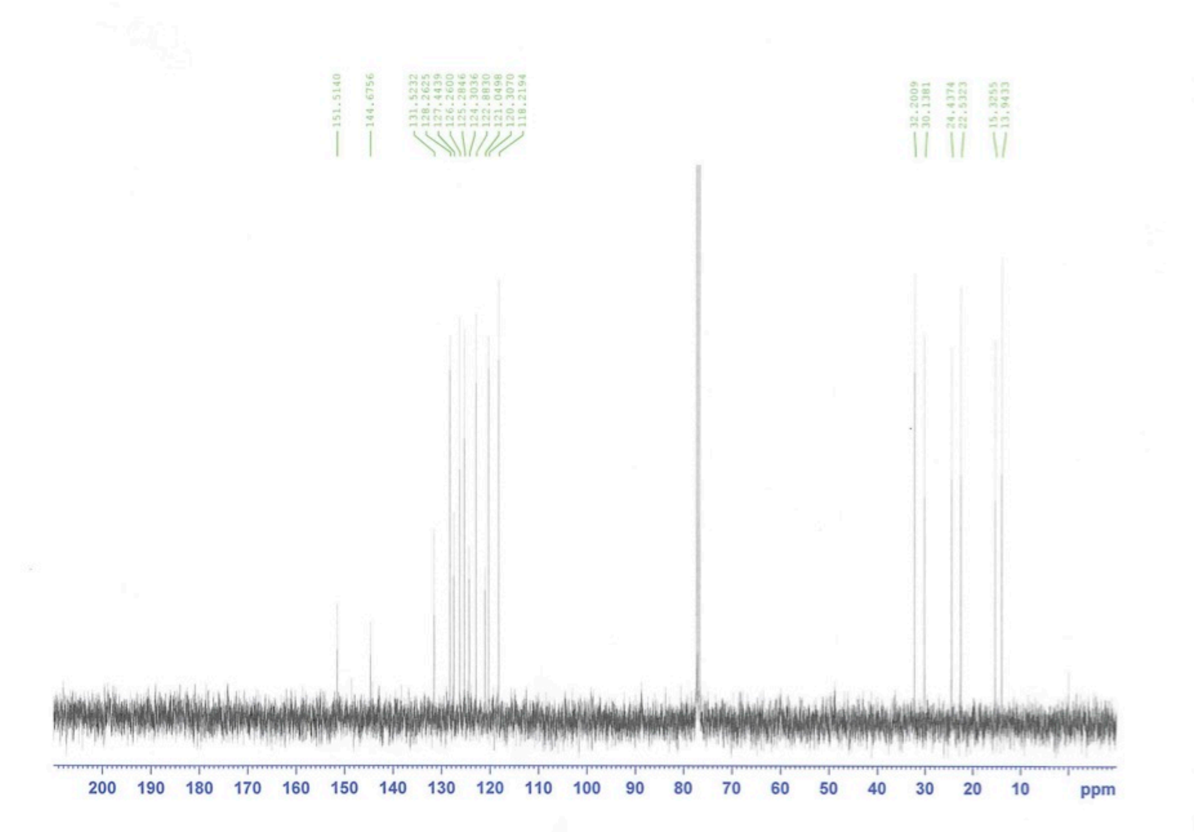

^{1}H NMR (400 MHz) and ^{13}C NMR (101 MHz) spectra of 3-butyl-2-(ethylthio)-6-methylbenzo[*b*]furan (**4a**) and 3-butyl-2-(ethylthio)-4-methylbenzo[*b*]furan (**5a**) ($CDCl_3$)

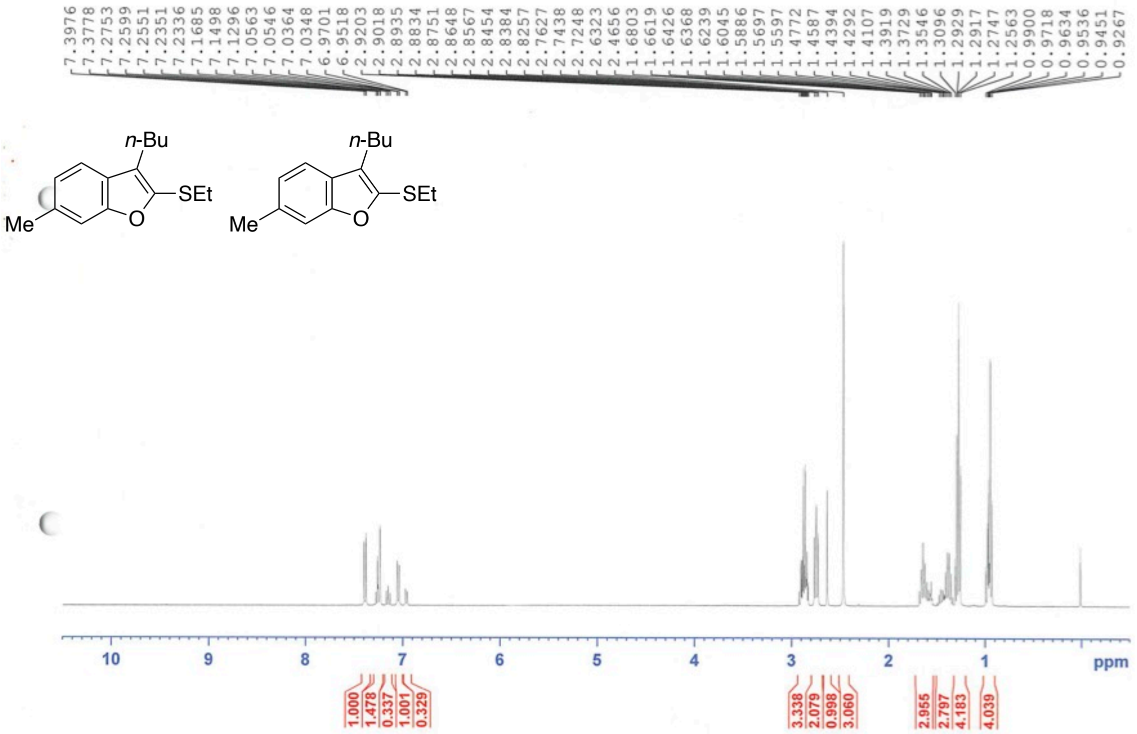

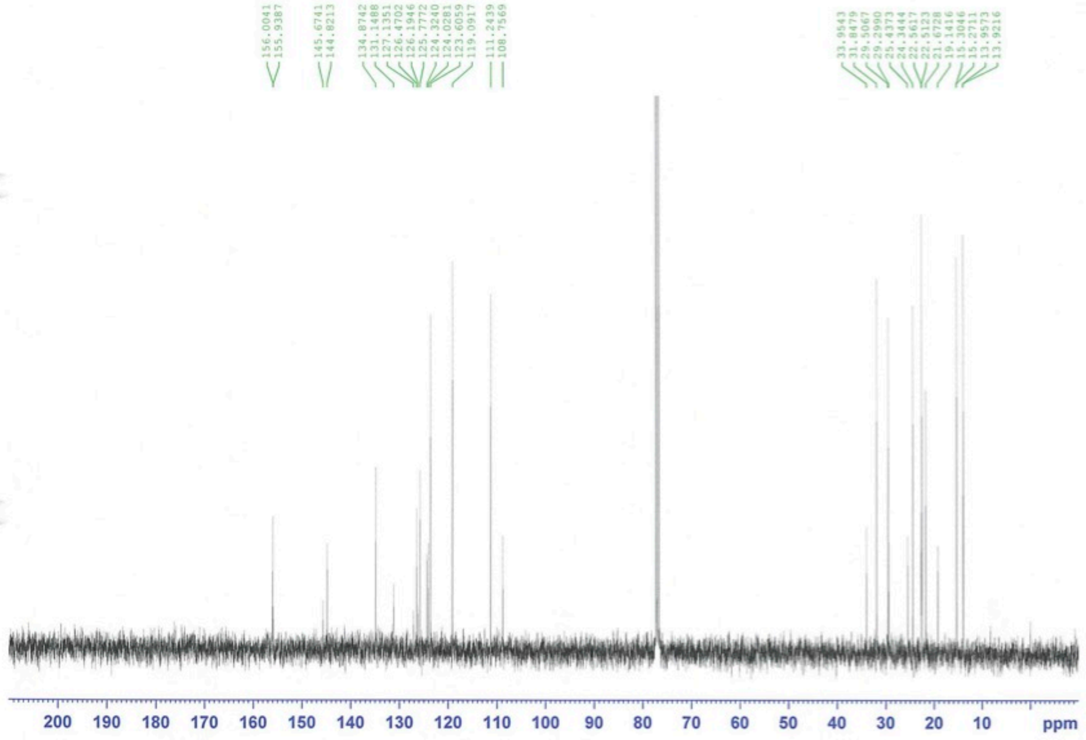

^{1}H NMR (400 MHz) and ^{13}C NMR (101 MHz) spectra of 3-butyl-2-(ethylthio)-6,7-dihydro-5*H*-indeno[5,6-*b*]furan (**4b**) and 1-butyl-2-(ethylthio)-7,8-dihydro-6*H*-indeno[5,4-*b*]furan (**5b**) ($CDCl_3$)

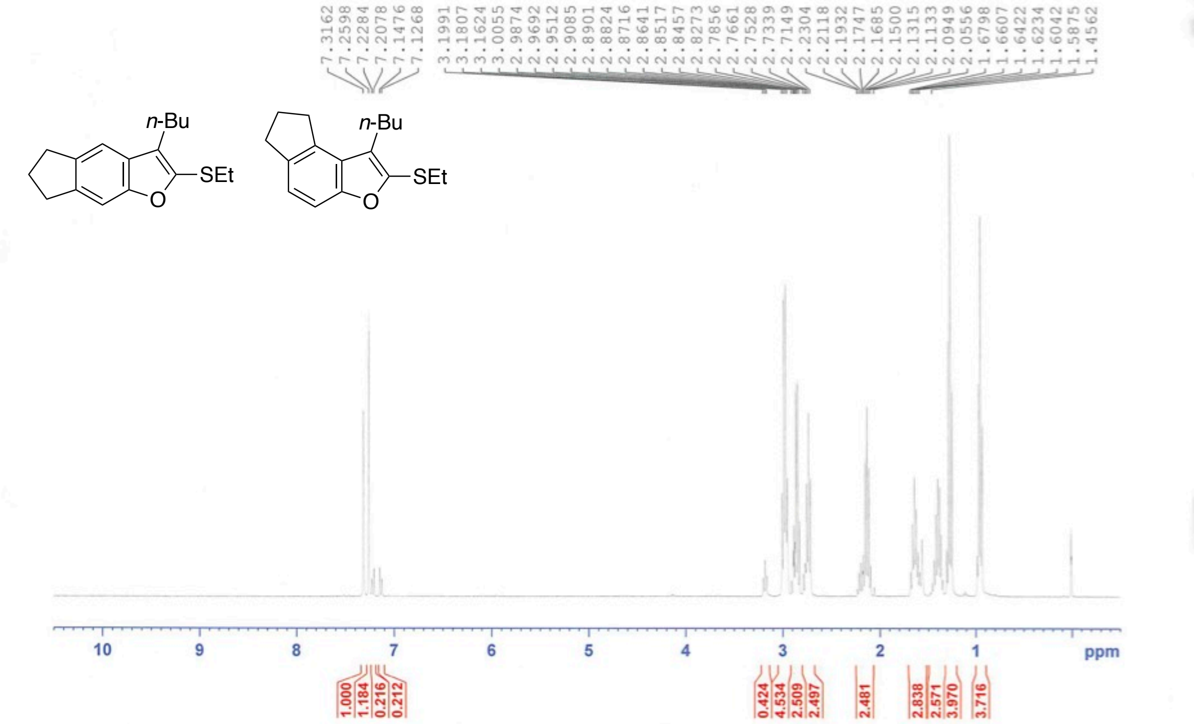

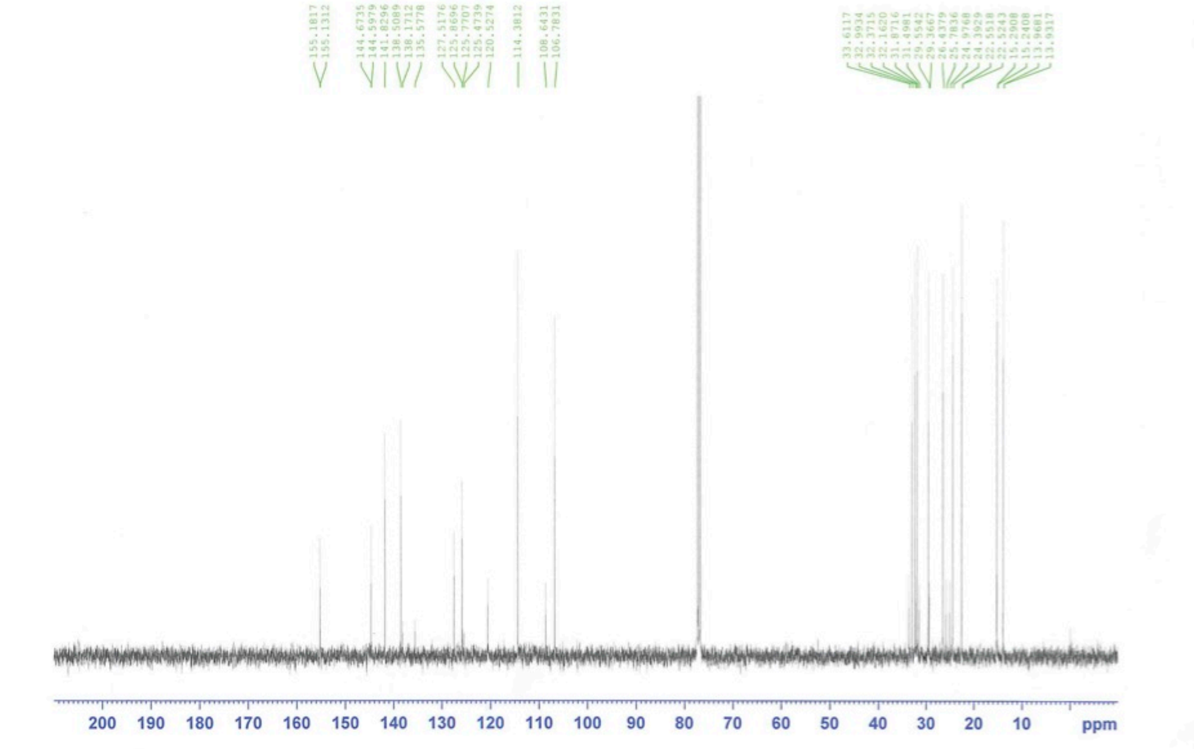

^{1}H NMR (400 MHz) and ^{13}C NMR (101 MHz) spectra of 3-butyl-2-(ethylthio)-5,6-dimethoxybenzo[*b*]furan (**4c**) ($CDCl_3$)

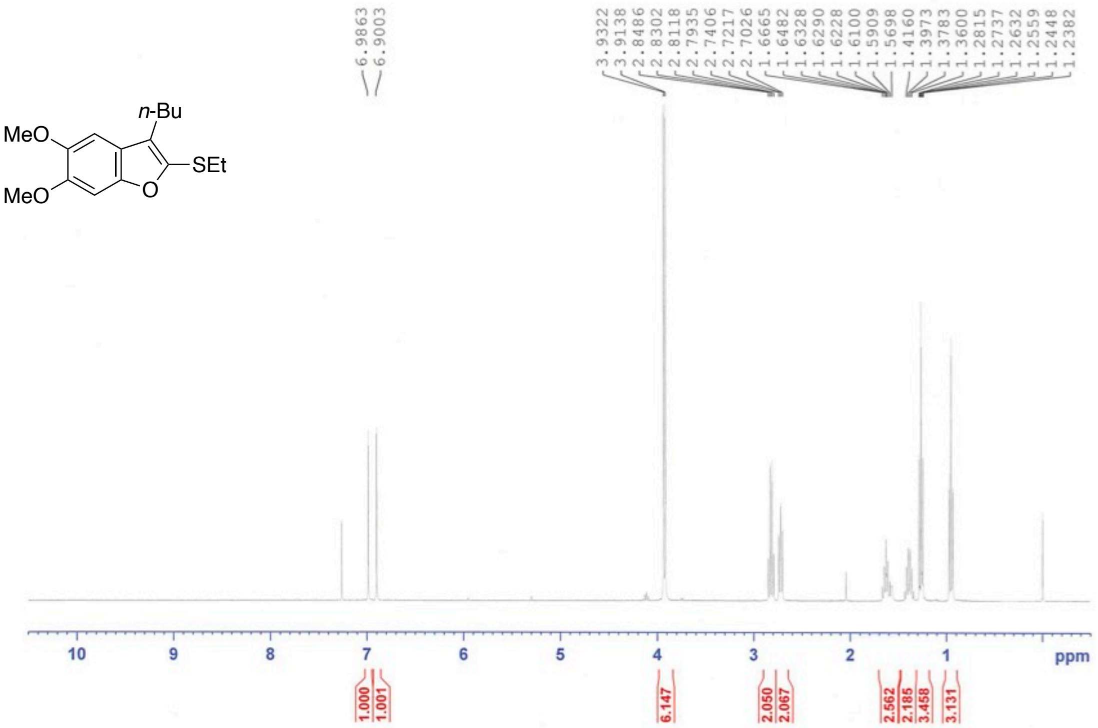

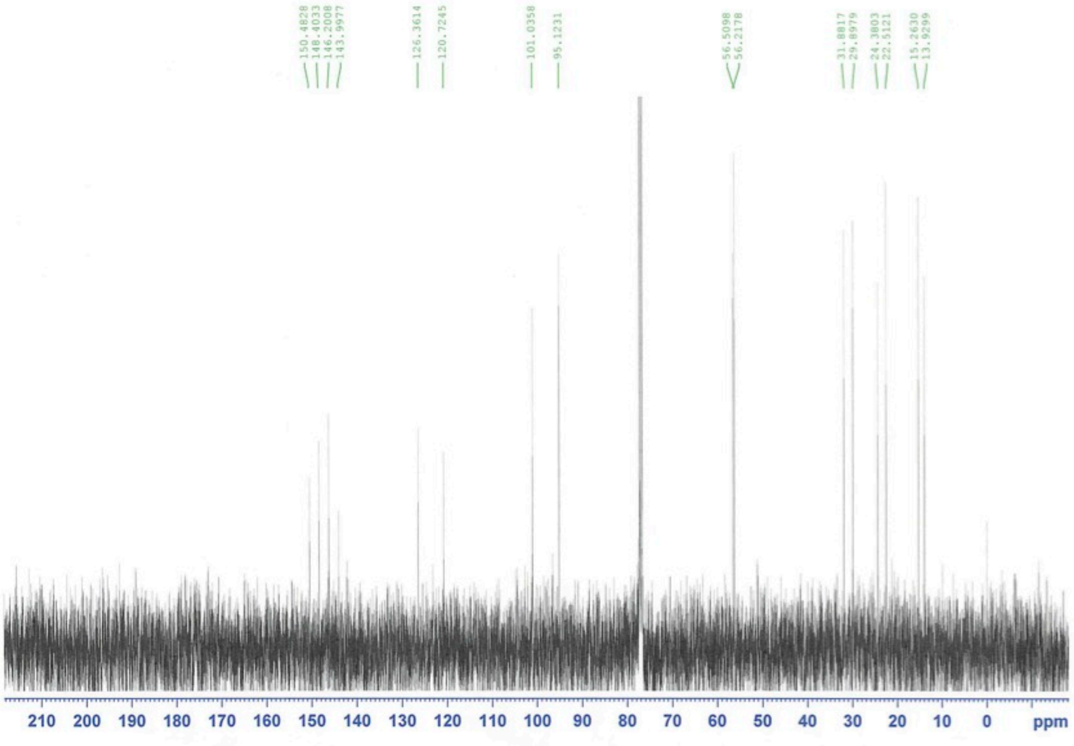

^{1}H NMR (400 MHz) and ^{13}C NMR (101 MHz) spectra of 1-butyl-2-(ethylthio)naphtho[2,1-*b*]furan (**5d**) ($CDCl_3$)

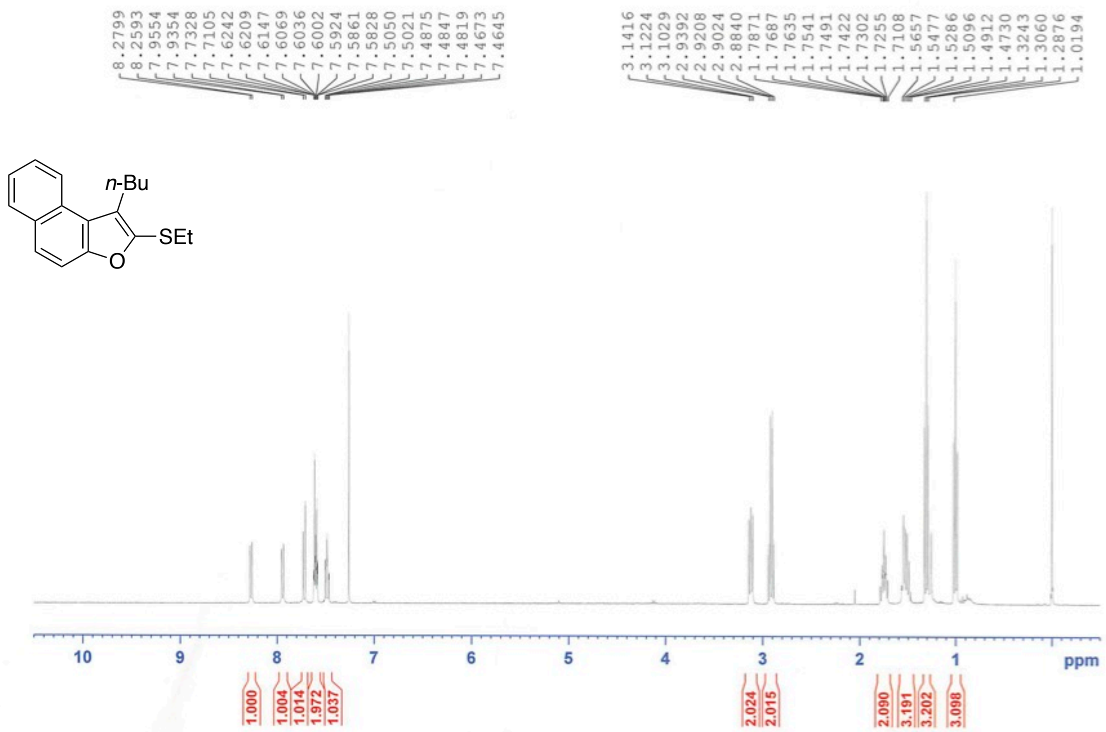

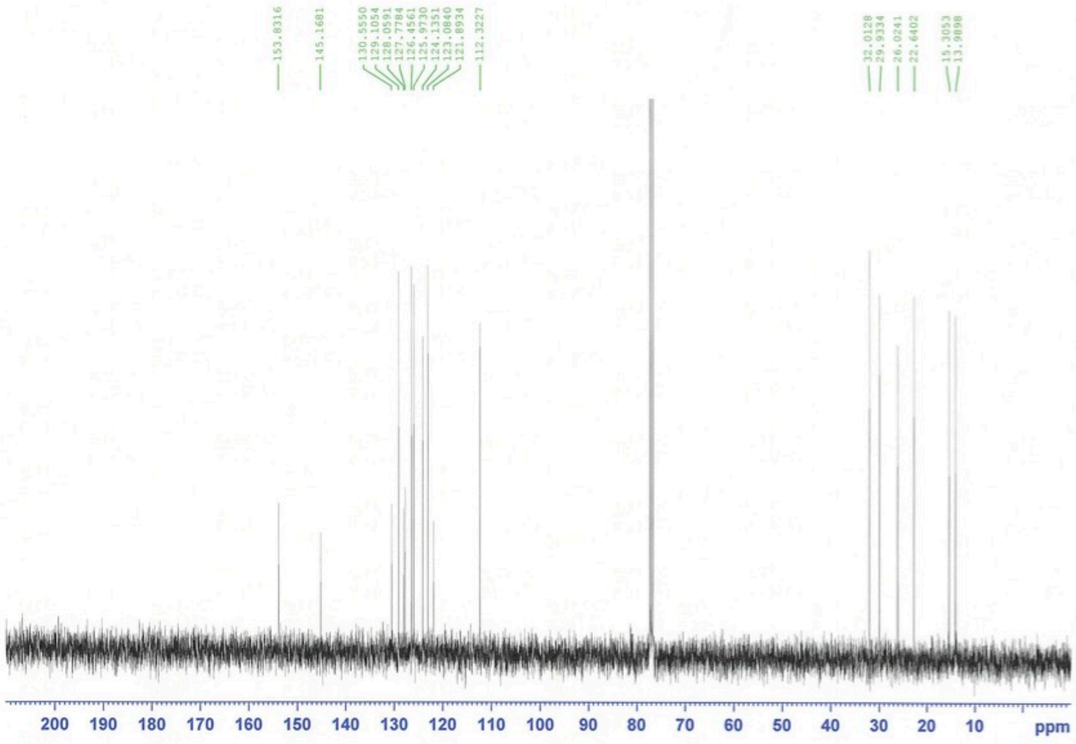

^{1}H NMR (400 MHz) and ^{13}C NMR (101 MHz) spectra of ((1-butyl-2-(ethylthio)naphtho[2,1-*b*]furan-8-yl)oxy)triisopropylsilane (**5e**) ($CDCl_3$)

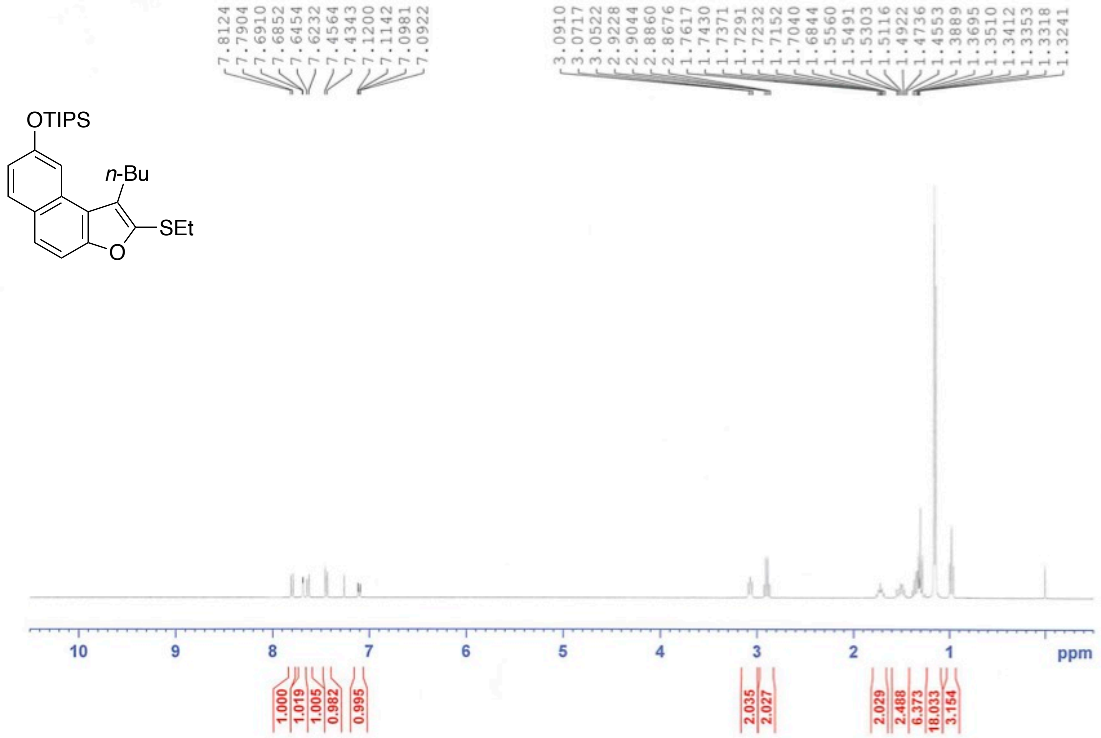

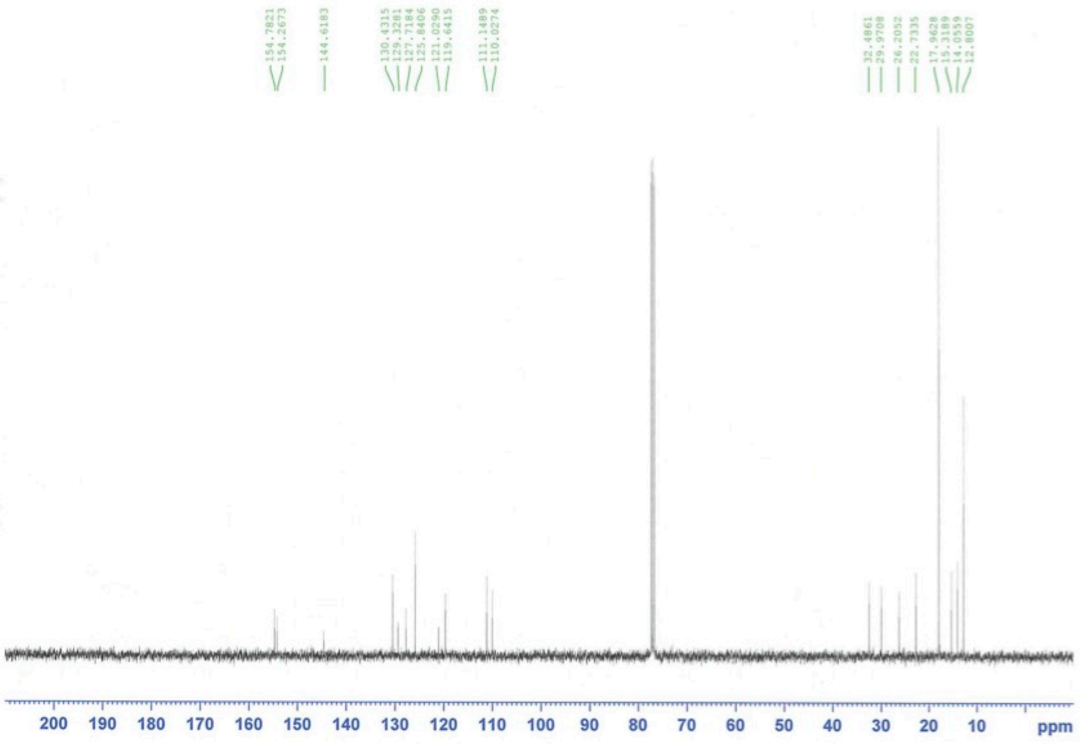

^{1}H NMR (400 MHz) and ^{13}C NMR (101 MHz) spectra of 2-ethylthio-3-butylbenzofuro[6,5-*b*]benzofuran (**4f**) ($CDCl_3$)

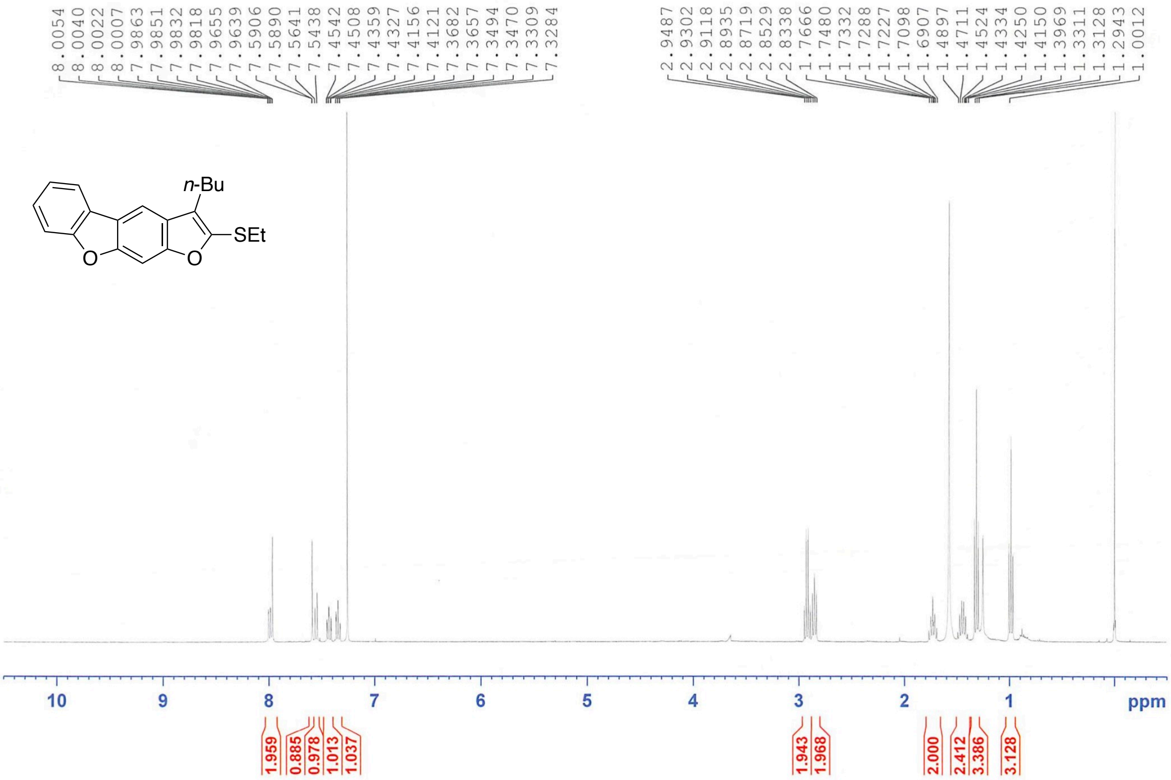

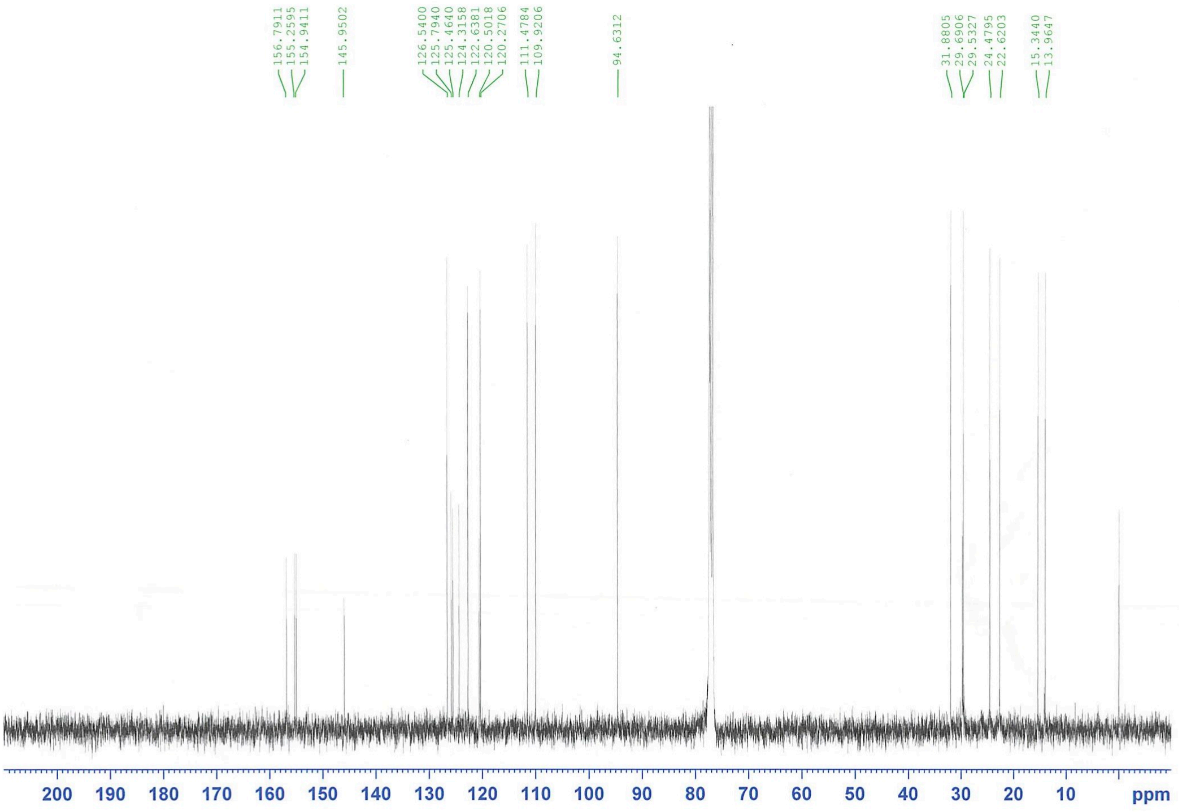

^{1}H NMR (400 MHz) and ^{13}C NMR (101 MHz) spectra of 2-ethylthio-3-butylbenzo[*b*]furo[2,3-*g*]benzofuran (**5f**) ($CDCl_3$)

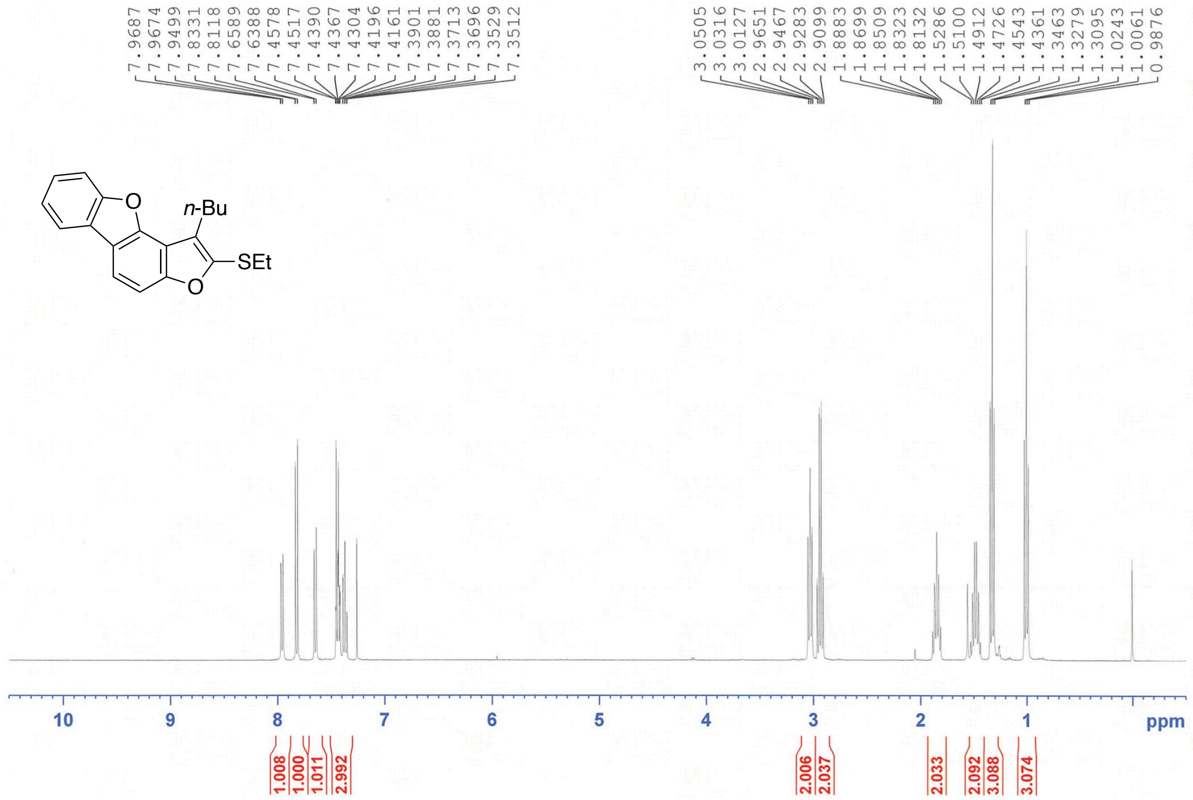

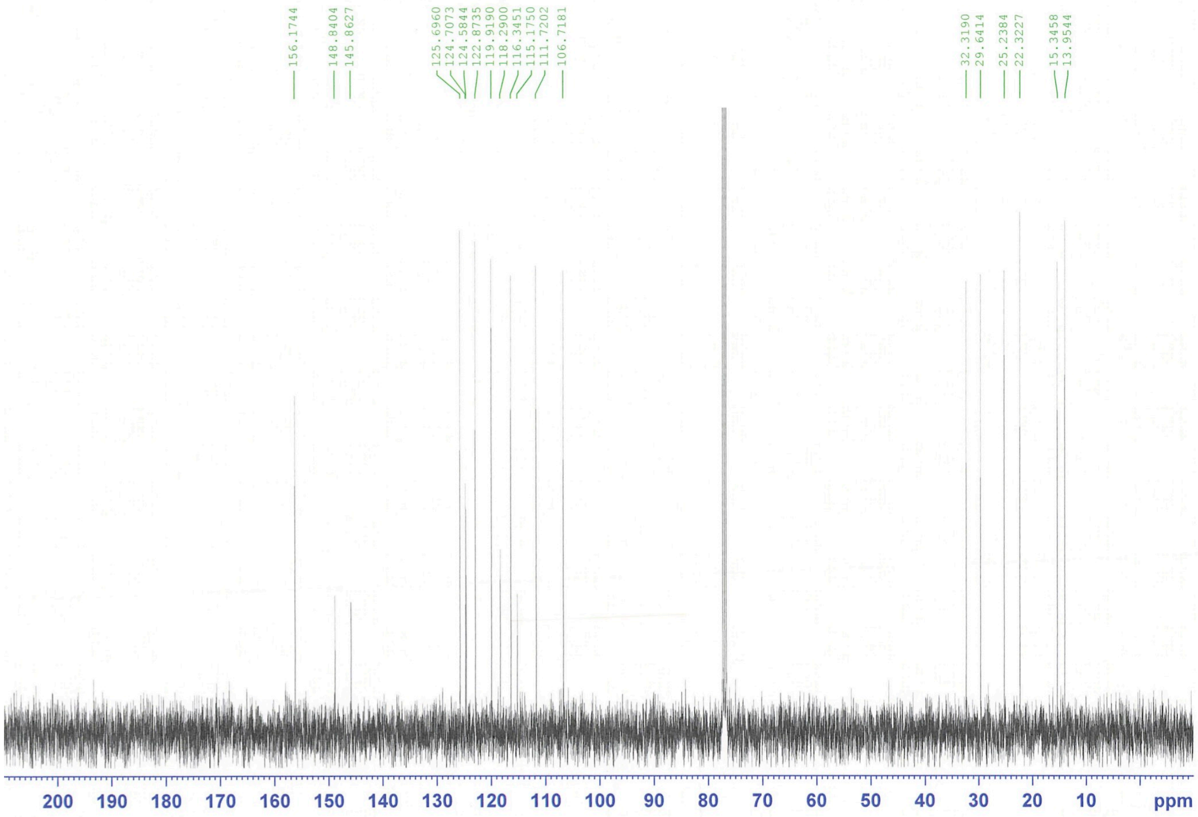

^{1}H NMR (400 MHz) and ^{13}C NMR (101 MHz) spectra of 3-butyl-2-(methylthio)benzo[*b*]furan (**3q**) ($CDCl_3$)

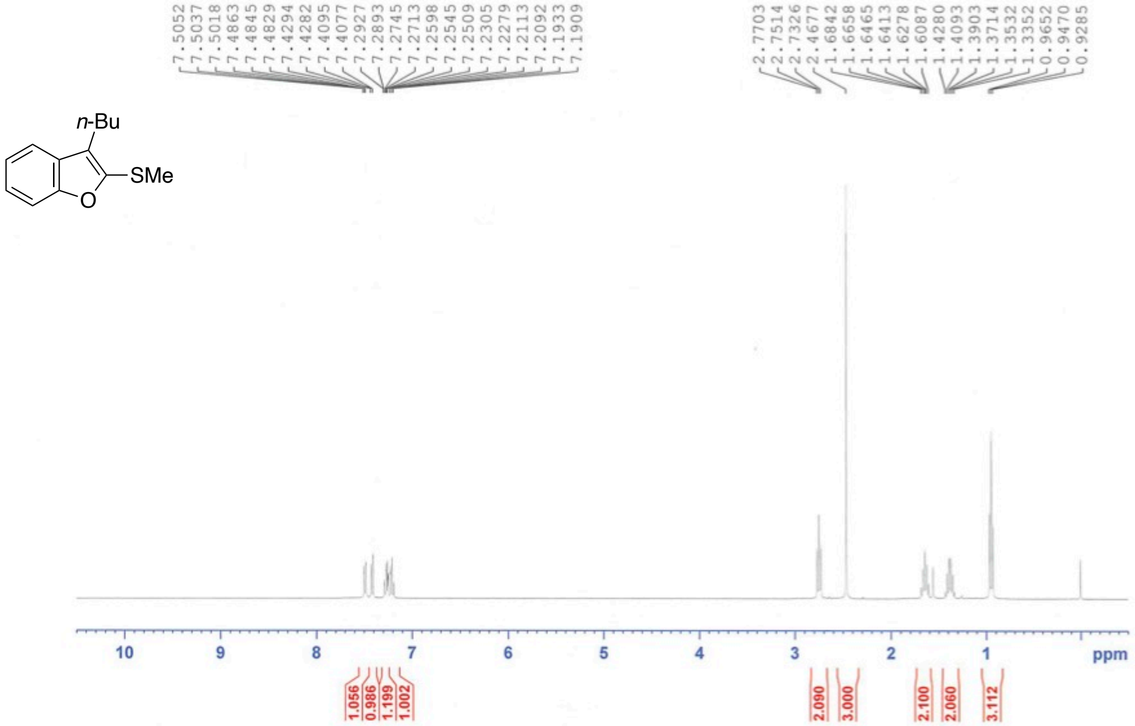

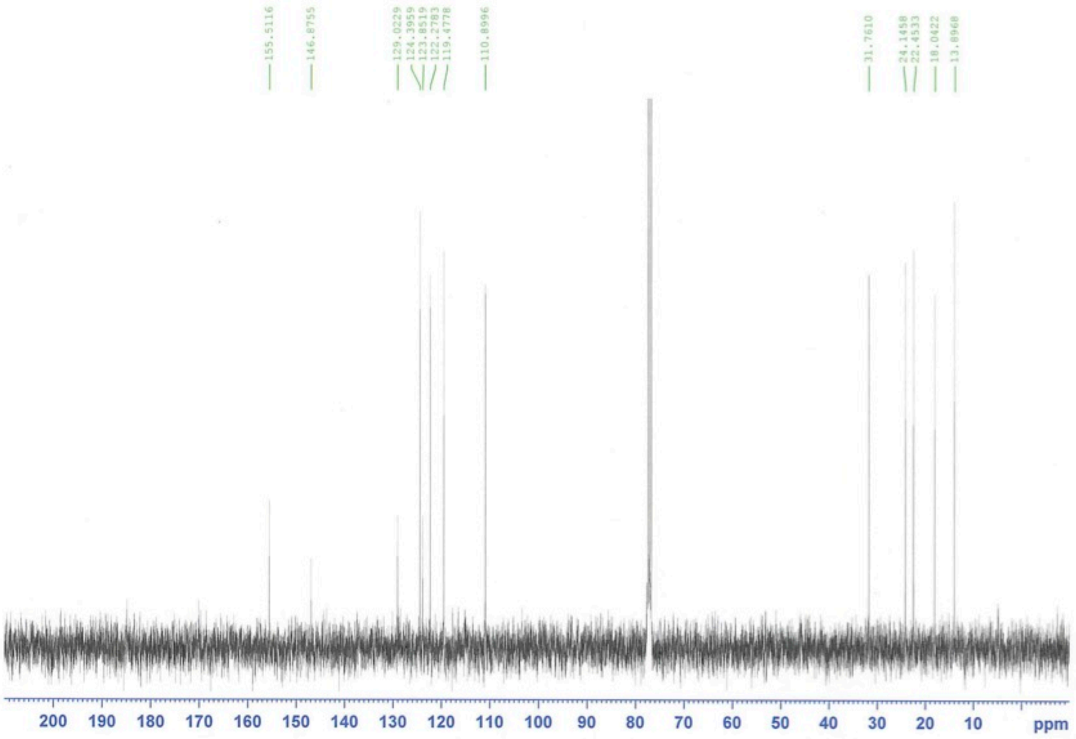

^{1}H NMR (400 MHz) and ^{13}C NMR (101 MHz) spectra of 2-(benzylthio)-3-butylbenzo[*b*]furan (**3r**) ($CDCl_3$)

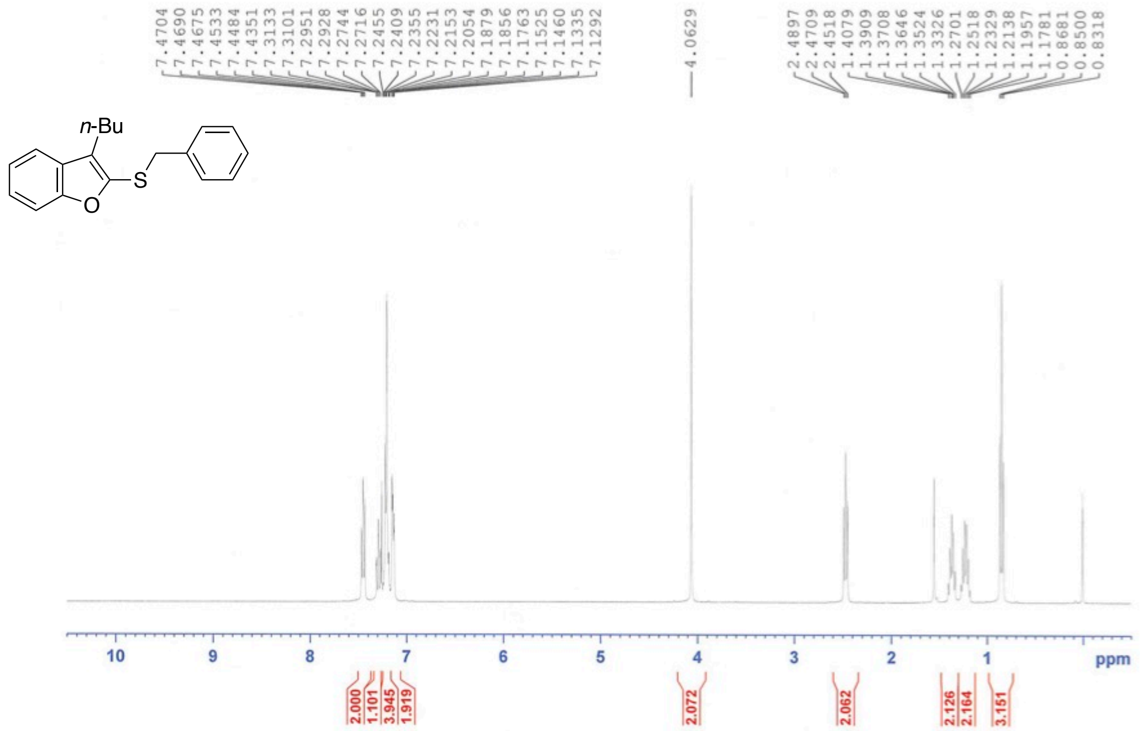

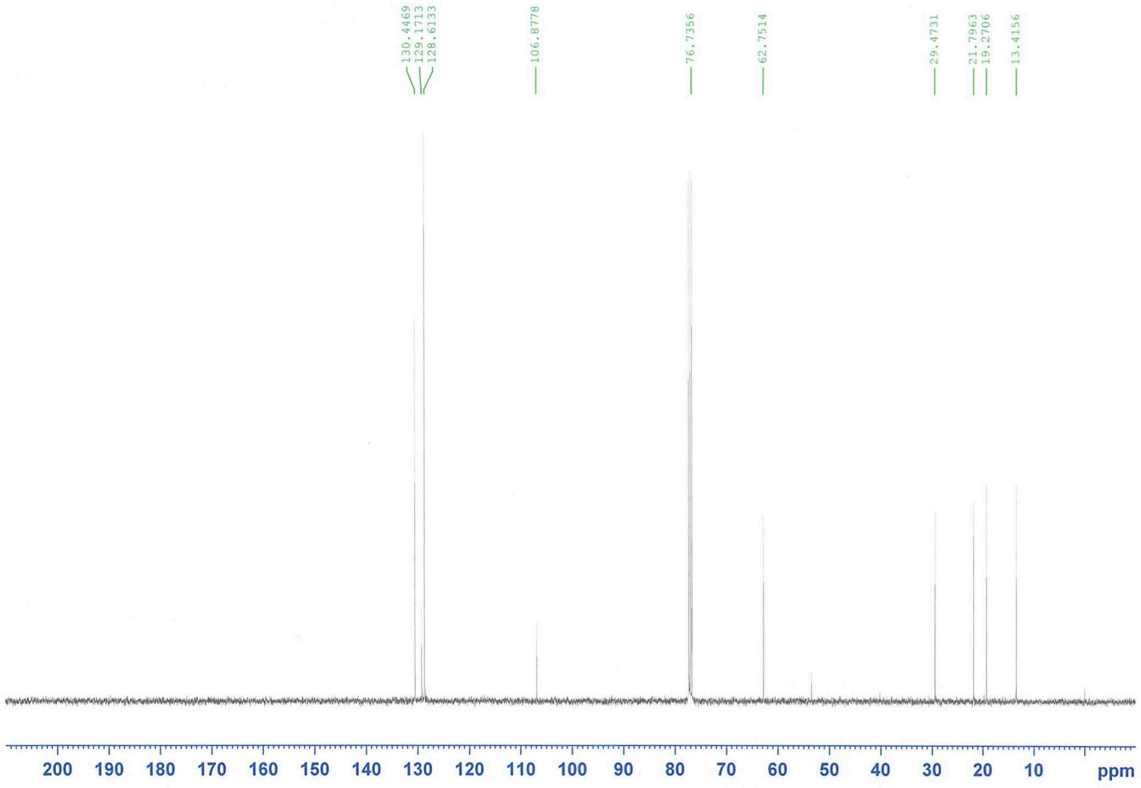

^1H NMR (400 MHz) and ^{13}C NMR (101 MHz) spectra of 3-butyl-2-((6-chlorohexyl)thio)benzo[*b*]furan (**3s**) ($CDCl_3$)

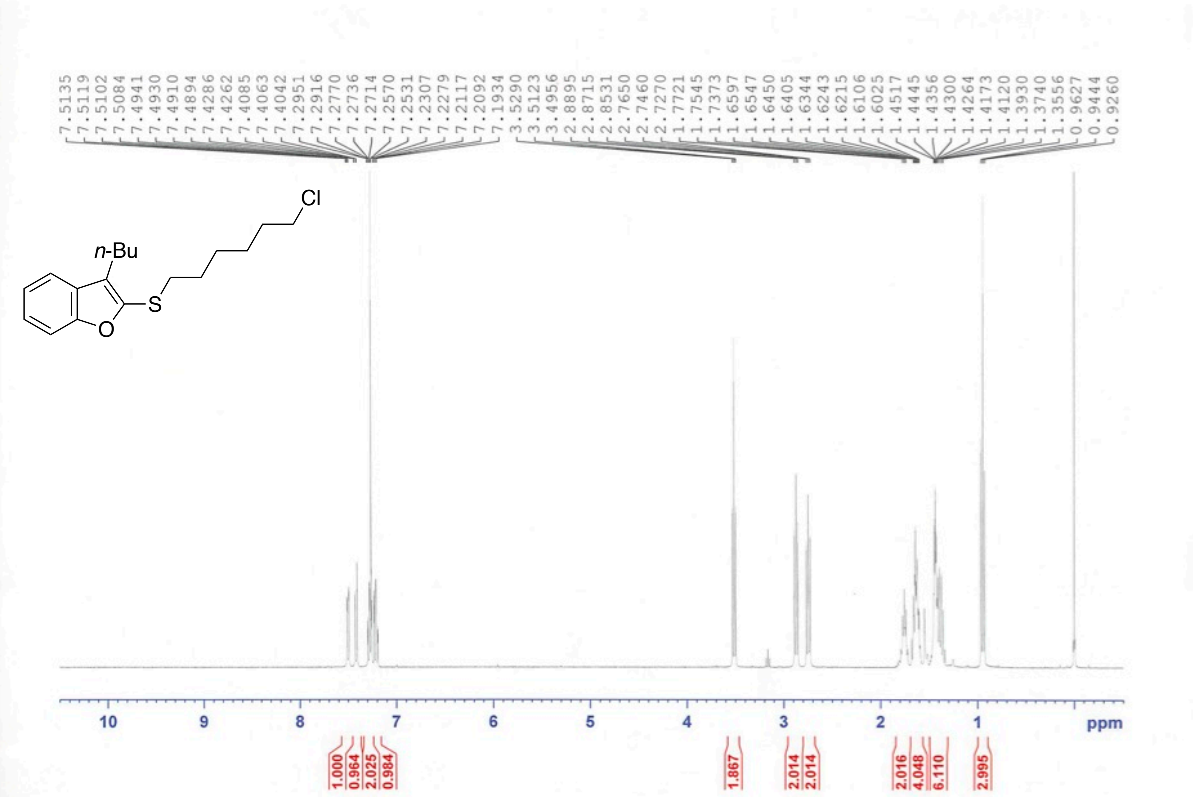

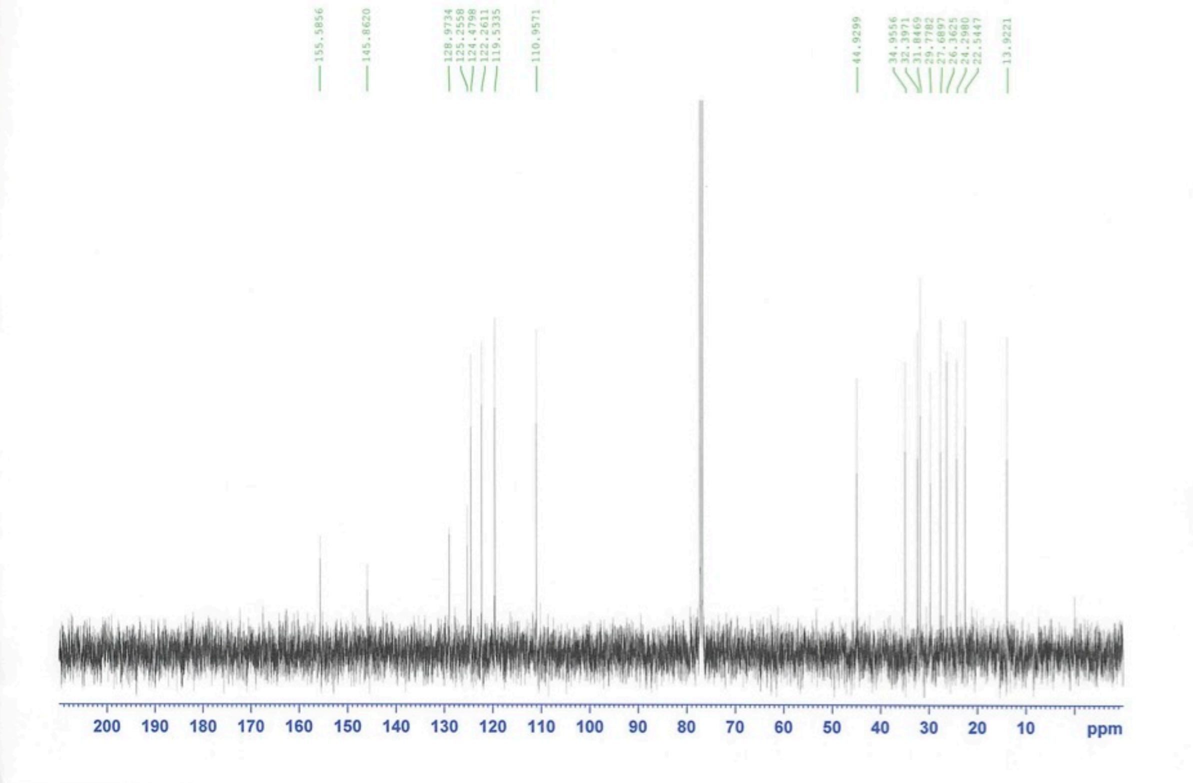

^{1}H NMR (400 MHz) and ^{13}C NMR (101 MHz) spectra of 3-butyl-2-(ethylsulfinyl)benzo[*b*]furan (**7**) ($CDCl_3$)

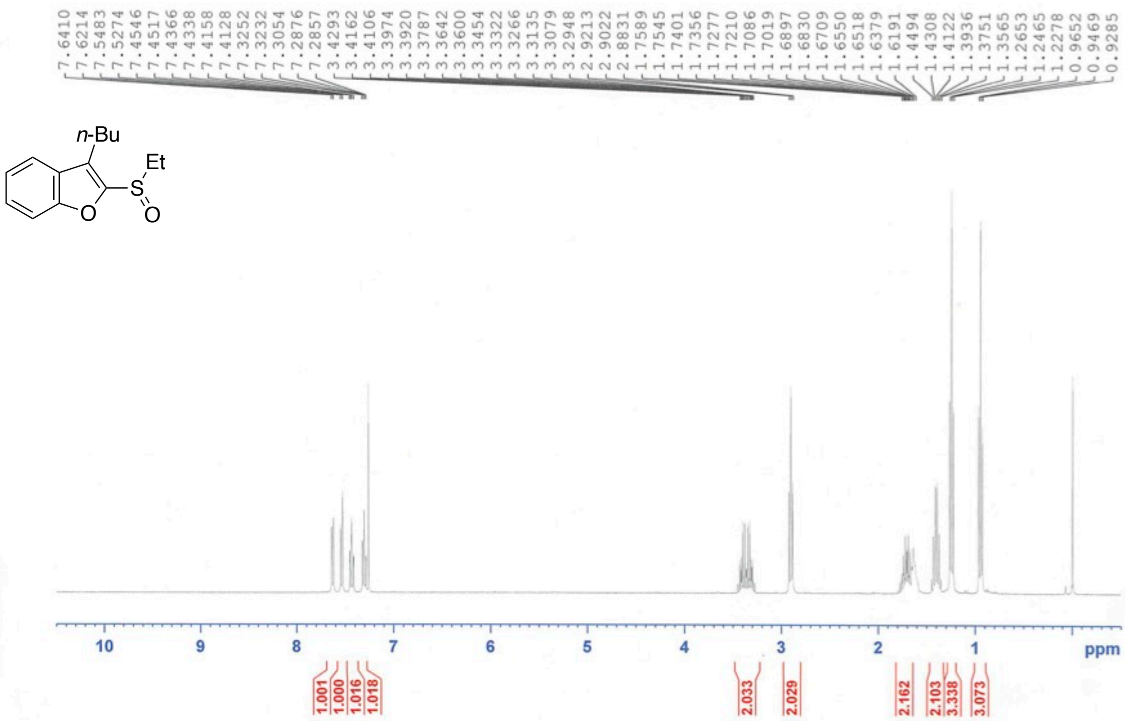

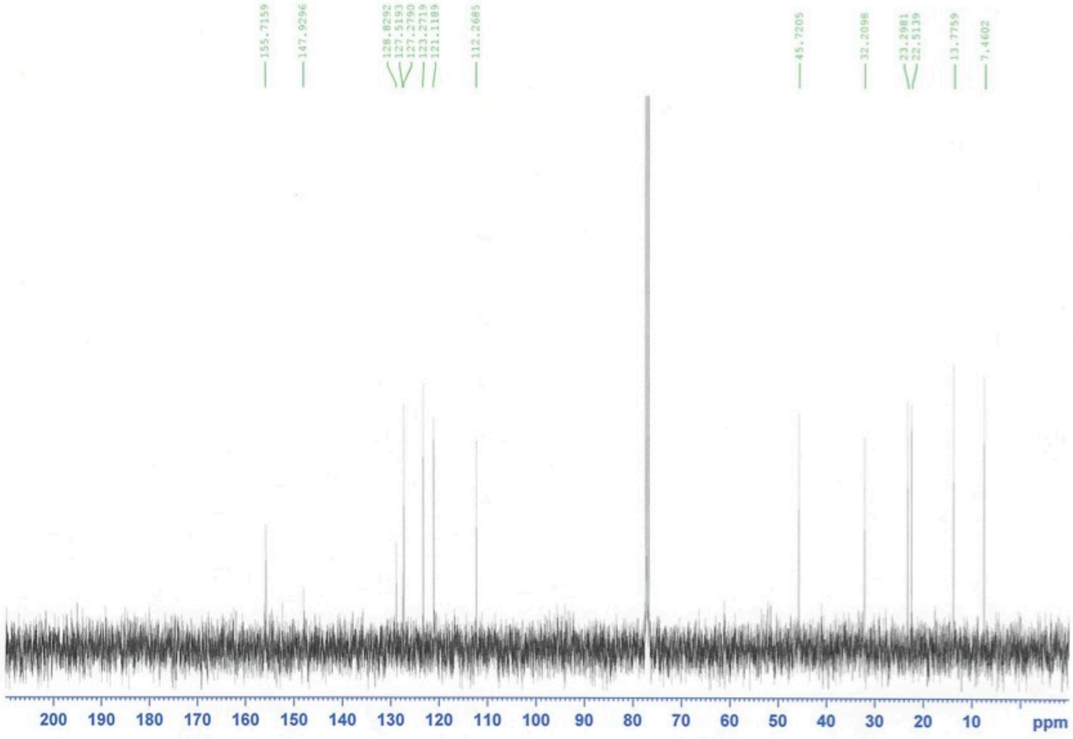

^{1}H NMR (400 MHz) and ^{13}C NMR (101 MHz) spectra of 3-butyl-2-(ethylsulfonyl)benzo[*b*]furan (**8**) ($CDCl_3$)

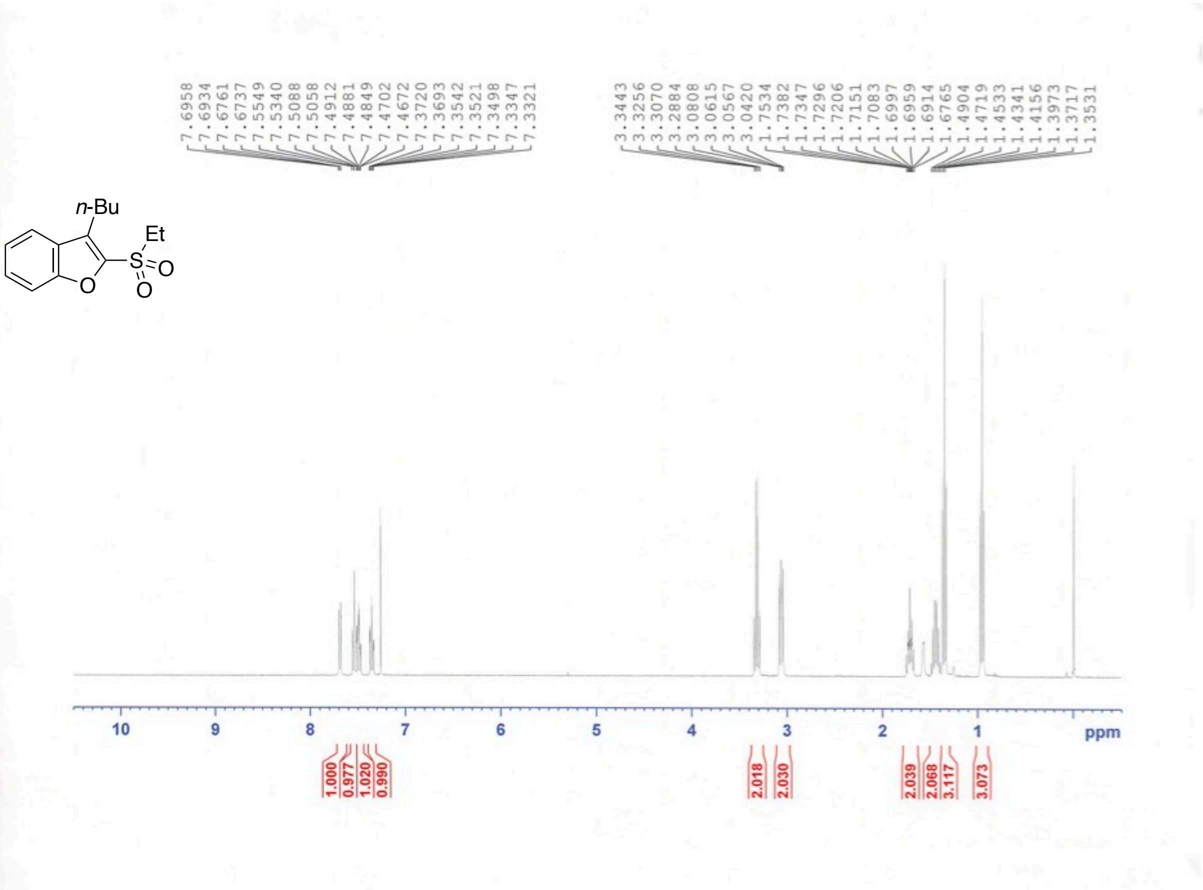

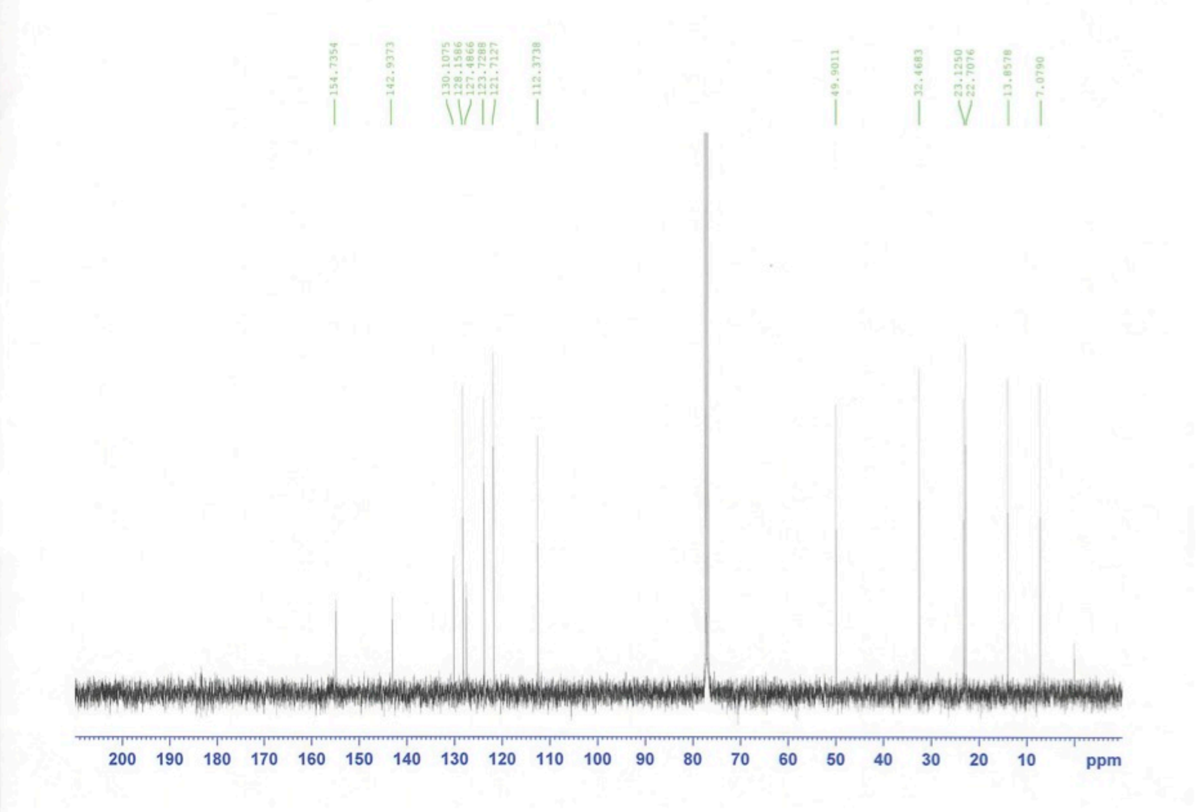

^{1}H NMR (400 MHz) and ^{13}C NMR (101 MHz) spectra of 3-butyl-2-(2-(ethylthio)-3-methoxyphenoxy)benzo[*b*]furan (**12**) ($CDCl_3$)

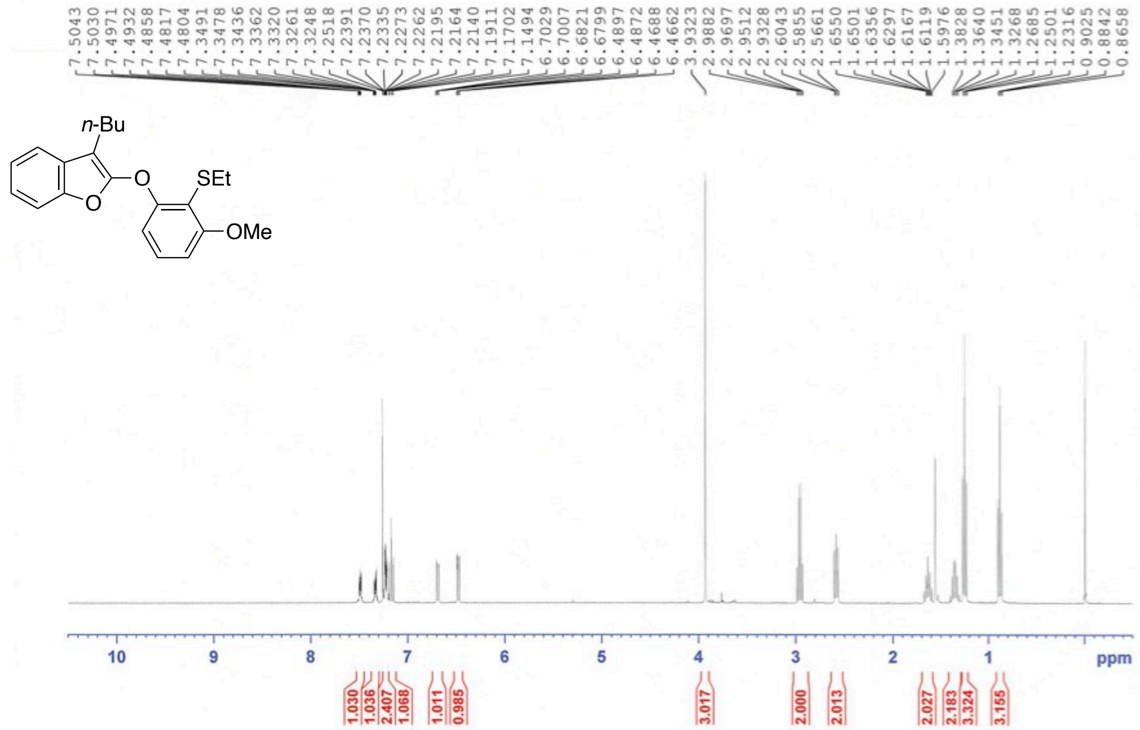

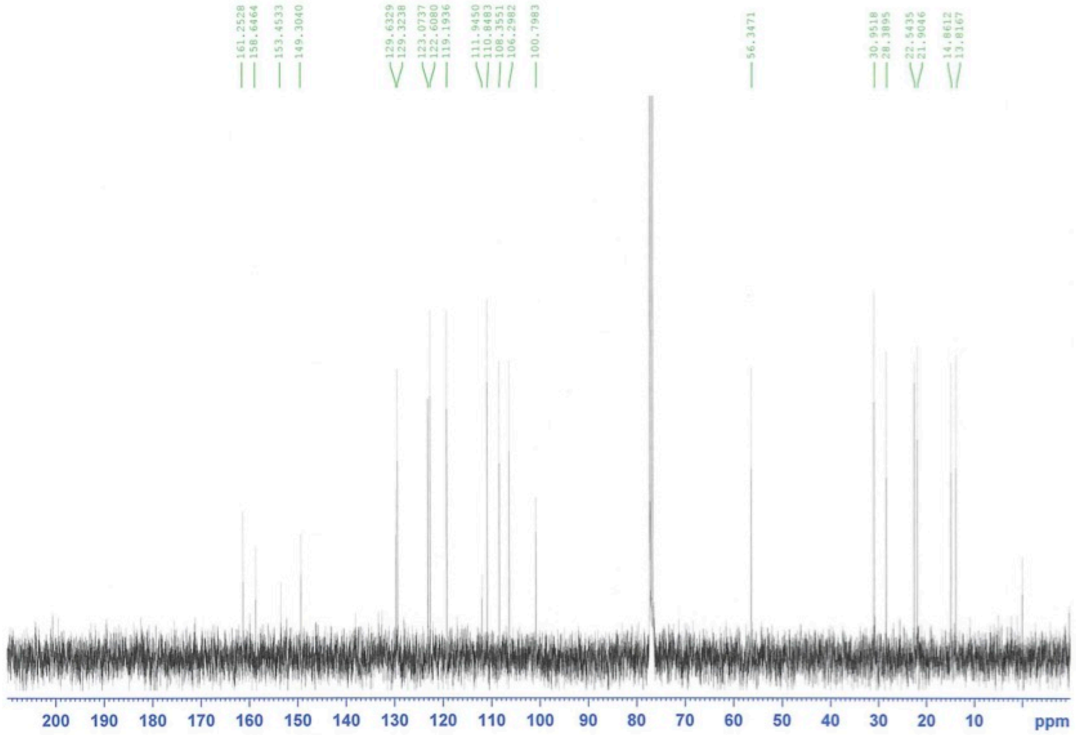

^{1}H NMR (400 MHz) and ^{13}C NMR (101 MHz) spectra of hex-1-yn-1-yl 4-tolyl sulfide ($CDCl_3$)

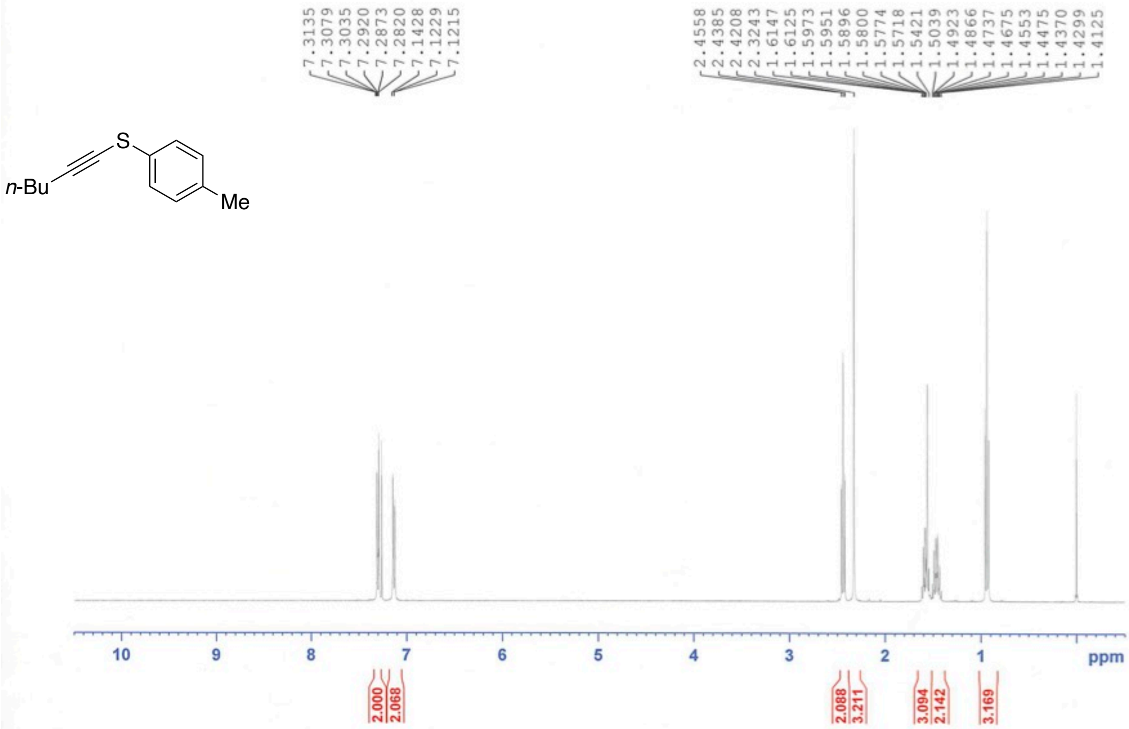

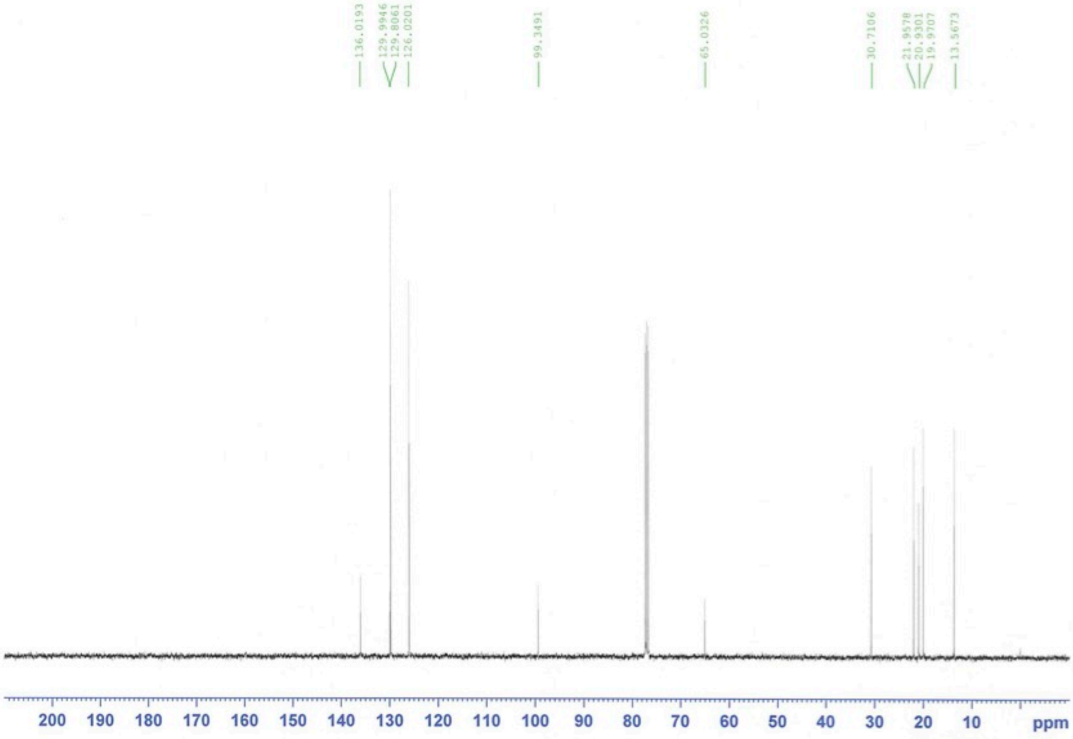

^1H NMR (400 MHz) and ^{13}C NMR (101 MHz) spectra of 4-bromophenyl hex-1-yn-1-yl sulfide ($CDCl_3$)

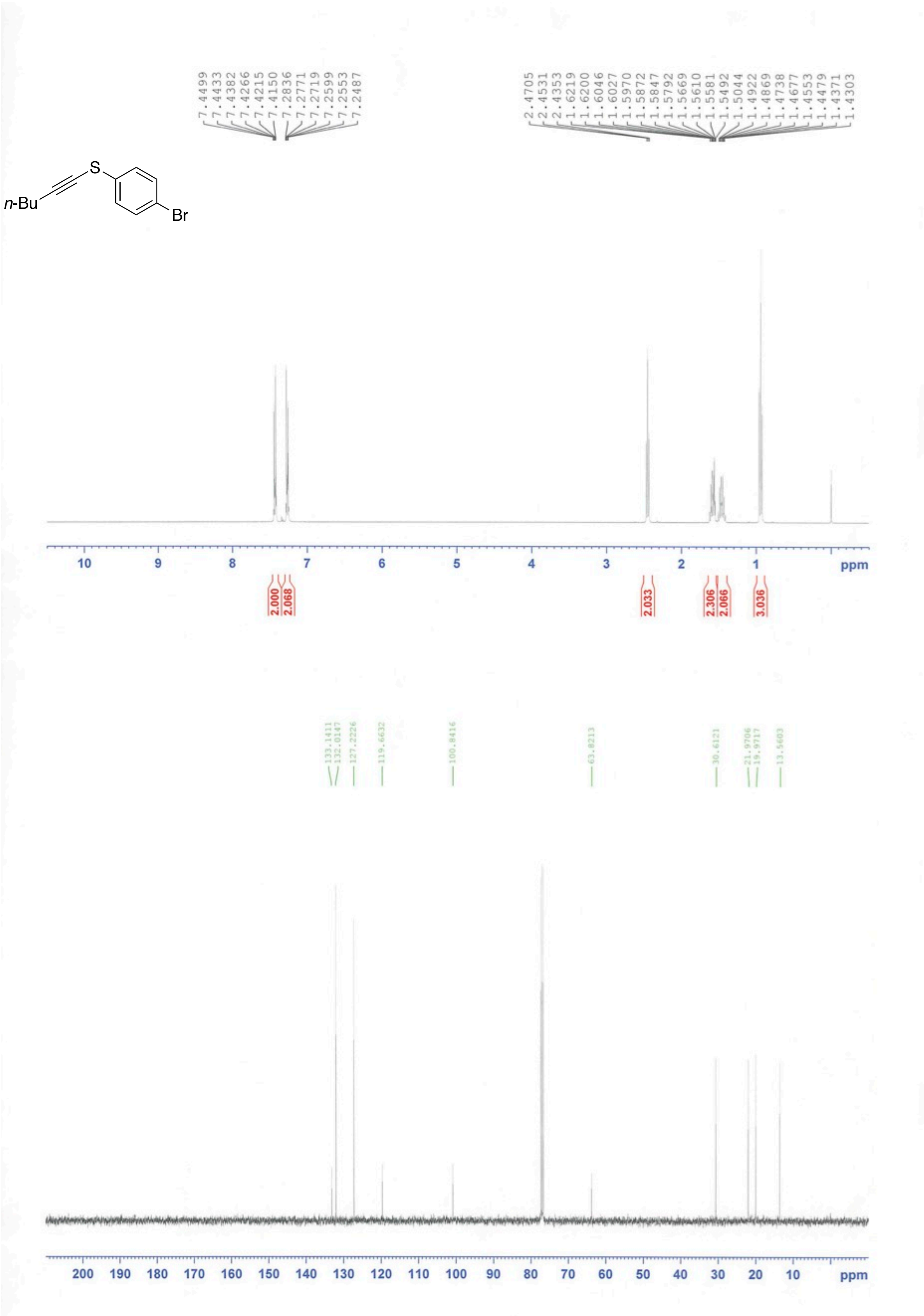

^{1}H NMR (400 MHz) and ^{13}C NMR (101 MHz) spectra of 6-chlorohexyl hex-1-yn-1-yl sulfide ($CDCl_3$)

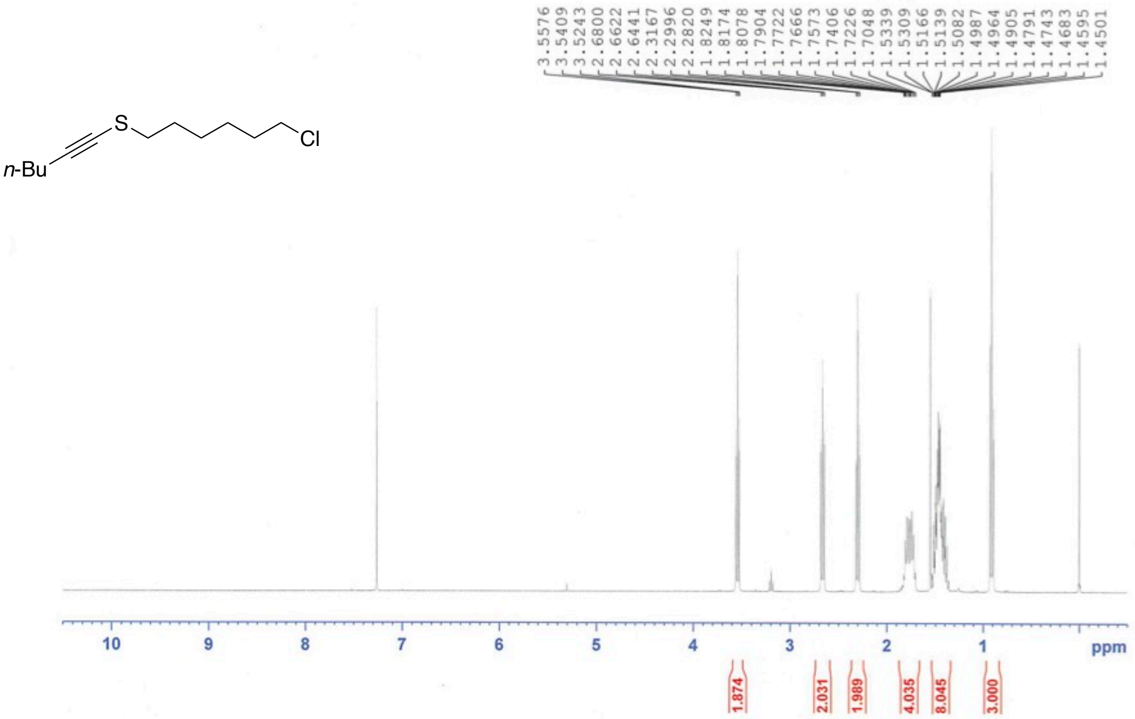

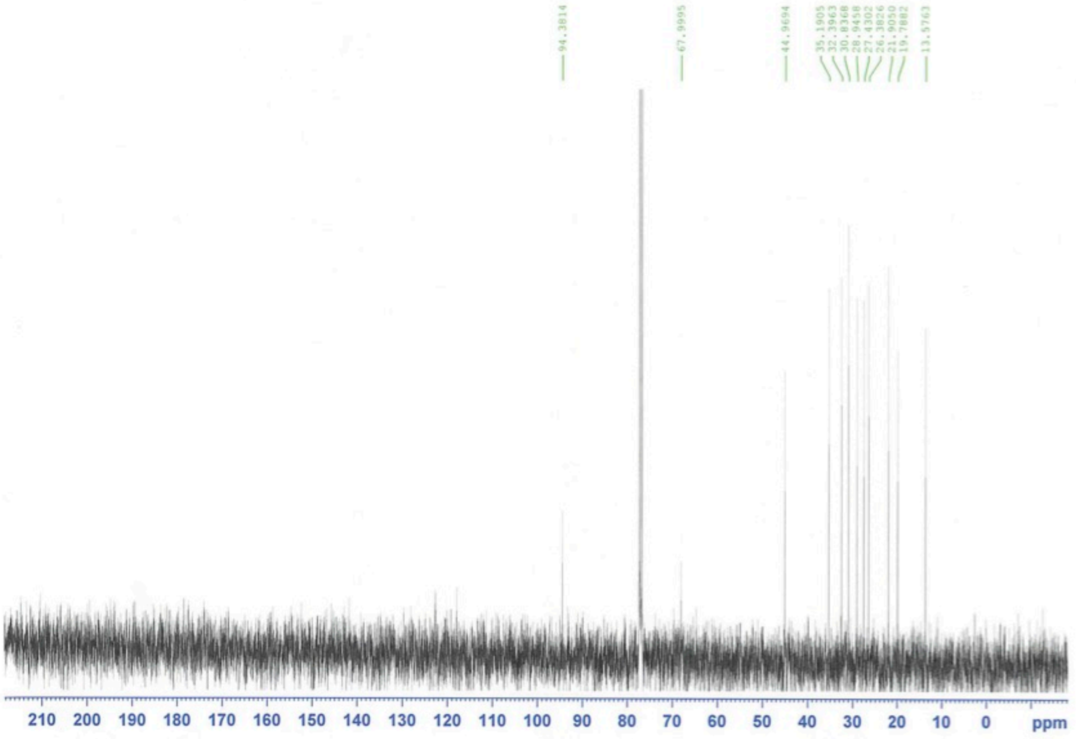

^{1}H NMR (400 MHz) and ^{13}C NMR (101 MHz) spectra of (4-(ethylsulfinyl)but-3-yn-1-yl)benzene (**2b**) ($CDCl_3$)

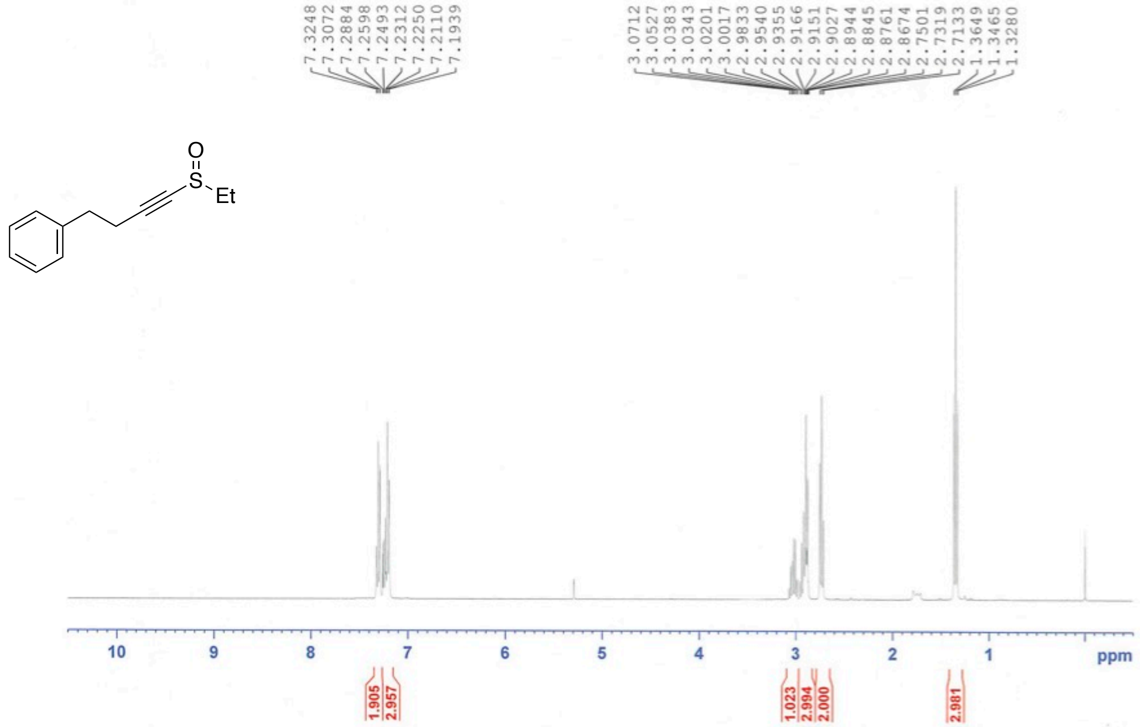

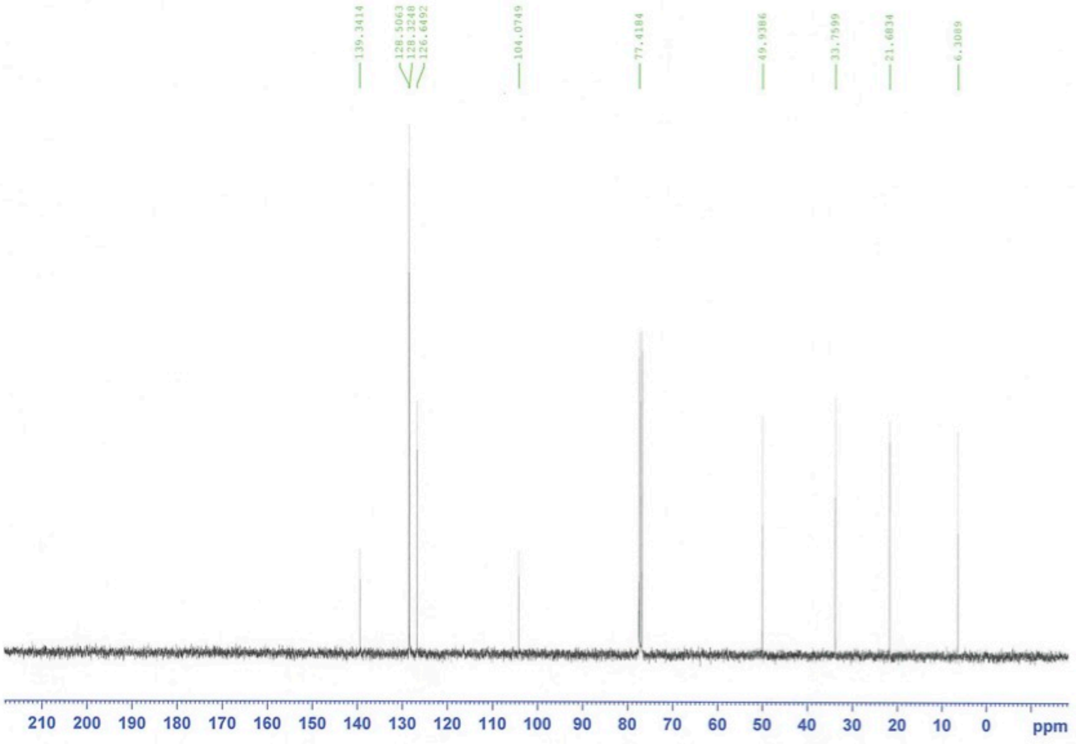

^{1}H NMR (400 MHz) and ^{13}C NMR (101 MHz) spectra of 4-(ethylsulfinyl)but-3-yn-1-yl 4-methylbenzoate.(**2c**) ($CDCl_3$)

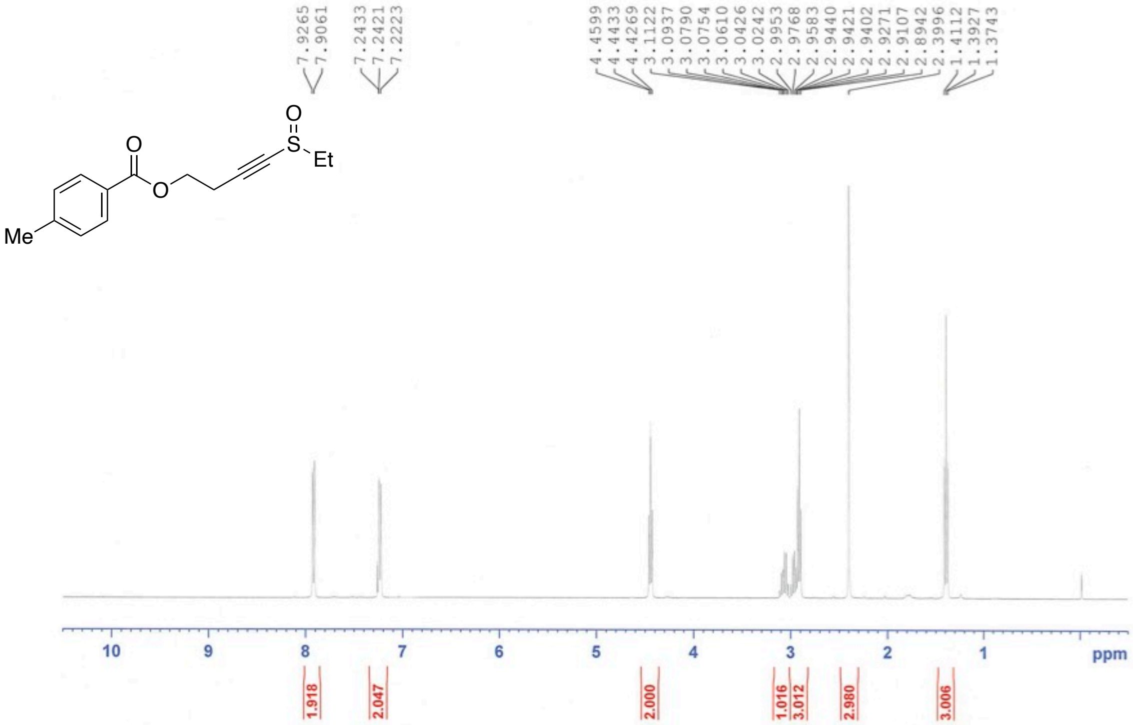

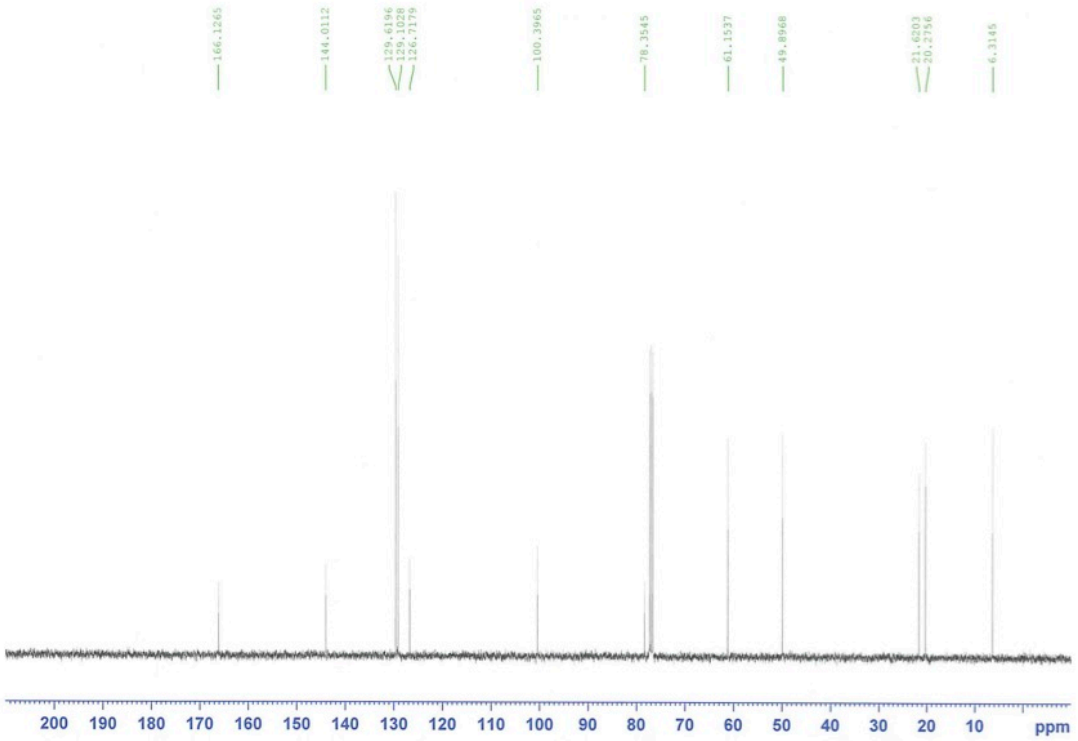

^{1}H NMR (400 MHz) and ^{13}C NMR (101 MHz) spectra of (2-((3-(ethylsulfinyl)prop-2-yn-1-yl)oxy)ethyl)benzene (**2d**) ($CDCl_3$)

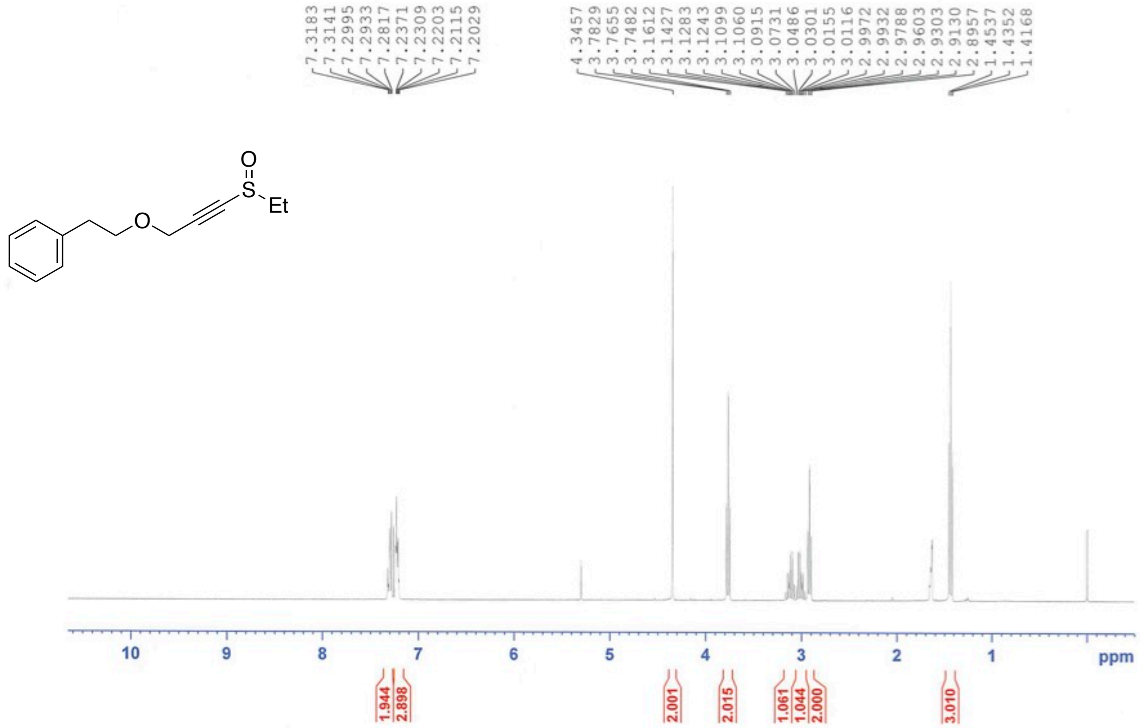

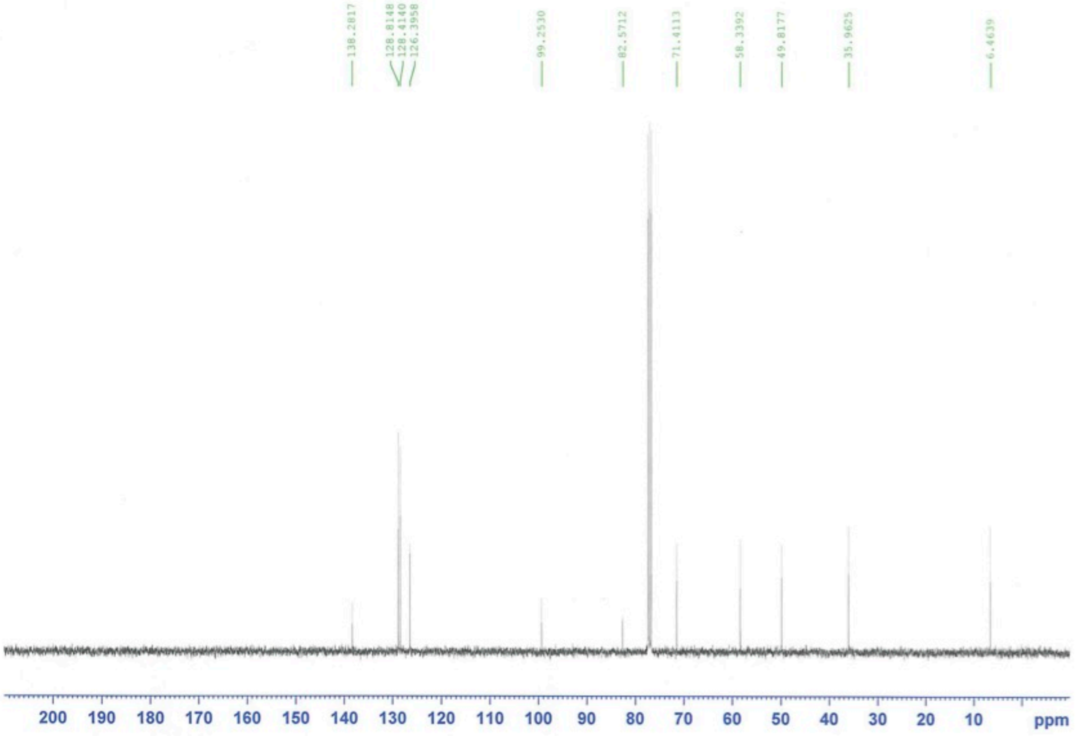

^{1}H NMR (400 MHz) and ^{13}C NMR (101 MHz) spectra of 1-((ethylsulfinyl)ethynyl)-4-methylbenzene (**2e**) ($CDCl_3$)

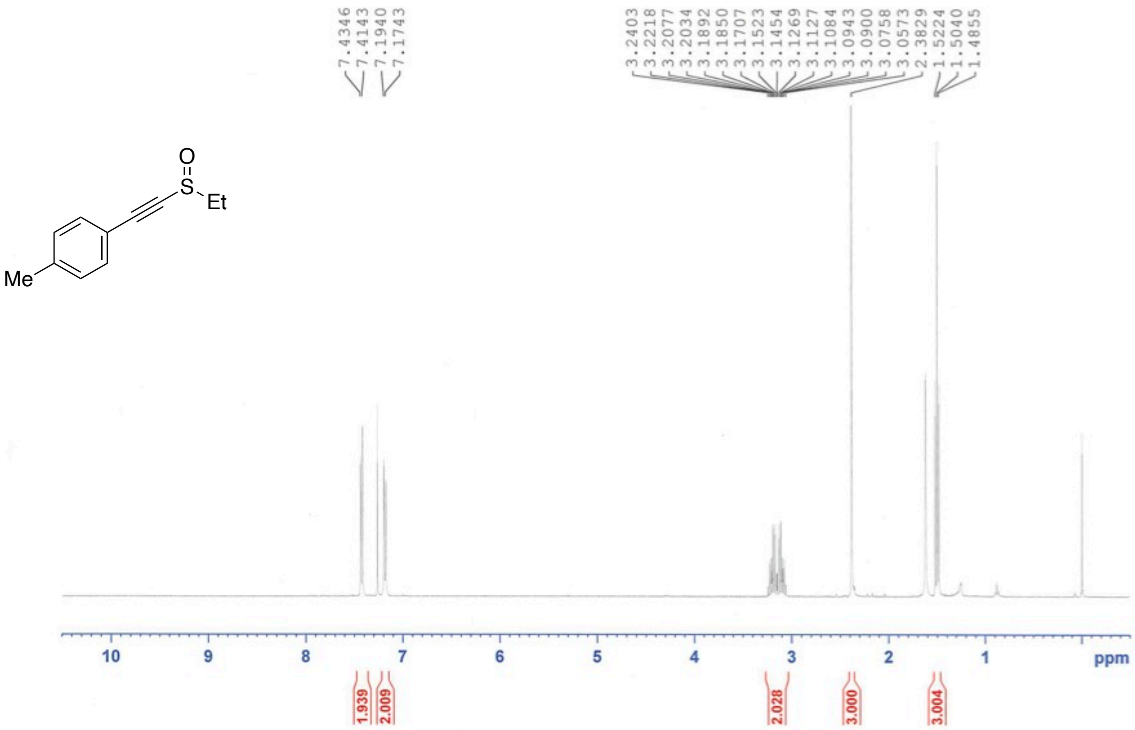

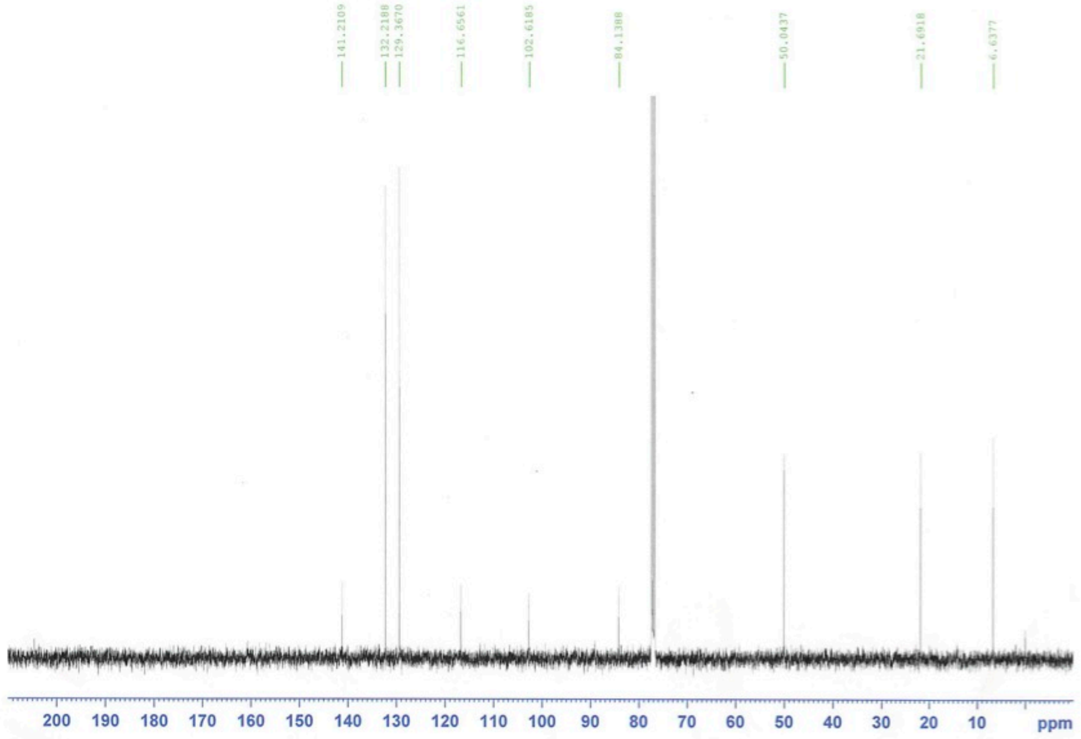

^{1}H NMR (400 MHz) and ^{13}C NMR (101 MHz) spectra of 1-chloro-4-((ethylsulfinyl)ethynyl)benzene (**2f**) ($CDCl_3$)

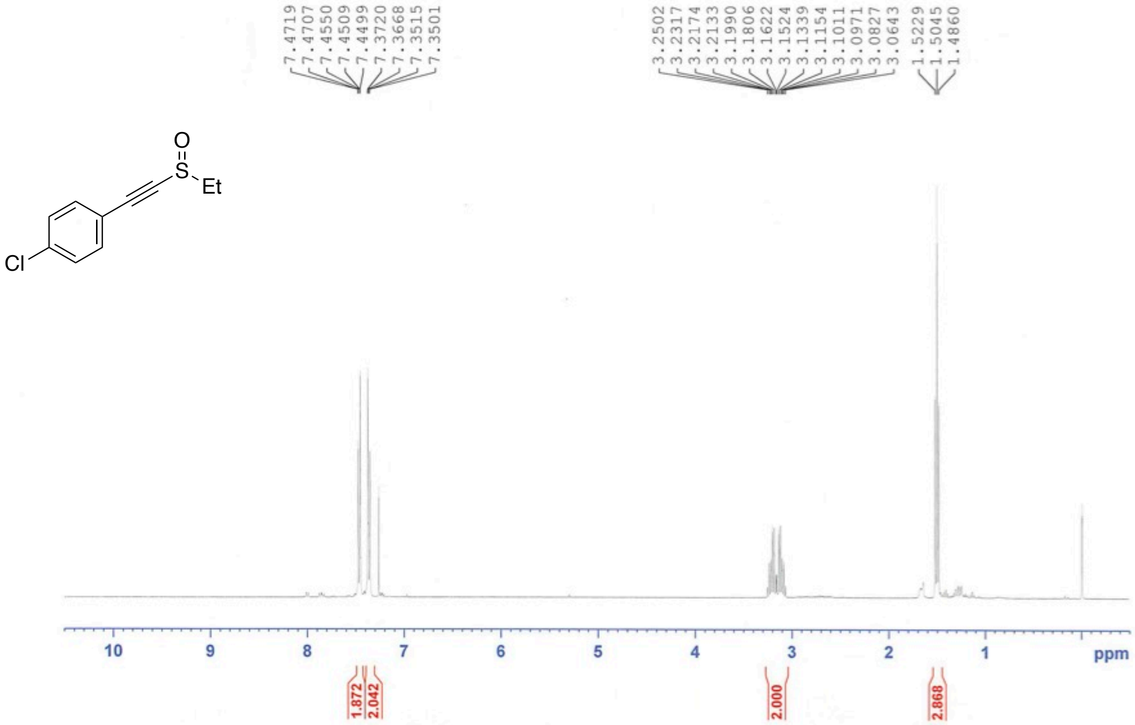

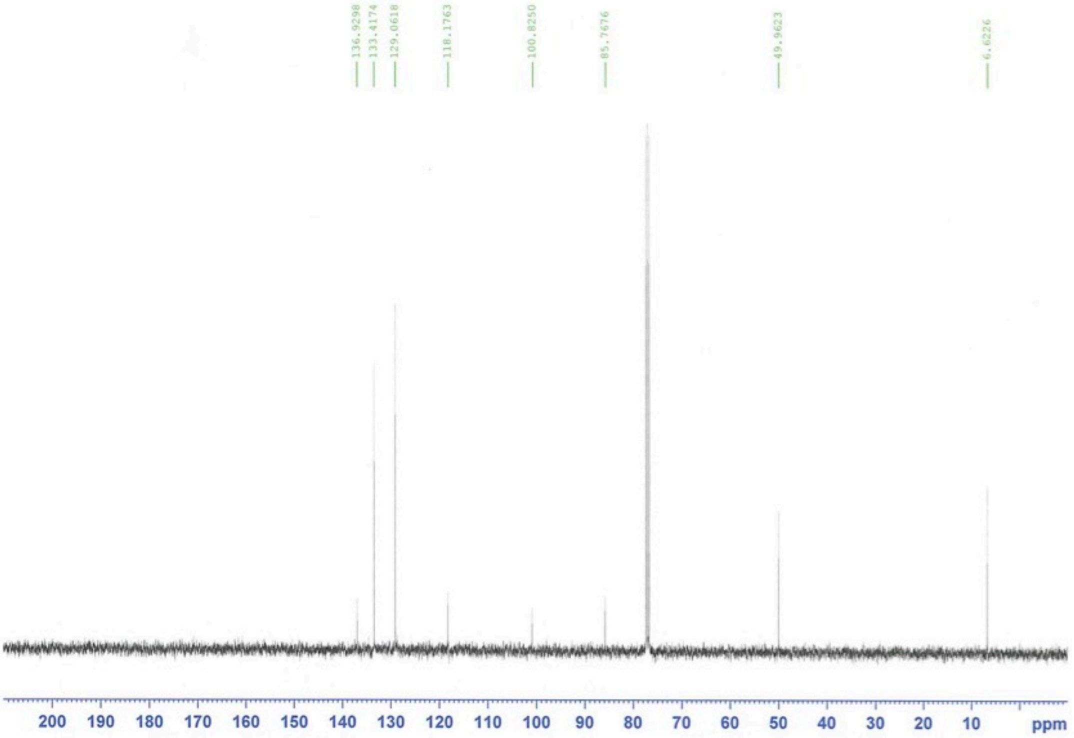

^{1}H NMR (400 MHz) and ^{13}C NMR (101 MHz) spectra of 1-(hex-1-yn-1-ylsulfinyl)-4-methylbenzene (**2g**) ($CDCl_3$)

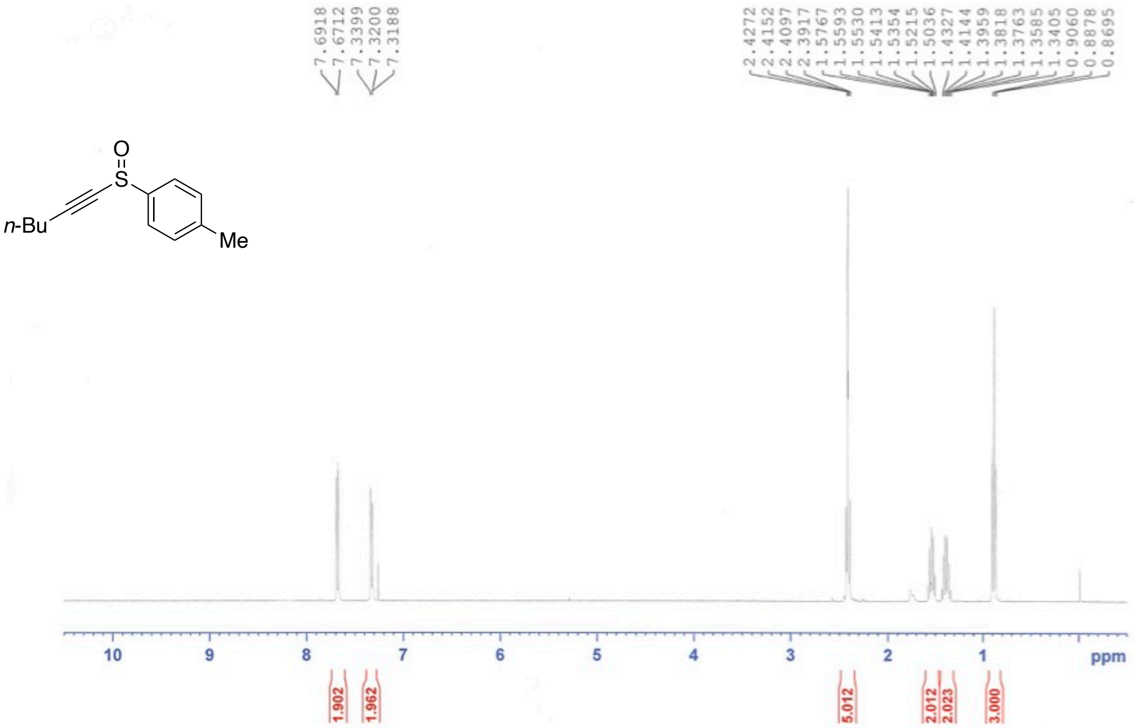

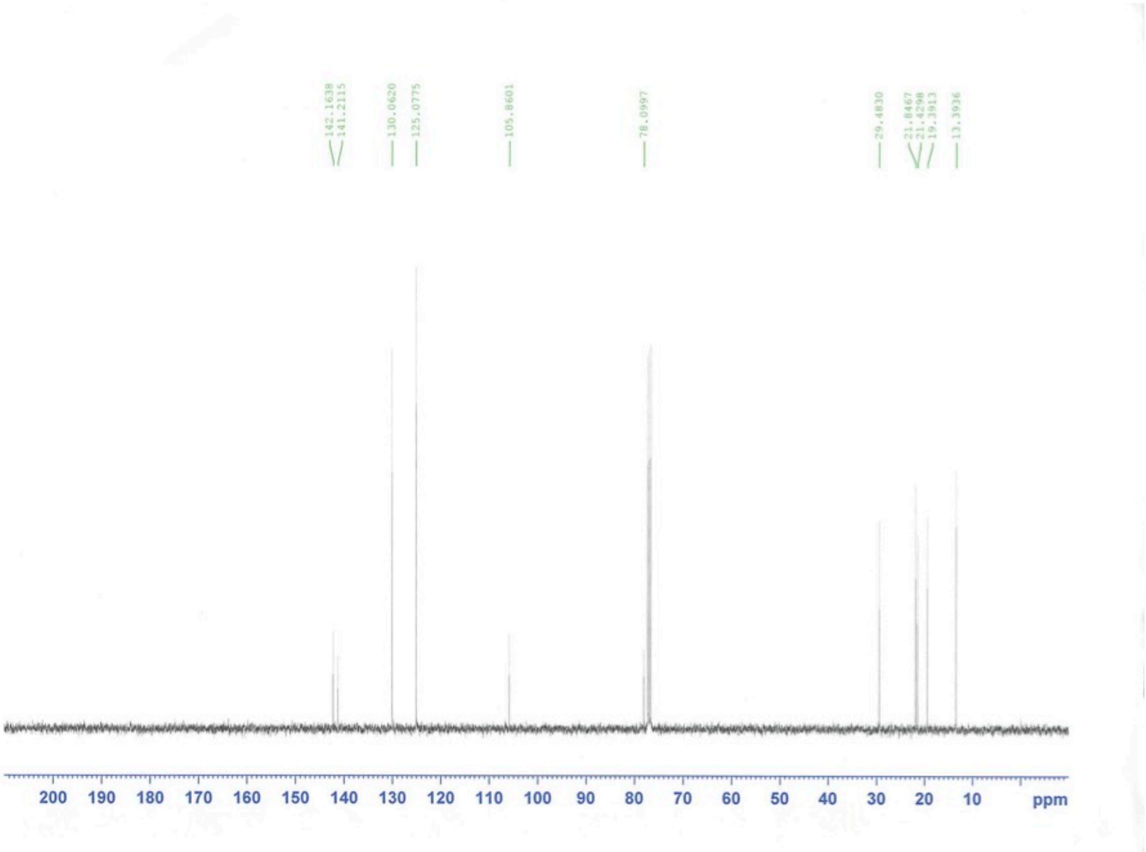

^{1}H NMR (400 MHz) and ^{13}C NMR (101 MHz) spectra of 1-bromo-4-(hex-1-yn-1-ylsulfinyl)benzene (**2h**) ($CDCl_3$)

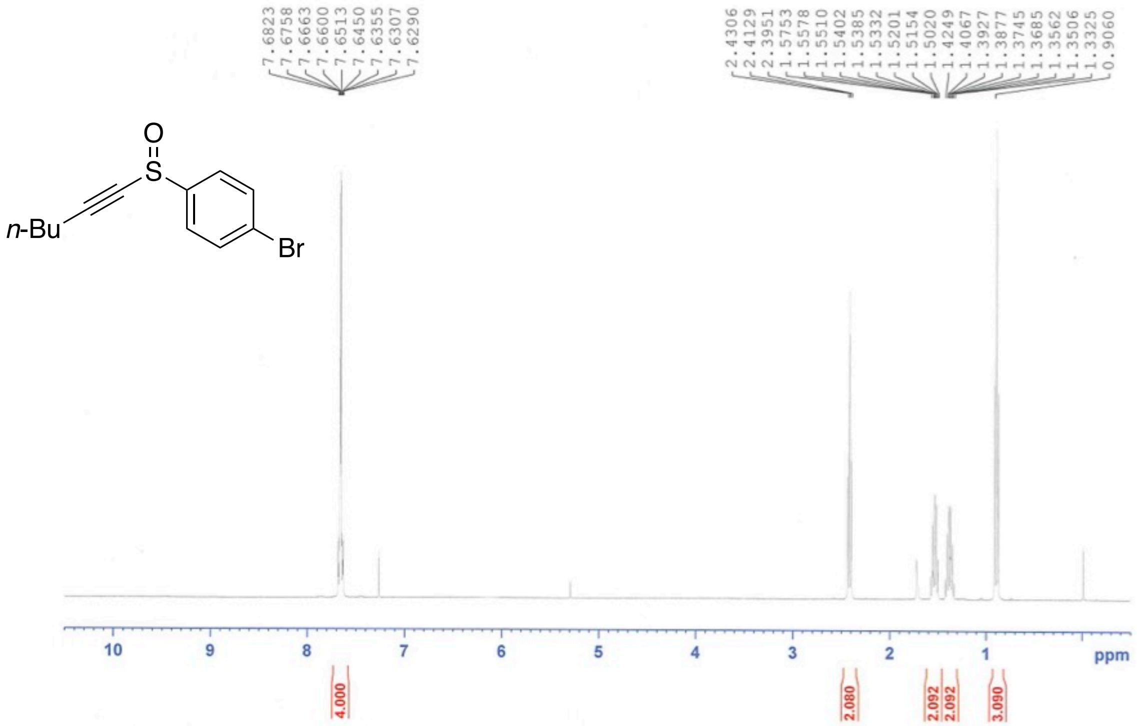

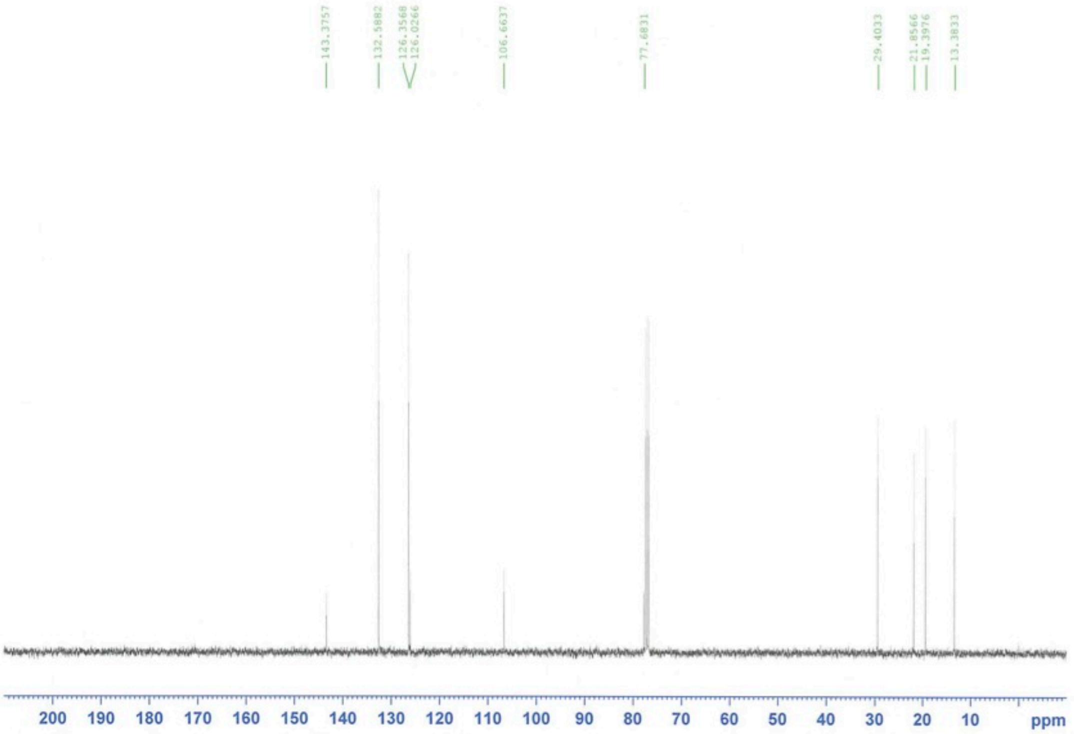

^{1}H NMR (400 MHz) and ^{13}C NMR (101 MHz) spectra of ((hex-1-yn-1-ylsulfinyl)methyl)benzene (**2j**) ($CDCl_3$)

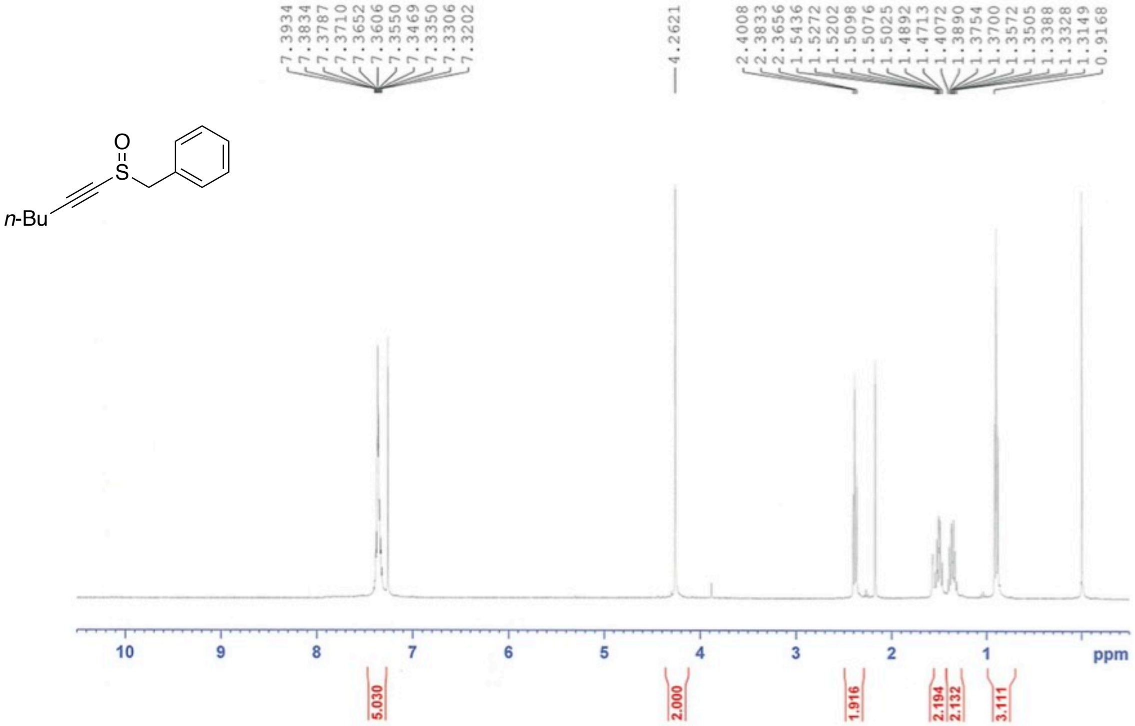

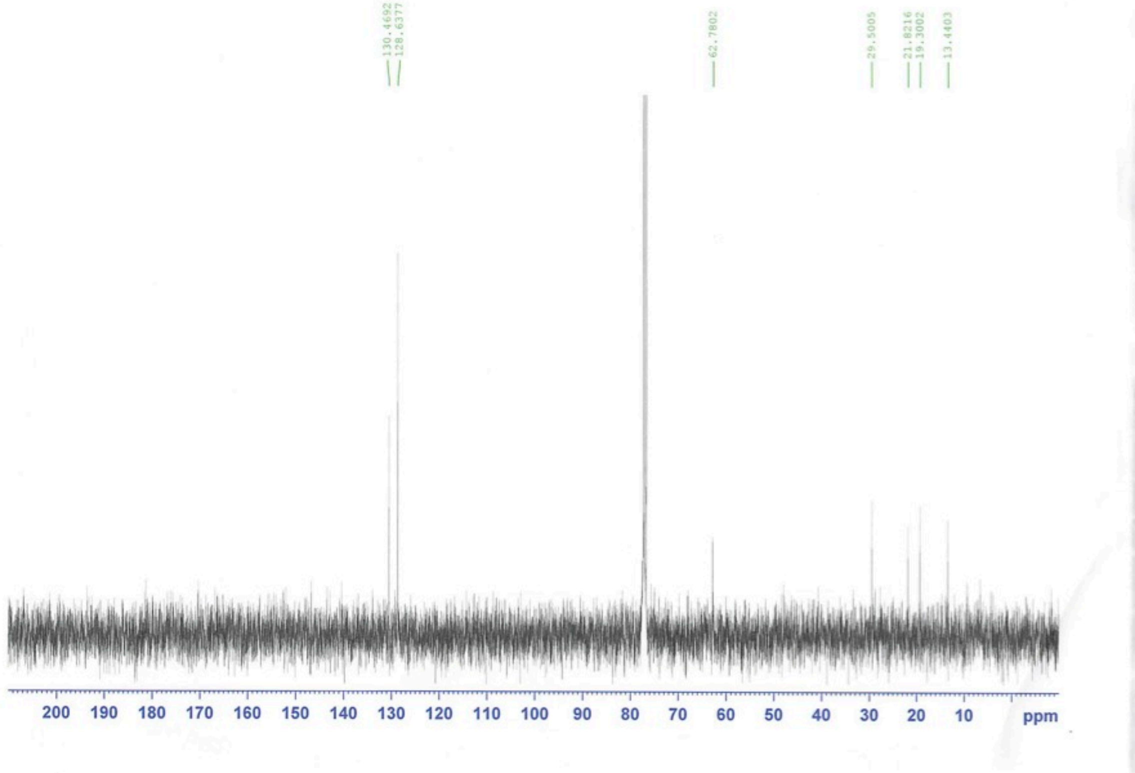

^{1}H NMR (400 MHz) and ^{13}C NMR (101 MHz) spectra of 1-((6-chlorohexyl)sulfinyl)hex-1-yne (**2k**) ($CDCl_3$)

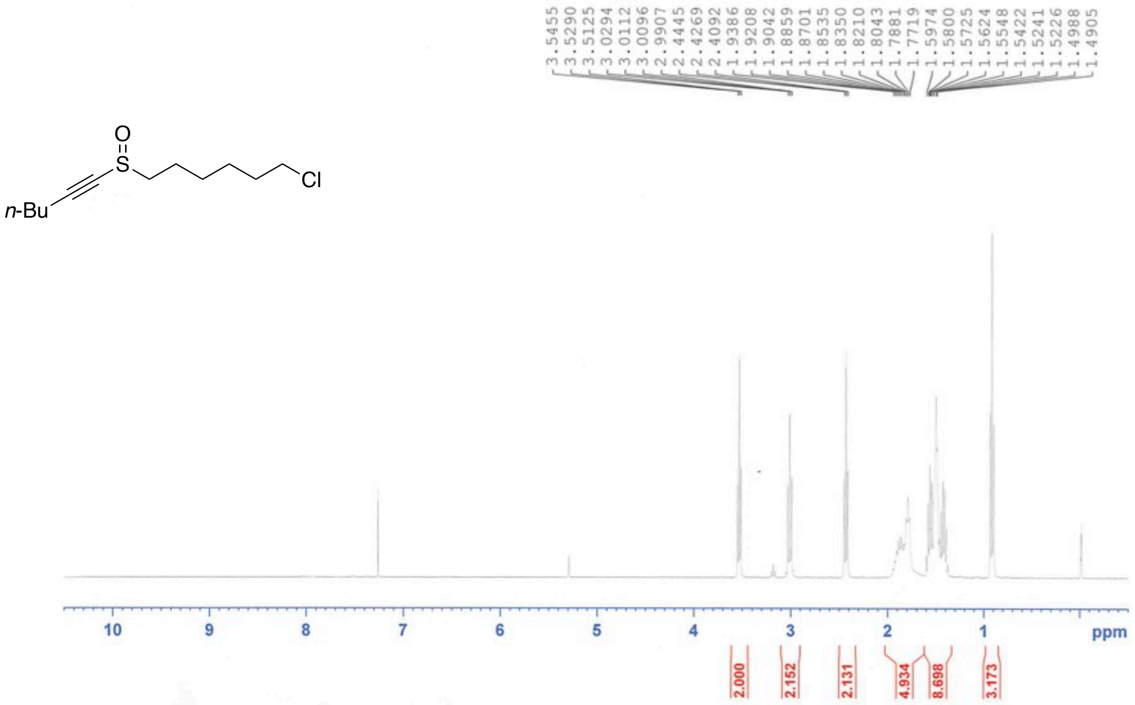

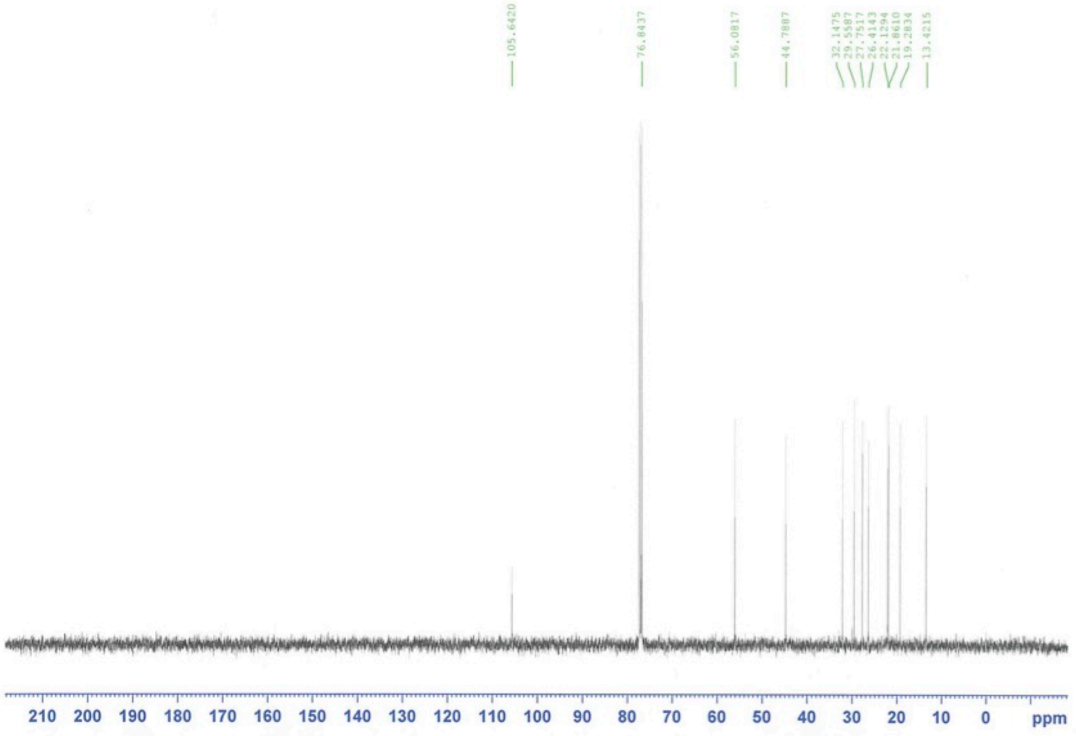

Supplement: RA-013-D2RA07856B-s001 [file RA-013-D2RA07856B-s001.pdf]
